# Design, synthesis and *in silico* molecular modelling studies of 2-Hydrazineyl-2-oxoethyl-4-(1*H*-pyrrol-1-yb) benzoate derivatives: a potent dual DHFR and ENR-reductase inhibitors with antitubercular, antibacterial and cytotoxic potential

**DOI:** 10.1371/journal.pone.0323702

**Published:** 2025-05-19

**Authors:** Mater H. Mahnashi, Prem Kumar Santhemavathuru Ramachandraiah, Ahmed Abdullah Al Awadh, Ibrahim Abdullah Almazni, Yahya I. Asiri, Ibrahim Ahmed Shaikh, Basheerahmed Abdulaziz Mannasaheb, Sravanthi Avunoori, Aejaz Abdullatif Khan, Shrinivas D. Joshi

**Affiliations:** 1 Department of Pharmaceutical Chemistry, College of Pharmacy, King Khalid University, Abha, Saudi Arabia; 2 Department of Pharmaceutical Quality Assurance, Sree Siddaganga College of Pharmacy, Tumkur, Karnataka, India; 3 Department of Clinical Laboratory Sciences, Faculty of Applied Medical Sciences, Najran University, Najran, Saudi Arabia; 4 Department of Pharmacology, College of Pharmacy, King Khalid University, Abha, Saudi Arabia; 5 Department of Pharmacology, College of Pharmacy, Najran University, Najran, Saudi Arabia; 6 Department of Pharmacy Practice, College of Pharmacy, AlMaarefa University, Riyadh, Saudi Arabia; 7 Novel Drug Design and Discovery Laboratory, Department of Pharmaceutical Chemistry, SET’s College of Pharmacy, Dharwad, Karnataka, India; 8 Department of General Science, Ibn Sina National College for Medical Studies, Jeddah, Saudi Arabia; Vignan Pharmacy College, INDIA

## Abstract

The current research includes the study of 28 new 2-hydrazineyl-2-oxoethyl-4-(1*H*-pyrrol-1-yl) benzoate derivatives as antitubercular, antibacterial, and enoyl-ACP reductase enzyme inhibitors. SYBYL-X.2.0 was used to investigate the molecular docking of ENR-ACP reductase/InhA in complex with 1-cyclohexyl-*N*-(3,5-dichlorophenyl)-5-oxopyrrolidine-3-carboxamide (PDB ID:4TZK) and MtDHFR in complex with methotrexate (PDB ID:1DF7). All of the reported derivatives have two or more H-bonding interactions with TYR158 and the cofactor NAD + , which fit snugly into InhA’s binding pocket, with MIC values of 0.8–3.12 µg/mL, 0.4–3.12 µg/mL, and 1.6–12.5 µg/mL [4(a-e), 5(a-p), 6(a-e)]. Also, the molecular H-bonding interactions of reported molecules with amino acids ARG32 and ARG60 of MtDHFR showed the interaction of molecules with targeted site. All of the reported compounds showed good activity against *M. tuberculosis* H_37_Rv, Gram-negative *E.coli*, and Gram-positive *S. aureus*, respectively. Compounds 5b and 6d showed highest antitubercular activity with the MIC value of 0.8 µg/mL. InhA inhibition was good to moderate in the tested compounds, with IC_50_ inhibition ranging from 9 to 51% at 50μM; and MtDHFR inhibition was good with IC_50_ values ranging from 23 to 153 µM compared to trimethoprim at 92 µM. The most potent compounds exhibiting dual enzyme inhibition were further evaluated for cytotoxicity in mammalian cells using the human lung cancer cell line A549. These compounds demonstrated significant cytotoxic effects, with IC_50_ values ranging from 255 to 319 µg/mL. In comparison, the standard antitubercular drug isoniazid exhibited an IC_50_ value greater than 450 µg/mL, while the anticancer drug cisplatin showed an IC_50_ value of 9.9 µg/mL. These molecules represent excellent future therapeutic possibilities with potential use in the biological and medical sciences due to the compounds’ pronounced docking properties and biological activity.

## 1. Introduction

Tuberculosis (TB) is a persistent infectious disease primarily affecting the lungs, caused by the bacterial pathogen *Mycobacterium tuberculosis* [1]. The disease is spread through the inhalation of aerosolized droplets expelled by TB-infected individuals during coughing, sneezing, or spitting [2]. TB is a preventable and curable disease, yet it is estimated that one-quarter of the global population has been infected with the TB bacteria [2]. Individuals with weakened immune systems are more susceptible to TB infection compared to those with a healthy immune system [3]. The World Health Organization (WHO) report for 2023 indicates a significant recovery in the number of individuals diagnosed with TB and receiving treatment in 2022, following the disruptions caused by the COVID-19 pandemic [4]. However, TB remained the second leading cause of death from a single infectious agent globally in 2022, after COVID-19, and the global TB elimination targets have either been missed or remain off track [4]. According to the TB report for India, nearly 33% or 8.4 lakh of the 25.5 lakh TB cases reported in 2023 were from the private healthcare sector [4]. The estimated incidence of TB in 2023 increased slightly to 27.8 lakh cases from the previous year’s estimate of 27.4 lakh [4]. India has reached its 2023 target of initiating treatment in 95% of patients diagnosed with the infection [5]. Despite setting ambitious goals to eliminate tuberculosis by 2025, India has faced challenges in meeting these targets, and the number of cases and deaths recorded in 2023 fell short of the targets set by the country [5,6]. Consequently, there is a pressing need for the development of novel chemotherapeutic agents that are safer, more effective, and work through a variety of mechanisms to combat this persistent public health threat [5,6]. Enoyl Acyl Carrier Protein (ACP) Reductase (InhA) is an enzyme involved in fatty acid synthesis mainly mycolic acid biosynthesis. It is a part of Tyrosine-dependent oxidoreductase also known as a short dehydrogenase/reductase family, especially to NADH-dependent enoyl ACP reductase [7]. It catalyses trans double bond reduction which is linked to a carbonyl group of an intermediate that is covalently linked to an acyl carrier protein in the FAS-II pathway [8]. InhA associated enoyl ACP reductase, an enzyme responsible for fatty acid synthesis has been one of the best verified targets for the development of anti-TB drugs or InhA inhibitors that are found to be active only against sensitive TB but not against Multi-Drug Resistant (MDR) TB due to Kat G mutant selection [9]. Jacob and co-workers identified InhA that catalyses the final enzymatic step in the FAS-II pathway as an effective target [10]. InhA plays a central role in the biosynthesis of mycolic acids, which are essential components of the Mycobacterium tuberculosis cell wall. Inhibition of the InhA enzyme disrupts the mycolic acid synthesis pathway, leading to the weakening and eventual lysis of the bacterial cell wall. This makes InhA a prime target for the development of new anti-TB drugs, as compounds that can effectively inhibit this enzyme can potentially kill or prevent the growth of *M. tuberculosis*. Hence, it is a crucial target in the development of novel anti-tuberculosis (TB) therapies.

Dihydrofolate reductase (DHFR) is another crucial enzyme that catalyzes the reduction of dihydrofolate to tetrahydrofolate which couples with thymidylate synthase in the reductive methylation of deoxyuridine to deoxythymidine. Tetrahydrofolate cofactor deficiencies brought on by the suppression of DHFR function result in cell death. As a key target for the development of chemotherapeutic agents against bacterial and parasite diseases as well as TB, DHFR inhibition has long been recognized. Methotrexate and trimethoprim are examples of DHFR inhibitors used in clinical practice.

The achievement of a drug is determined not only by its efficacy but also by an acceptable Absorption, Distribution, Metabolism, Excretion, and Toxicity (ADMET) profile [11]. ADMET studies are crucial in drug discovery because they help determine the safety and efficacy of a drug candidate. By conducting ADMET studies in combination with in vivo and in vitro assessments early in the drug discovery process, researchers can identify and address potential issues related to toxicity and efficacy, improving the chances of success in later stages of development [12]. This helps in making informed decisions about which drug candidates to move forward with, ultimately saving time and resources.

The pyrrole heterocyclic ring template possesses multiple pharmacophores, providing a means for the generation of a library of diverse lead molecules [13]. Due to its significant pharmacological profile, pyrrole and its analogues have drawn considerable attention from researchers and chemists worldwide, leading to extensive exploration for the benefit of humanity [14]. The pyrrole moiety is an essential structural motif in functional compounds, natural phytomolecules, and pharmaceutical drugs [15]. Increasingly, efficient synthetic strategies towards pyrroles have emerged, wherein various effective building blocks are developed, and these synthons enable the synthesis of pyrroles with unique compositional features [15].

Our laboratory for Novel Drug Design and Discovery has been conducting studies on the potential of dihydrofolate reductase (DHFR) and enoyl-acyl carrier protein (enoyl-ACP) reductase as molecular targets for anti-tuberculosis (anti-TB) therapies. Specifically, we have been investigating the use of pyrrole pharmacophoric scaffolds that can inhibit both DHFR and enoyl-ACP reductase [16–18]. The rationale behind this approach is to develop new compounds that can effectively inhibit both enzymes, thereby demonstrating enhanced anti-tuberculosis activity. DHFR and enoyl-ACP reductase are critical enzymes involved in essential metabolic pathways of *M. tuberculosis*, the causative agent of tuberculosis. Targeting these two enzymes simultaneously can potentially result in a more potent and comprehensive disruption of the bacterial metabolism, leading to improved therapeutic outcomes. By exploring pyrrole-based scaffolds, we aim to leverage the unique pharmacological properties and versatility of this heterocyclic moiety to design novel anti-TB agents. Pyrroles have shown tremendous potential as building blocks for the generation of diverse lead compounds with promising biological activities, including antimicrobial, anti-inflammatory, and anti-cancer properties.

The pharmacophore hybridization approach is a strategic technique used in drug design. It allows researchers to combine key pharmacological components into a single molecular structure. Literature suggests that pyrazoles and hydrazides possess potential as antimicrobial agents [19]. Likewise, 1,4-Dihydroindeno[1,2-c]pyrazole, a novel pseudoazulenic structure with two fused five-membered rings, was first synthesized by Boyd in 1965. Recently, the indenopyrazole pharmacophore has garnered significant interest due to its diverse biological activities. The imidazo[2,1-b]thiazole scaffold is another chemical motif that has been extensively studied by medicinal chemists. This is because it has shown a variety of important biological activities, including anti-cancer, blood pressure-lowering, antimicrobial, and anti-inflammatory effects. Interestingly, this imidazo[2,1-b]thiazole core is found in the structure of the drug levamisole, which is used as an anti-parasitic and to modulate the immune system [20,21].

The ongoing studies in our laboratory are focused on the systematic optimization of pyrrole-based inhibitors, evaluating their potency, selectivity, and ADMET profiles. This multi-pronged approach, combining computational predictions and in vitro/in vivo assessments, aims to expedite the discovery and development of novel anti-tuberculosis therapeutics that can address the evolving challenges posed by drug resistance and the global burden of this infectious disease.

In this study, we present the design and synthesis of 28 new pyrrole scaffold-containing compounds that act as dual-target inhibitors of dihydrofolate reductase (DHFR) and enoyl-acyl carrier protein (enoyl-ACP) reductase. These dual-inhibitor molecules have the potential to overcome the limitations associated with the use of separate drugs in combination chemotherapy treatments for tuberculosis. The development of these dual-target inhibitors is a strategic approach to address the issues related to toxicity, drug-drug interactions, and/or pharmacokinetic drawbacks that can arise when administering multiple medications concurrently. Furthermore, the cost of a single dual-inhibitor drug could be lower than the combined cost of two separate treatments, and it may also improve patient compliance. The successful development of these pyrrole-based dual-target inhibitors could lead to the identification of promising anti-tuberculosis drug candidates with improved therapeutic potential and better pharmacological properties.

## 2. Results and discussion

According to methodology, the compounds **4(a-g)** were prepared from the key intermediate **3** by reacting with different anhydrides in presence of ethanol, **5(a-p)** were prepared by reacting the compound **3** with substituted acetophenones in presence of ethanol and few drops of glacial acetic acid, **6(a-e)** were prepared by reacting compound **3** with substituted benzaldehydes in presence of ethanol and glacial acetic acid. The completion of reactions was monitored by TLC and the reaction products were purified using column chromatography. The reactions afforded the reaction mixtures in good yield. The structures of the synthesised compounds were confirmed by IR, ^1^HNMR, ^13^C NMR and Mass spectroscopic methods.

### 2.1. Molecular docking

**Fig 1:**
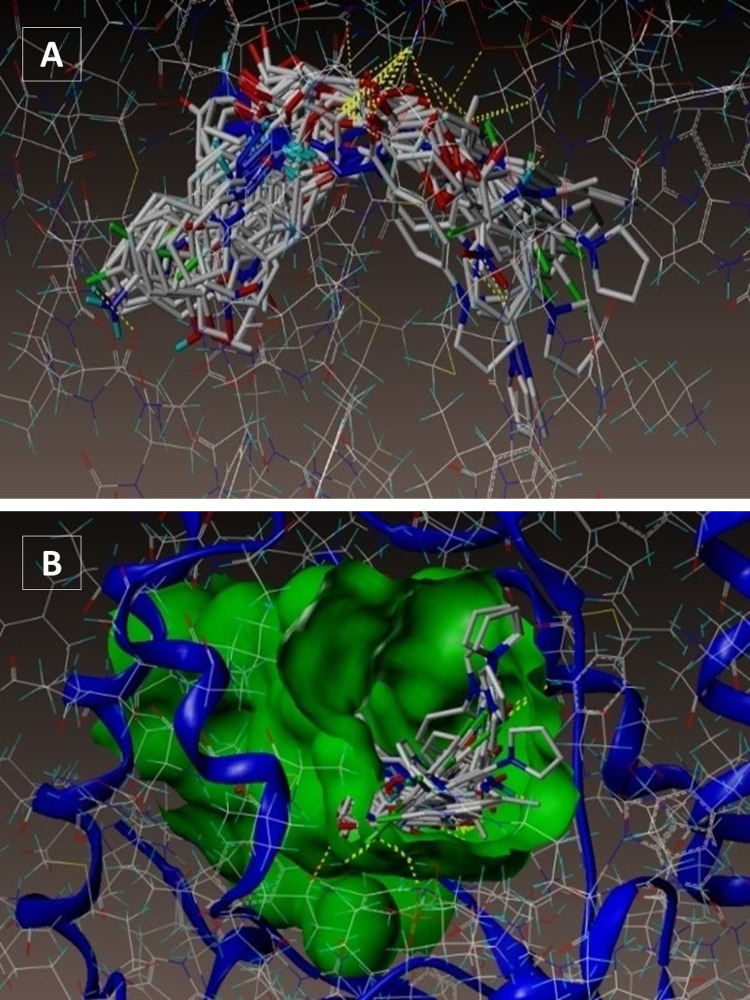
(A-B): Docked mode of all the 28 synthesized compounds at InhA active site (PDB ID 4TZK).

To study the interface approach of the synthesized compounds with the important amino acid residues at the dynamic site of ENR-ACP reductase, molecular docking protocol was performed for all the 28 *N’*-substituted-2-oxoethyl 4-(1*H*-pyrrol-1-yl) benzoate series into the dynamic site of InhA enzyme (Fig 1A and 1B). The outcomes of surflex docking study are portrayed in Table 1. Docking results showed interactions of approximately all the synthesized molecules in the active site of the enzyme (Fig 1A and B) similar to that of pyrrolidine carboxamide (Fig S1A and S1B in S2 File). The 4TZK-ligand displayed two H-bonding connections at the active site, the oxygen atom (---O-C) of pyrrolidine ring carbonyl group showed H-bonding interface with co-factor NAD^+^ and amino acid TYR158. We determined the generally distinguishing receptor-ligand connections of the molecule **4a** (pthalazine series **4a-g**) as projected by the Surflex-Dock binding approach, wherein it was institute that binding approach of hit molecules **4a** is parallel to that of the stated co-crystallized ligand 4TZK. As shown in Fig S2A and S2B in S2 File, oxygens of (-C = O-) pyridazinone carbonyl group present at the 3^rd^ and 6^th^ carbon of molecule **4a** showed H-bonding interface each with the co-factor NAD^+^ (2.61 Å), and the amino acid residue TYR158 (2.24 Å). The oxygen of (-C = O-NH) carbonyl group of the same molecule produced H-bonding communication with the co-factor NAD^+^ (1.83 Å) and TYR158 (1.68 Å). From the **5a-p** series, the molecule **5h** (Fig S3A and S3B in S2 File) formed 2 H-bonding interactions at the InhA active site. The oxygen atom of carbonyl group created two H-bonds, one with TYR158 (2.10 Å) amino acid and one more with co-factor NAD^+^ (1.82 Å). While, compound **6a** (Fig S4A and S4B in S2 File) from the third series showed two H-bonding interactions with the enzyme. The oxygen of -C = O group displayed two H-bonding connections, one with TYR158 amino acid (1.87 Å) while another with NAD^+^ co-factor (1.79 Å). and another oxygen of nitrogen showed interaction with amino acid TYR158 (2.01 Å). The nitrogen atom of nitro group formed H-bond with the same TYR158 amino acid residue (1.68 Å). The docking scores obtained from the Surflex docking study are specified in Table 1. The aminoacids [ALA22, ALA201, ALA191, ALA198, ALA213, ALA252, TRP259, LEU246, ILE25, ILE144, GLY212, GLY205, LEU108, LEU245, MET13, MET201, PHE91, PRO140, PRO151, PHE97, VAL91, VAL145, VAL189] and [ASP261, THR241, HIS265, TYR149, TYR158, TYR259] are important to understand the hydrophobic and hydrophilic interactions of **4a**(magenta), **5h**(blue) and **6a**(green) with InhA (Fig S5A and S5B in S2 File). All the hits exhibited a consensus score ranging from 8.08 to 3.68, representing the outline of all the forces of communication among the ligands and InhA enzyme. Protein and ligands connections includes electrostatic and van der Waals types that are found to fluctuate from -116.22 to -183.87. Helmholtz free energies of communications for protein-ligands molecular pairs ranged between -45.92 and -78.85, but its H-bonding, complex (ligand-protein) and internal (ligand-ligand) energies ranged from -187.68 to -296.60, whereas those values ranging from -23.79 to -42.92 indicated molecular scores with respect to H-bonding, lipophilic contact, and rotational entropy, along with the intercept terms. All these scores showed that the compounds selectively confine to InhA enzyme in contrast to the locus ligand 4TZK. The majority of hits may well bind to the substrate binding site of InhA. The H-bonding connection approach with amino acid TYR158 and co-factor NAD + is important for InhA enzyme inhibition activity. Majority of the synthesized compounds were found to show H-bonding exchanges with TYR158 and cofactor NAD+ similar to that of ligand 4TZK and therefore, can be considered as the enhanced ENR inhibitors. The binding mode of these compounds indicates that these molecules may have similar mechanism of action as identified for InhA inhibitors.

**Table 1:** Surflex dock scores (kcal/mol) of pyrrolyl benzohydrazide derivatives on PDB ID: 4TZK.

Comp.	C score^a^	Crash score^b^	Polar score^c^	D score^d^	PMF score^e^	G score^f^	Chem score^g^
**4TZK ligand**	8.735	-1.39	1.18	-168.117	-49.195	-285.296	-37.478
**4a**	6.28	-4.62	1.59	-183.876	-57.353	-296.207	-38.194
**4b**	3.68	-2.02	1.07	-153.355	-52.947	-206.045	-33.910
**4c**	5.23	-1.89	2.34	-155.212	-65.336	-223.078	-38.864
**4d**	6.24	-1.91	1.76	-111.173	-72.397	-211.892	-23.794
**4e**	5.58	-1.59	0.95	-132.276	-72.309	-243.999	-36.455
**4f**	5.88	-1.41	1.81	-114.177	-75.520	-207.450	-24.835
**4g**	4.83	-1.39	0.88	-125.921	-59.323	-226.893	-26.543
**5a**	6.56	-1.61	1.96	-131.091	-52.920	-223.240	-37.773
**5b**	5.62	-1.00	1.39	-110.065	-57.538	-187.684	-31.979
**5c**	6.54	-2.81	1.48	-144.172	-45.921	-282.933	-39.739
**5d**	6.21	-1.25	0.77	-127.620	-71.217	-234.664	-29.682
**5e**	7.19	-2.51	1.53	-145.828	-59.437	-282.096	-39.911
**5f**	5.58	-1.59	0.95	-132.276	-72.309	-243.999	-36.455
**5g**	7.23	-2.48	3.12	-174.554	-67.597	-287.451	-42.922
**5h**	7.12	-2.48	1.14	-147.209	-57.490	-262.270	-39.549
**5i**	6.84	-0.74	1.37	-135.381	-65.650	-222.897	-36.239
**5j**	7.60	-1.79	1.65	-139.602	-60.313	-264.029	-35.915
**5k**	5.71	-2.59	1.65	-140.591	-53.387	-263.589	-38.264
**5l**	6.62	-1.19	1.15	-143.742	-55.195	-263.875	-40.041
**5m**	6.84	-1.68	0.85	-170.921	-60.600	-280.502	-38.440
**5n**	4.61	-1.25	1.15	-116.221	-66.571	-200.106	-33.740
**5o**	7.11	-2.80	1.50	-139.386	-68.607	-280.196	-38.585
**5p**	7.77	-1.98	1.55	-138.330	-60.335	-255.501	-36.760
**6a**	8.08	-1.03	1.71	-134.813	-70.459	-260.486	-35.725
**6b**	6.72	-0.77	1.78	-139.006	-78.853	-260.120	-37.592
**6c**	7.40	-1.61	1.81	-145.344	-59.002	-249.994	-35.544
**6d**	6.67	-2.04	1.43	-139.569	-59.066	-255.628	-38.342
**6e**	5.62	-1.21	2.02	-116.387	-54.409	-220.114	-33.567

**Fig 2:**
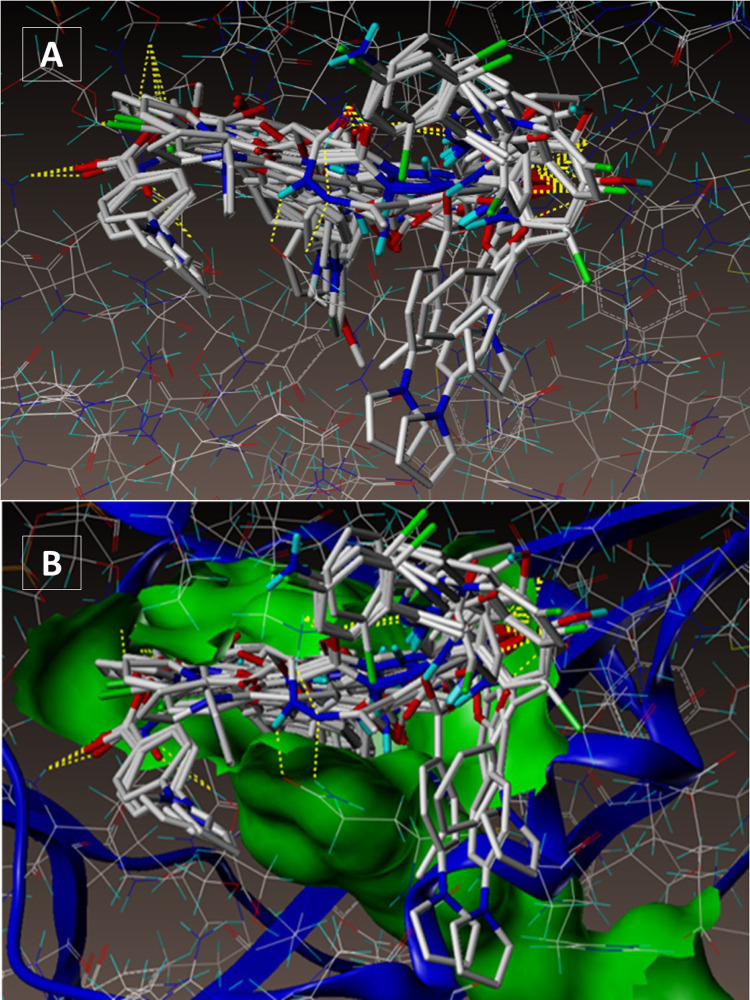
(A-B): Docked mode of all the 28 synthesized compounds at InhA active site (PDB ID1DF7).

All of the compounds had excellent docking scores (Fig 2A and 2B) against the dihydrofolate reductase forms of *Mycobacterium* TB, similar to that of ligand 1DF7 ligand (methotrexate) (Fig S6A and S6B in S2 File) according to a second docking investigation using PDB ID: 1DF7 (Table 2).

Compound **4a** forms three hydrogen bonding contacts at the enzyme’s active site, as depicted in Fig S7A and S7B in S2 File (PDB ID: 1DF7). ARG32’s hydrogen atom is involved in one hydrogen bond raised by the oxygen of the benzoate’s C = O group (-O---H-ARG32, 2.05 Å), while ARG60’s hydrogen atom is involved in another two interactions (-O----H- ARG60, 2.40 Å, 1.89 Å) by the -C = O group. As seen in Fig S8A and S8B in S2 File (PDB ID: 1DF7), compound **5h** creates three hydrogen bonds in the enzyme’s active site. These bonds are formed by the hydrogen atoms of ARG32 and ARG60 with the oxygen atom of the benzoate’s C = O group (-O---H-ARG32, 2.17 Å; O---H-ARG60, 2.04, 2.48 Å). Fig S7A and S7B in S2 File show the docked picture compound **6a** creates three hydrogen bonds in the enzyme’s active site. These bonds are formed by the hydrogen atoms of ARG632and ARG60 with the oxygen atom of the benzoate’s C = O group (-O---H-ARG32, 2.32 Å; O---H-ARG60, 1.88, 2.26 Å). Fig S8A and S8B in S2 File depict the hydrophobic and hydrophilic amino acids that are encircled by the two compounds **4a, 5h** and **6a**.

**Table 2:** Surflex dock scores (kcal/mol) of pyrrolyl benzohydrazide derivatives on PDB ID: 1DF7.

Comp.	C score^a^	Crash score^b^	Polar score^c^	D score^d^	PMF score^e^	G score^f^	Chem score^g^
**1DF7_ligand**	13.76	-1.32	8.92	-229.875	-138.104	-353.514	-38.494
**4a**	6.23	-0.76	2.24	-64.371	-61.949	-162.755	-24.989
**4b**	5.64	-0.72	2.50	-103.883	-41.344	-160.240	-27.941
**4c**	6.33	-1.93	2.19	-112.200	-64.967	-224.049	-31.913
**4d**	5.94	-0.90	0.80	-125.287	-62.937	-218.675	-26.688
**4e**	5.32	-0.83	1.45	-94.838	-64.550	-182.968	-20.911
**4f**	6.79	-0.57	2.23	-84.470	-44.297	-170.849	-21.618
**4g**	6.10	-0.28	2.30	-80.198	-52.538	-154.887	-21.328
**5a**	6.41	-1.40	2.89	-95.751	-53.054	-177.017	-25.508
**5b**	5.29	-0.86	1.10	-99.230	-35.264	-190.693	-28.997
**5c**	6.42	-1.44	2.86	-95.324	-50.422	-181.275	-25.613
**5d**	8.41	-3.12	6.31	-158.717	-68.381	-230.089	-46.326
**5e**	5.96	-1.43	3.28	-73.337	-32.472	-178.884	-26.344
**5f**	6.00	-0.87	0.52	-129.351	-63.792	-185.561	-30.340
**5g**	7.87	-1.47	3.88	-83.176	-82.037	-188.484	-33.224
**5h**	7.83	-1.62	2.09	-96.148	-70.385	-209.409	-31.600
**5i**	7.12	-1.58	1.11	-124.411	-50.620	-234.159	-30.316
**5j**	6.13	-0.81	0.84	-128.942	-62.215	-200.635	-27.318
**5k**	6.83	-0.85	226	-117.576	-53.462	-194.066	-34.385
**5l**	6.00	-0.58	0.40	-127.218	-61.016	-182.989	-27.746
**5m**	5.65	-1.06	3.38	-80.967	-59.910	-120.307	-29.581
**5n**	5.72	-1.13	1.22	-79.809	-56.148	-125.057	-28.349
**5o**	5.95	-0.94	3.33	-80.961	-55.162	-118.376	-27.959
**5p**	6.07	-0.61	0.37	-126.989	-60.382	-175.534	-26.563
**6a**	5.48	-1.32	2.28	-97.306	-64.277	-195.467	-28.282
**6b**	5.89	-1.00	3.39	-78.588	-62.488	-144.401	-29.559
**6c**	7.92	-1.46	3.83	-82.814	-72.527	-178.581	-32.097
**6d**	5.38	0.78	1.86	-103.164	-57.997	-164.863	-23.822
**6e**	5.96	-1.12	1.12	-100.406	-33.858	-215.533	-26.256

### 2.2. Anti-tubercular and Antibacterial activities

Antitubercular efficacy of all the newly produced molecules were established against *M. tuberculosis* H37Rv strain and their minimal inhibitory concentrations (MIC) values are compiled in Table 3 and these results are compared with the standard drugs isoniazid and streptomycin.

**Table 3:** *In vitro* evaluation of antitubercular and antibacterial activities. The values represent the mean ± standard error of the mean (SEM) obtained from three separate and independent measurements (n = 3).

Compound	*M. tuberculosis* H37Rv MIC values (μg/mL)	% InhA Inhibition at 50 µM	*S. aureus* *ATCC* 29213 (Gram +ve)	*E. coli* *ATCC* 25922 (Gram –ve)	IC_50_ (µM) MtDHFR
MIC (μg/mL)	MIC (μg/mL)	
**4a**	1.6	43	12.5	3.12	23
**4b**	1.6	29	6.25	0.8	25
**4c**	1.6	51	6.25	0.8	21
**4d**	1.6	NT	6.25	1.6	31
**4e**	1.6	NT	6.25	1.6	32
**4f**	1.6	23	3.12	0.8	35
**4g**	1.6	NT	3.12	0.8	56
**5a**	1.6	26	12.5	1.6	95
**5b**	0.8	22	1.6	0.4	23
**5c**	1.6	NT	6.25	1.6	100
**5d**	1.6	NT	6.25	1.6	121
**5e**	1.6	NT	12.5	3.12	118
**5f**	1.6	43	3.12	1.6	153
**5g**	1.6	34	6.25	1.6	132
**5h**	1.6	NT	12.5	3.12	124
**5i**	3.12	NT	6.25	1.6	129
**5j**	1.6	32	12.5	3.12	70
**5k**	1.6	32	12.5	3.12	69
**5l**	1.6	46	12.5	3.12	65
**5m**	3.12	NT	6.25	0.8	90
**5n**	3.12	NT	6.25	0.8	93
**5o**	3.12	NT	12.5	3.12	90
**5p**	3.12	NT	12.5	3.12	115
**6a**	1.6	13	12.5	1.6	132
**6b**	1.6	NT	3.12	1.6	130
**6c**	1.6	9	6.25	1.6	133
**6d**	0.8	18	3.12	1.6	62
**6e**	1.6	NT	6.25	1.6	69
**Pyrazinamide**	3.12	–	–	–	–
**Isoniazid**	1.6	> 99%	–	–	–
**Streptomycin**	0.8	–	2	–	–
**Ciprofloxacin**	–	–	2	2	–
**Triclosan**	–	> 99%	–	–	–
**Trimethoprim**	–	–	–	–	92

All the reported compounds have showed the good minimum inhibition value in the range of 1.6–3.12µg/mL. Among the compounds from all the three series, molecules **5b** and **6d** emerged as very effective compounds with a minimum inhibition value of 0.8µg/mL.

### 2.3. InhA inhibition assay

*In vitro* inhibition study of InhA enzyme from *M. tuberculosis* at 50 µM concentration was carried out for the chosen molecules and results are given in Table 3. All the molecules examined in the first series showed comparable results against InhA and showed inhibition in the range of 23% and 51% at 50 µM, among these the least value for **4b, 4f** and the maximum for **4c** were observed. Molecules (Schiff bases) from the second series, all the tested molecules emerged as moderate inhibitors in the range of 22–43% at 50 µM. Compound **6a, 6c** and **6d** from the third series showed moderate InhA inhibition (9–18% at 50 µM), while the other compounds **6b** and **6e** have no inhibition towards InhA. The IC_50_ values revealed InhA activities that are found to be moderate to good inhibitors at 50 µM concentrations.

### 2.4. *In vitro* Anti-bacterial growth inhibition study

**Scheme 1:**
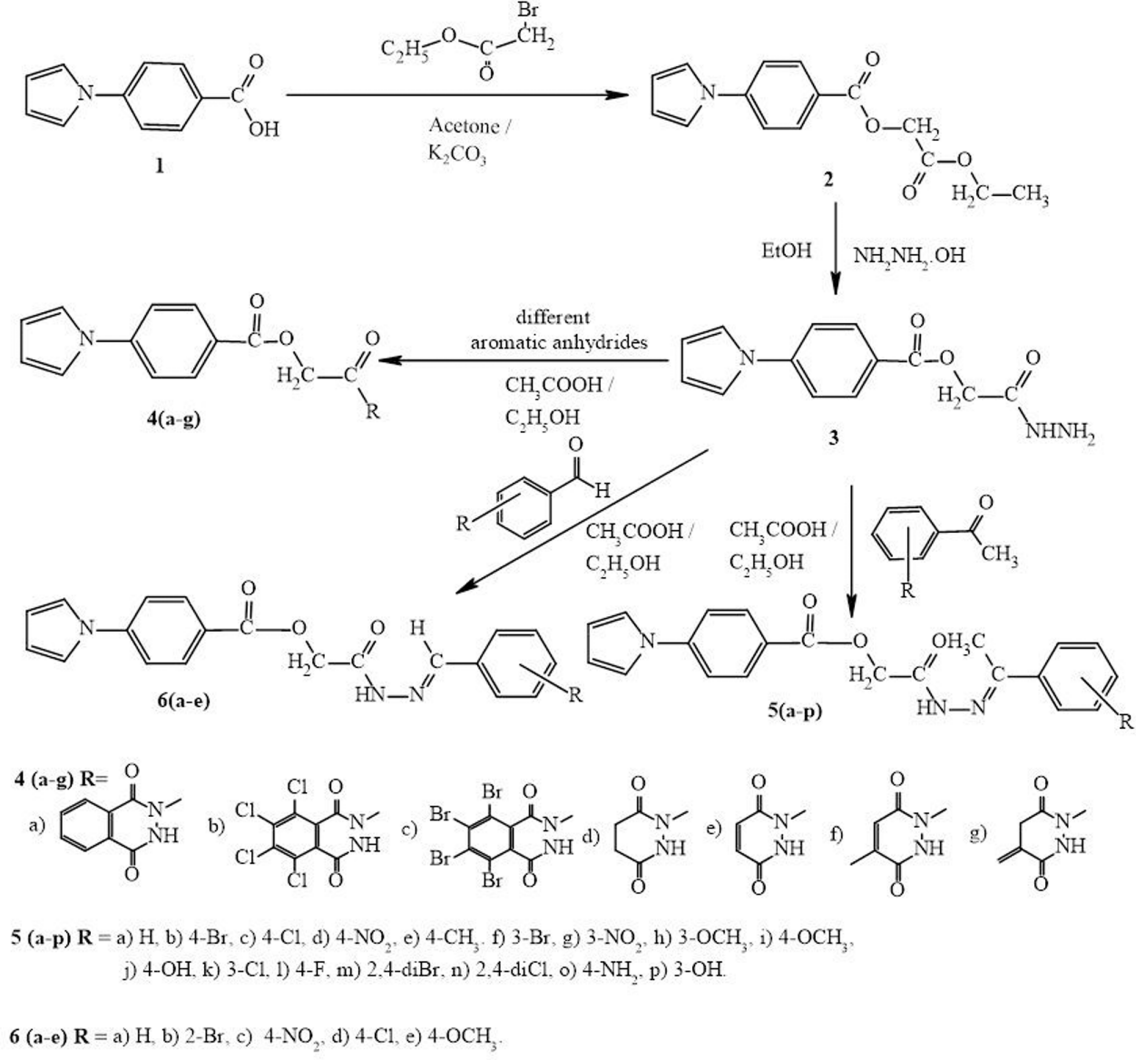
Synthetic route for the novel pyrrolyl-benzoate derivatives.

All twenty-eight molecules were evaluated for *in vitro* antimicrobial activities that were assessed against Gram +ve and Gram -ve strains. Antimicrobial data are tabulated in Table 3. All the tested molecules displayed moderate to good bacterial inhibition with the MIC values ranges from 0.4 to 12.5 µg/mL. In this case ciprofloxacin was used as a reference drug molecule. Compounds **4b, 4c, 4f** and **4g** from the first seriesrobustly controlled *E. coli* progression (MIC = 0.8 µg/mL) showing moderate inhibition against *S. aureus* growth (MIC = 3.12–6.25 µg/mL). Molecules **5b, 5m** and **5n** from the second series exhibited the highest potency against *E. coli* bacteria (Gram -ve) with the MIC value of 0.4–0.8 µg/mL. While, compounds **5a, 5c, 5d, 5f, 5g** and **5i** exhibited the excellent potency against *E. coli* bacteria (Gram -ve) with the MIC value of 1.6 µg/mL. Compounds **6(a-e)** from the third series, emerged as much potent molecules against *E. coli* with MIC value of 1.6 µg/mL. All the reported molecules from Scheme 1 showed moderate inhibition against *S. aureus* (MIC = 3.12–12.5 µg/mL) and excellent inhibition towards *E. coli* (MIC = 0.4–3.12 µg/mL) growth. These data exhibited that all the reported molecules have a better activity against *E. coli* and moderate activity against *S. aureas.*

### 2.5. MtDHFR inhibitory activity

By observing the fluorescence given off by MtDHFR substrates when stimulated at 340 nm, the *in vitro* MtDHFR inhibitory activity of our newly synthesized compounds was determined (Table 3). Since the product (NADP+) is not luminescent, the enzyme’s activity was evaluated by consuming its substrate. Trimethoprim, the assay’s positive control, had an IC_50_ value of 92 μM using this method, which was consistent with the findings from the literature (88 μM). Table 3 shows that the majority of the investigated compounds were much more effective against MtDHFR. Seven of them (**4a, 4b, 4c, 4d, 4e, 4f** and **5b**) had greater inhibition characteristics than trimethoprim while six have shown moderate inhibition (**4g, 5j, 5k, 5l, 6d** and **6e**).

### 2.6. MTT-based cytotoxicity studies

The cytotoxic effects of seventeen selected molecules were assessed against the A549 human lung adenocarcinoma cell line using the MTT assay. Cisplatin served as the reference drug for comparison. The IC_50_ values (concentration of the compound needed to inhibit cell viability by 50%) for the compounds are listed in Table 4. Lower IC_50_ values indicate higher cytotoxicity.

**Table 4:** *In vitro* MTT-based cytotoxicity activity of selected compounds against human lung cancer cell line A549 (IC_50_ in µg/mL).

Comp.	Human lung cancer cell line A549
	IC_50_ in µg/mL
**4a**	315 ± 0.3
**4b**	319 ± 0.4
**4c**	287 ± 0.7
**4f**	258 ± 0.1
**5a**	225 ± 0.3
**5b**	266 ± 0.3
**5f**	275 ± 0.4
**5g**	249 ± 0.9
**5j**	255 ± 0.3
**5k**	229 ± 0.4
**5l**	241 ± 0.6
**6a**	244 ± 0.8
**6c**	296 ± 0.3
**6d**	262 ± 0.7
**INH**	>450
**Cisplatin**	9.90

Most of the evaluated compounds exhibited moderate to low cytotoxicity against A549 cells, with IC_50_ values ranging from 225 to 319 µg/mL. Cisplatin, a known anticancer drug, showed a significantly higher cytotoxic effect with an IC_50_ value of 9.90 µg/mL, indicating its potent anticancer activity. The cytotoxicity of the compounds was also compared to Isoniazid (INH), a common anti-TB drug, which showed an IC_50_ value of over 450 µg/mL. All evaluated compounds demonstrated higher cytotoxicity compared to INH. Compounds such as 4b (IC_50_ = 319 µg/mL), 4a (IC_50_ = 315 µg/mL), and 6c (IC_50_ = 296 µg/mL) displayed relatively lower cytotoxicity among the evaluated molecules. The MTT assay results indicate that while the evaluated compounds show reasonable cytotoxicity against the A549 cell line, their cytotoxic effects are significantly lower compared to cisplatin. Further studies are needed to optimize the therapeutic potential of these compounds as anti-TB agents, balancing their cytotoxicity and therapeutic efficacy.

### 2.7. ADMET studies

The Swiss ADME web tool calculated the ADME properties of every synthesised compound, and every molecule complies with Lipinki’s rule of five. All compounds have good synthetic accessibility, moderate solubility, and high GI absorption, according to Swiss ADME tests. The BBB is only crossed by compounds **5a**, **5b, 5c, 5e, 5f, 5k, 5l, 5n, 6a, 6b** and **6d**. The substances had skin permeability that ranged from -5.64 to -7.93, which was moderate. Table S1 in S2 File presents the outcomes.

ProTox-II determined the toxicological summaries of all the chemicals, and Table S2 in S2 File shows the toxic effects. This data on toxicity demonstrated that none of the molecules shown any toxicity.

## 3. Experimental Section

### 3.1. Chemicals

Chemicals were procured from Spectrochem Pvt Ltd, Sigma Aldrich, and S. D. Fine Chem. Ltd. for the purpose of synthesizing the mentioned compounds. Both the recrystallization procedure and the distillation method were employed for the purification of various compounds and solvents.

### 3.2. Instruments

Shital digital melting point apparatus were used to record melting points and are uncorrected. Maximum absorbance wavelength recorded on RIGOL 3660, UV Visible spectrophotometer. FTIR spectra were recorded using Bruker α-T FTIR spectrophotometer using KBr pellet method. The ^1^H and ^13^C NMR spectra were documented using indicated solvents (Bruker AVANCE II) at 400 and 100/75 MHz, correspondingly; chemical shifts were expressed in parts per million (δ ppm) in relation to TMS. Acronyms used to define the peak outlines are: (s) singlet, (d) doublet, (t) triplet, (q) quartet and (m) multiplet. Mass spectra (MS) were recorded in a Schimadzu QP − 20105 GC−Mass spectrometer and WATERS Q − Tof Premier Mass spectrometer. Elemental analysis is studied using a TRUSPEC CHN rapid analyzer.

### 3.3. General procedure

#### 3.3.1 Synthesis of 2-ethoxy-2-oxoethyli4-(1*H*-pyrrol-1-yl) benzoate (2):.

A round bottom flask was charged with 4−(1*H*−pyrrol−1 − yl)benzoic acid **(1)** (1.0 eq), anhydrous K_2_CO_3_ (3.0 eq) and catalytic amount of KI; the mixture was stirred for 2 min in dry acetone (25mL) to which ethyl bromoacetate (1.2 eq.) was added and stirred for 48 h at 80ºC and monitoring the reaction by TLC. After cooling to ambient temperature, the mixture was filtered over a pad of celite and further rinsed with acetone (60 mL). Solvent was evaporated and the crude solid was recrystallized in diethyl ether to give the desired product [17].

White crystalline solid. (Yield 78%). M.p 100–102 °C; FTIR (KBr-cm^-^): 2988, 2933 (Ar-C = CH), 1758 (C = O), 17221 (C = O), 1223 (C-O-C).

1 H NMR (8 mg- CDCl_3_-*d*_*6*_, 400 MHz, T-18.85 ^o^C, *δ* ppm): 8.15–8.13 (2H, d, *J *= 6.76 Hz, bridging phenyl-C_8_, C_10_), 7.46–7.44 (2H, d, *J *= 6.88 Hz, bridging phenyl-C_7_, C_11_), 7.17–7.15 (2H, m, pyrrole-C_2_, C_5_), 6.38–6.37 (2H, m, pyrrole-C_3_, C_4_), 4.84 (2H, s, oxoethyl- CH_2_-C_14_), 4.29–4.23 (2H, m, ethoxy- CH_2_-C_17_), 1.32–1.28 (3H, 3, ethoxy-CH_3_-C_18_).

#### 3.3.2. Synthesis of 2-hydrazineyl-2-oxoethyl-4-(1*H*-pyrrol-1-yl) benzoate(3):.

2-ethoxy-2-oxoethyl-4-(1*H*-pyrrol-1-yl) benzoate (**2)** (3.47g, 15 mmol) was refluxed with hydrazine hydrate (10 mL) in absolute ethanol (10 mL for 6h). The reaction mixture was cooled and the mass obtained was recrystallized using ethanol [17].

Yellow crystalline solid. (Yield 76%). M.p 170–172 °C; FTIR (KBr-cm^-^): 3314 (NH_2_), 3263 (NH), 2917, 2850 (Ar-C = CH), 1704 (C = O), 1643 (C = O), 1281 (C-O-C).

1 H NMR (8 mg-DMSO-*d*_*6*_, 400 MHz, T-18.85 ^o^C, *δ* ppm): 9.80 (1H, s, NH), 7.93–7.91 (2H, d, *J *= 7.00 Hz, bridging phenyl-C_8_, C_10_), 7.60–7.58 (2H, d, *J *= 8.72 Hz, bridging phenyl-C_7_, C_11_), 7.37–7.36 (2H, m, pyrrole-C_2_, C_5_), 6.28–6.26 (2H, m, pyrrole-C_3_, C_4_), 5.05 (2H, s, oxoethyl- CH_2_-C_14_), 4.41 (2H, s, NH_2_).

Mass (ESI- *m/z*) = Found 259.19 (M^+^), Calcd.259.10.

#### 3.3.3. General procedure for the synthesis of 2-(1, 4-dioxo-3, 4-dihydrophthalazin-2 (1*H*)-yl)-2-oxoethyl-4-(1*H*-pyrrol-1-yl) benzoate (4a-g):.

Equimolar quantity of 2-hydrazineyl-2-oxoethyl-4-(1*H*-pyrrol-1-yl) benzoate (**3)** (0.01mol) and appropriate aromatic anhydrides (0.01mol) was refluxed for 6h. Completion of the reaction is monitored by TLC and then reaction mixture was cooled and 10% Na_2_CO_3_ solution was added and stirred for 5 min. Solution was filtered, dried, and purified by column chromatography using ethylacetate:petroleum ether (6:4) mobile phase [17].

#### 3.3.4. General procedure for the synthesis of 2-oxo-2-(2-(1-phenylethylidene) hydrazineyl) ethyl-4-(1*H*-pyrrol-1-yl) benzoates (5a-p):.

Equimolar quantity of 2-hydrazineyl-2-oxoethyl-4-(1*H*-pyrrol-1-yl) benzoate (**3)** and appropriate aromatic ketones were refluxed in alcohol for 4–6 h in the presence of few drops of glacial acetic acid. The solvent was removed under reduced pressure and the residue was purified by column chromatography on silica gel with ethyl acetate/peteroleum ether (6:4) as eluent to afford phenylethylidene-hydrazineyl-pyrrolylibenzoates **(5a-p)** in good yields with high purity [17].

#### 3.3.5. General procedure for the synthesis of 2-(2-benzylidenehydrazineyl)-2-oxoethyl-4-(1*H*-pyrrol-1-yl) benzoates (6a-e):.

An equimolar quantity of 2-hydrazineyl-2-oxoethyl-4-(1*H*-pyrrol-1-yl) benzoate (**3)** and appropriate aromatic aldehydes were refluxed in alcohol for 8–12 h in the presence of few drops of glacial acetic acid. The solvent was removed under reduced pressure. The residue was purified by column chromatography on silica gel with ethyl acetate/petroleum ether (6:4) as eluent to afford hydrazones **(6a-e)** in good yields with high purity [17].

#### 3.3.6. Synthesis of 2-(1,4-dioxo-3,4-dihydrophthalazin-2(1H)-yl)-2-oxoethyl 4-(1H-pyrrol-1-yl)benzoate (4a):.

Brown crystalline solid. (Yield 55%). M.p 192–188 °C. FTIR (KBr-cm^-^): 3377 (NH), 2921, 2852 (Ar-C = CH), 1791 (C = O), 1722 (C = O), 1685 (C = O), 1262 (C-O-C).

^1^H NMR (8 mg-CDCl_3_-*d*_*6*_, 400 MHz, T-18.85 ^o^C, *δ* ppm): 11.31 (1H, s, NH), 8.10–8.08 (2H,d, *J *= 8.76 Hz, bridging phenyl-C_20_, C_24_), 8.00–7.93 (4H, m, dihydrophthalazine-C_7,_ C_8,_ C_9_ &C_10_), 7.77–7.75 (2H,d, *J *= 8.76 Hz, bridging phenyl-C_21_, C_23_), 7.47–7.46 (2H, m, pyrrole-C_26_, C_29_), 6.33–6.32 (2H, m, pyrrole-C_27_, C_28_), 5.02 (2H, s, oxoethyl-CH_2_-C_15_).

^13^C NMR (10 mg- CDCl_3_-*d*_*6*_, 100 MHz, T-18.85 ^o^C, *δ* ppm): 165.26, 164.51, 162.21, 157.28, 142.93, 135.10, 131.56, 129.56, 129.53, 126.86, 123.68, 118.83, 111.29, 78.77.

Mass (ESI- *m/z*) = Found 389.09 (M^+^), Calcd.389.10.

CHN Anal.For C_21_H_15_N_3_O_5_: Calcd.C, 64.78; H, 3.88; N, 10.79; O, 20.54; Found.C, 64.78; H, 3.88; N, 10.79; O, 20.54.

#### 3.3.7. Synthesis of 2-oxo-2-(5,6,7,8-tetrachloro-1,4-dioxo-3,4-dihydrophthalazin-2(1H)-yl)ethyl 4-(1H-pyrrol-1-yl)benzoate (4b):.

Brown crystalline solid. (Yield 56%). M.p 194–195 °C. FTIR (KBr-cm^-^): 3396 (NH), 2921, 2854 (Ar-C = CH), 1792 (C = O), 1733 (C = O), 1697 (C = O), 1260 (C-O-C).

^1^H NMR (8 mg-CDCl_3_-*d*_*6*_, 400 MHz, T-18.85 ^o^C, *δ* ppm): 11.55 (1H, s, NH), 8.09–8.06 (2H,d, *J *= 2.19 Hz, bridging phenyl-C_24_, C_28_), 7.84–7.82 (2H,d, *J *= 8.80 Hz, bridging phenyl-C_25_, C_27_), 7.56–7.55 (2H, m, pyrrole-C_30_, C_33_), 6.34–6.33 (2H, m, pyrrole-C_31_, C_32_), 5.03 (2H, s, oxoethyl-CH_2_-C_19_).

^13^C NMR (10 mg- CDCl_3_-*d*_*6*_, 100 MHz, T-18.85 ^o^C, *δ* ppm): 166.18, 164.15, 161.40, 152.29, 143.03, 139.33, 135.10, 129.68, 129.00, 126.43, 126.14, 118.57, 111.45, 77.67.

Mass (ESI- *m/z*) = Found 526.89 (M^+^), 528.97 (M^+^+2), Calcd.526.94.

CHN Anal. For C_21_H_11_Cl_4_N_3_O_5_: Calcd.C, 35.78; H, 1.57; Br, 45.34; N, 5.96; O, 11.35; Found.C, 47.85; H, 2.10; Cl, 26.90; N, 7.97; O, 15.18.

#### 3.3.8. Synthesis of 2-oxo-2-(5,6,7,8-tetrabromo-1,4-dioxo-3,4-dihydrophthalazin-2(1H)-yl)ethyl 4-(1H-pyrrol-1-yl)benzoate (4c):.

Brown crystalline solid. (Yield 62%). M.p 204–206 °C. FTIR (KBr-cm^-^): 3229 (NH), 2922, 2853 (Ar-C = CH), 1739 (C = O), 1677 (C = O), 1607 (C = O), 1276 (C-O-C).

^1^H NMR (8 mg-CDCl_3_-*d*_*6*_, 400 MHz, T-18.85 ^o^C, *δ* ppm): 11.46 (1H, s, NH), 8.06–8.04 (2H,d, *J *= 2.19 Hz, bridging phenyl-C_24_, C_28_), 7.82–7.80 (2H,d, *J *= 8.80 Hz, bridging phenyl-C_25_, C_27_), 7.54–7.53 (2H, m, pyrrole-C_30_, C_33_), 6.33–6.32 (2H, m, pyrrole-C_31_, C_32_), 5.02 (2H, s, oxoethyl-CH_2_-C_19_).

^13^C NMR (10 mg- CDCl_3_-*d*_*6*_, 100 MHz, T-18.85 ^o^C, *δ* ppm): 165.16, 164.17, 162.02, 153.09, 142.05, 139.13, 134.80, 129.88, 129.07, 126.53, 126.08, 118.77, 111.55, 76.97.

Mass (ESI- *m/z*) = Found 704.75 (M^+^), 706.82 (M^+^+2), Calcd.704.74.

CHN Anal. For C_21_H_11_Br_4_N_3_O_5_: Calcd.C, 35.78; H, 1.57; Br, 45.34; N, 5.96; O, 11.35; Found.C, 35.78; H, 1.57; Br, 45.34; N, 5.96; O, 11.35.

#### 3.3.9. Synthesis of 2-(3,6-dioxotetrahydropyridazin-1(2H)-yl)-2-oxoethyl 4-(1H-pyrrol-1-yl)benzoate (4d):.

Brown crystalline solid. (Yield 61%). M.p > 250 °C. FTIR (KBr-cm^-^): 3204 (NH), 2923, 2856 (Ar-C = CH), 1690 (C = O), 1642 (C = O), 1601 (C = O), 1299 (C-O-C).

^1^H NMR (8 mg-CDCl_3_-*d*_*6*_, 400 MHz, T-18.85 ^o^C, *δ* ppm): 11.21 (1H, s, NH), 8.12–8.10 (2H,d, *J *= 7.76 Hz, bridging phenyl-C_16_, C_20_), 7.93–7.91 (2H,d, *J *= 7.26 Hz, bridging phenyl-C_17_, C_19_), 7.39–7.37 (2H, m, pyrrole-C_22_, C_25_), 6.37–6.35 (2H, m, pyrrole-C_23_, C_24_), 5.03 (2H, s, oxoethyl-CH_2_-C_11_), 2.27 (4H, s, dioxotetrahydropyridazine-CH_2_-C_4_, C_5_).

^13^C NMR (10 mg- CDCl_3_-*d*_*6*_, 100 MHz, T-18.85 ^o^C, *δ* ppm): 166.25, 164.43, 161.12, 153.33, 142.88, 135.21, 131.48, 129.55, 126.88, 123.63, 118.85, 111.22, 75.67.

Mass (ESI- *m/z*) = Found 341.07 (M^+^), Calcd.341.10.

CHN Anal. For C_17_H_15_N_3_O_5_: Calcd.C, 59.82; H, 4.43; N, 12.31; O, 23.44; Found.C, 59.82; H, 4.43; N, 12.31; O, 23.44.

#### 3.3.10. Synthesis of 2-(3,6-dioxo-2,3-dihydropyridazin-1(6H)-yl)-2-oxoethyl 4-(1H-pyrrol-1-yl)benzoate (4e):.

Brown crystalline solid. (Yield 58%). M.p 210–212 °C. FTIR (KBr-cm^-^): 3231 (NH), 2922, 2853 (Ar-C = CH), 1739 (C = O), 1676 (C = O), 1606 (C = O), 1275 (C-O-C).

^1^H NMR (8 mg-CDCl_3_-*d*_*6*_, 400 MHz, T-18.85 ^o^C, *δ* ppm): 10.27 (1H, s, NH), 7.91–7.89 (2H,d, *J *= 9.26 Hz, bridging phenyl-C_16_, C_20_), 7.74–7.72 (1H, m, dioxodihydropyridazine-CH-C_5_),7.67–7.65 (2H,d, *J *= 8.80 Hz, bridging phenyl-C_17_, C_19_), 7.49–7.48 (1H, m, dioxodihydropyridazine-CH-C_4_),7.46–7.45 (2H, m, pyrrole-C_22_, C_25_), 6.30–6.27 (2H, m, pyrrole-C_23_, C_24_), 5.10 (2H, s, oxoethyl-CH_2_-C_11_).

^13^C NMR (10 mg- CDCl_3_-*d*_*6*_, 100 MHz, T-18.85 ^o^C, *δ* ppm): 168.64, 165.36, 161.61, 156.55, 151.50, 144.87, 142.08, 130.72, 130.44, 129.45, 128.77, 127.69, 118.88, 115.13, 111.15, 75.22.

Mass (ESI- *m/z*) = Found 339.10 (M^+^), Calcd.339.09.

CHN Anal. For C_17_H_13_N_3_O_5_: Calcd.C, 60.18; H, 3.86; N, 12.38; O, 23.58; Found. C, 60.18; H, 3.86; N, 12.38; O, 23.58.

#### 3.3.11. Synthesis of 2-(4-methyl-3,6-dioxo-2,3-dihydropyridazin-1(6H)-yl)-2-oxoethyl 4-(1H-pyrrol-1-yl)benzoate (4f):.

Brown crystalline solid. (Yield 52%). M.p 196–198 °C. FTIR (KBr-cm^-^): 3496 (NH), 2928, 2851 (Ar-C = CH), 1735 (C = O), 1653 (C = O), 1607 (C = O), 1289 (C-O-C).

^1^H NMR (8 mg-CDCl_3_-*d*_*6*_, 400 MHz, T-18.85 ^o^C, *δ* ppm): 11.03 (1H, s, NH), 8.00–7.98 (2H,d, *J *= 8.80 Hz, bridging phenyl-C_17_, C_21_), 7.79–7.77 (2H,d, *J *= 8.80 Hz, bridging phenyl-C_18_, C_20_), 7.53–7.51 (2H, m, pyrrole-C_26_, C_23_), 6.86 (1H, s, dioxodihydropyridazine-CH-C_4_),6.32–6.31 (2H, m, pyrrole-C_24_, C_25_), 5.11 (2H, s, oxoethyl-CH_2_-C_12_), 2.09 (3H, s, dioxodihydropyridazine-CH_3_-C_5_).

^13^C NMR (10 mg- CDCl_3_-*d*_*6*_, 100 MHz, T-18.85 ^o^C, *δ* ppm): 168.44, 164.36, 162.61, 156.48, 151.40, 144.67, 141.88, 130.62, 129.35, 128.75, 127.99, 118.28, 111.05, 76.64, 31.22.

Mass (ESI- *m/z*) = Found 353.08 (M^+^), Calcd.353.10.

CHN Anal. For C_18_H_15_N_3_O_5_: Calcd.C, 61.19; H, 4.28; N, 11.89; O, 22.64; Found. C, 61.19; H, 4.28; N, 11.89; O, 22.64.

#### 3.3.12. Synthesis of 2-(4-methylene-3,6-dioxotetrahydropyridazin-1(2H)-yl)-2-oxoethyl 4-(1H-pyrrol-1-yl)benzoate (4g):.

Brown crystalline solid. (Yield 58%). M.p 212–214 °C. FTIR (KBr-cm^-^): 3401 (NH), 2924, 2854 (Ar-C = CH), 1787 (C = O), 1729 (C = O), 1644 (C = O), 1290 (C-O-C).

^1^H NMR (8 mg-CDCl_3_-*d*_*6*_, 400 MHz, T-18.85 ^o^C, *δ* ppm): 10.31 (1H, s, NH), 7.96–7.94 (2H,d, *J *= 8.80 Hz, bridging phenyl-C_17_, C_21_), 7.71–7.69 (2H,d, *J *= 8.80 Hz, bridging phenyl-C_18_, C_20_), 7.50–7.48 (2H, m, pyrrole-C_26_, C_23_), 6.29 (1H, m, dioxodihydropyridazine-CH_2_-C_10_),6.30–6.27 (2H, m, pyrrole-C_24_, C_25_), 5.94 (1H, s, dioxodihydropyridazine-CH_2_-C_10_),5.48 (2H, s, oxoethyl-CH_2_-C_12_), 1.91 (2H, s, dioxodihydropyridazine-CH_2_-C_4_).

^3^C NMR (10 mg- CDCl_3_-*d*_*6*_, 100 MHz, T-18.85 ^o^C, *δ* ppm): 168.34, 164.35, 162.60, 158.84, 156.38, 144.88, 141.90, 130.61, 129.07, 127.78, 118.92, 111.03, 76.44, 31.41.

Mass (ESI- *m/z*) = Found 353.07 (M^+^), Calcd.353.10.

CHN Anal. For C_18_H_15_N_3_O_5_: Calcd.C, 61.19; H, 4.28; N, 11.89; O, 22.64; Found. C, 61.19; H, 4.28; N, 11.89; O, 22.64.

#### 3.3.13. Synthesis of 2-oxo-2-(2-(1-phenylethylidene)hydrazinyl)ethyl 4-(1H-pyrrol-1-yl)benzoate (5a):.

Yellow crystalline solid. (Yield 68%). M.p 168-169^o^C. FTIR (KBr-cm^-^): 3327 (NH), 2922, 28534 (Ar-C = CH), 1712 (C = O), 1664 (C = O), 1271 (C-O-C).

^1^H NMR (8 mg-CDCl_3_-*d*_*6*_, 400 MHz, T-18.85 ^o^C, *δ* ppm): 10.71 (1H, s, NH), 7.83–7.81 (2H,d, *J *= 8.08 Hz, bridging phenyl-C_8_, C_10_), 7.77–7.75 (2H,d, *J *= 7.72 Hz, bridging phenyl-C_7_, C_11_), 7.53–7.44 (5H,m, phenyl-C_23_, C_24,_ C_25,_ C_26_ andC_27_), 7.43–7.42 (2H, m, pyrrole-C_2_, C_5_), 6.33–6.32 (2H, m, pyrrole-C_3_, C_4_), 5.56 (2H, s, oxoethyl-CH_2_-C_14_), 2.21 (3H, s, phenylethylidene-CH_3_-C_21_).

^13^C NMR (10 mg- CDCl_3_-*d*_*6*_, 100 MHz, T-18.85 ^o^C, *δ* ppm): 174.36, 168.31, 146.49, 136.91, 136.83, 134.02, 133.67, 128.38, 128.33, 128.06, 127.17, 118.99, 111.14, 61.03, 18.83.

Mass (ESI- *m/z*) = Found 361.11 (M^+^), Calcd.361.14.

CHN Anal. For C_21_H_19_N_3_O_3_: Calcd.C, 69.79; H, 5.30; N, 11.63; O, 13.28; Found. C, 69.79; H, 5.30; N,11.63; O, 13.28.

#### 3.3.14. Synthesis of 2-(2-(1-(4-bromophenyl) ethylidene) hydrazineyl)-2-oxoethyl-4-(1*H*-pyrrol-1-yl) benzoate (5b):.

Yellow crystalline solid. (Yield 72%). M.p 150-152^o^C. FTIR (KBr-cm^-^): 3431 (NH), 2921, 2853 (Ar-C = CH), 1660 (C = O), 1610 (C = O), 1276 (C-O-C).

^1^H NMR (8 mg-CDCl_3_-*d*_*6*_, 400 MHz, T-18.85 ^o^C, *δ* ppm): 10.84 (1H, s, NH), 7.92–7.91 (2H,d, *J *= 7.20 Hz, bridging phenyl-C_8_, C_10_), 7.75–7.73 (2H,d, *J *= 6.40 Hz, bridging phenyl-C_7_, C_11_), 7.69–7.67 (2H,d, *J *= 6.40 Hz, phenyl-C_23_, C_27_), 7.48–7.47 (2H,m, phenyl-C_24_, C_26_), 7.52–7.51 (2H, m, pyrrole-C_2_, C_5_), 6.30–6.29 (2H, m, pyrrole-C_3_, C_4_), 4.80 (2H, s, oxoethyl-CH_2_-C_14_), 2.37 (3H, s, phenylethylidene-CH_3_-C_21_).

^13^C NMR (10 mg- CDCl_3_-*d*_*6*_, 100 MHz, T-18.85 ^o^C, *δ* ppm): 167.97, 164.98, 144.35, 141.62, 137.22, 135.27, 131.24, 129.50, 128.43, 128.31, 126.82, 118.93, 111.07, 62.03, 17.82.

Mass (ESI- *m/z*) = Found 439.18 (M^+^), 441.18 (M^+^+ 2), Calcd.439.05.

CHN Anal. For C_21_H_18_BrN_3_O_3_: Calcd.C, 57.29; H, 4.12; Br, 18.15; N, 9.54; O, 10.90; Found. C, 57.29; H, 4.12; Br, 18.15; N, 9.54; O, 10.90.

#### 3.3.15. Synthesis of 2-(2-(1-(4-chlorophenyl)ethylidene)hydrazinyl)-2-oxoethyl 4-(1H-pyrrol-1-yl)benzoate (5c):.

Yellow crystalline solid. (Yield 62%). M.p 176–178 °C. FTIR (KBr-cm^-^): 3328 (NH), 2923, 2854 (Ar-C = CH), 1708 (C = O), 1644 (C = O), 1272 (C-O-C).

^1^H NMR (8 mg-CDCl_3_-*d*_*6*_, 400 MHz, T-18.85 ^o^C, *δ* ppm): 10.83 (1H, s, NH), 7.93–7.92 (2H,d, *J *= 7.00 Hz, bridging phenyl-C_8_, C_10_), 7.82–7.81 (2H,d, *J *= 8.00 Hz, bridging phenyl-C_7_, C_11_), 7.74–7.73 (2H,d, *J *= 6.00 Hz, phenyl-C_23_, C_27_), 7.53–7.52 (2H,d, *J *= 4.50 Hz, phenyl-C_24_, C_26_), 7.50–7.48 (2H, m, pyrrole-C_2_, C_5_), 6.33–6.32 (2H, m, pyrrole-C_3_, C_4_), 4.66 (2H, s, oxoethyl-CH_2_-C_14_), 2.29 (3H, s, phenylethylidene-CH_3_-C_21_).

^13^C NMR (10 mg- CDCl_3_-*d*_*6*_, 100 MHz, T-18.85 ^o^C, *δ* ppm): 168.78, 167.39, 157.40, 147.01, 139.56, 137.34, 135.08, 134.51, 130.14, 128.94, 124.64, 119.49, 111.62, 71.61, 22.56.

Mass (ESI- *m/z*) = Found 395.17 (M^+^), 397.70 (M^+^+2). Calcd.395.10.

CHN Anal. For C_21_H_18_ClN_3_O_3_: Calcd.C, 63.72; H, 4.58; Cl, 8.96; N, 10.62; O, 12.13; Found. C, 63.72; H, 4.58; Cl, 8.96; N, 10.62; O, 12.13.

#### 3.3.16. Synthesis of 2-(2-(1-(4-nitrophenyl) ethylidene) hydrazineyl) -2-oxoethyl-4-(1*H*-pyrrol-1-yl) benzoate (5d):.

Yellow crystalline solid. (Yield 65%). M.p 188–190 °C. FTIR (KBr-cm^-^): 3268 (NH), 2923, 2853 (Ar-C = CH), 1720 (C = O), 1657 (C = O), 1271 (C-O-C).

^1^H NMR (8 mg-CDCl_3_-*d*_*6*_, 400 MHz, T-18.85 ^o^C, *δ* ppm): 10.99 (1H, s, NH), 8.32–8.31 (2H,d, *J *= 7.00 Hz, phenyl-C_24_, C_26_), 8.29–8.27 (2H,d, *J *= 8.50 Hz, bridging phenyl-C_8_, C_10_), 8.18–8.17 (2H,d, *J *= 5.00 Hz, phenyl-C_23_, C_27_), 7.74–7.73 (2H,d, *J *= 7.00 Hz, bridging phenyl-C_7_, C_11_), 7.52–7.51 (2H, m, pyrrole-C_2_, C_5_), 6.33–6.32 (2H, m, pyrrole-C_3_, C_4_), 4.66 (2H, s, oxoethyl-CH_2_-C_14_), 2.32 (3H, s, phenylethylidene-CH_3_-C_21_).

^13^C NMR (10 mg- CDCl_3_-*d*_*6*_, 100 MHz, T-18.85 ^o^C, *δ* ppm): 169.02, 166.89, 156.57, 148.57, 144.76, 143.79, 135.52, 134.36, 130.49, 128.34, 124.10, 119.51, 111.66, 71.61, 15.53.

Mass (ESI- *m/z*) = Found 406.11 (M^+^), Calcd.406.13.

CHN Anal. For C_21_H_18_N_4_O_5_: Calcd.C, 62.07; H, 4.46; N, 13.79; O, 19.68; Found. C, 62.07; H, 4.46; N, 13.79; O, 19.68.

#### 3.3.17. Synthesis of 2-oxo-2-(2-(1-(p-tolyl)ethylidene)hydrazinyl)ethyl 4-(1H-pyrrol-1-yl)benzoate (5e):.

Yellow crystalline solid. (Yield 66%). M.p 178–180 °C. FTIR (KBr-cm^-^): 3325 (NH), 2923, 2854 (Ar-C = CH), 1700 (C = O), 1664 (C = O), 1274 (C-O-C).

^1^H NMR (8 mg-CDCl_3_-*d*_*6*_, 400 MHz, T-18.85 ^o^C, *δ* ppm): 10.59 (1H, s, NH), 7.81–7.80 (2H,d, *J *= 8.50 Hz, bridging phenyl-C_8_, C_10_), 7.64–7.62 (2H,d, *J *= 7.50 Hz, bridging phenyl-C_7_, C_11_), 7.50–7.48 (2H,d, *J *= 6.20 Hz, phenyl-C_23_, C_27_), 7.27–7.25 (2H,d, *J *= 8.00 Hz, phenyl-C_24_, C_26_), 7.23–7.19 (2H, m, pyrrole-C_2_, C_5_), 6.33–6.32 (2H, m, pyrrole-C_3_, C_4_), 4.42 (2H, s, oxoethyl-CH_2_-C_14_), 2.37 (3H, s, phenyl-CH_3_-C_28_), 2.26 (3H, s, phenylethylidene-CH_3_-C_21_).

^13^C NMR (10 mg- CDCl_3_-*d*_*6*_, 100 MHz, T-18.85 ^o^C, *δ* ppm): 168.78, 167.39, 157.91, 152.90, 148.25, 139.56, 135.76, 132.20, 129.72, 126.88, 124.64, 119.48, 111.60, 71.61, 21.36.

Mass (ESI- *m/z*) = Found 375.18 (M^+^), Calcd.375.16.

CHN Anal. For C_22_H_21_N_3_O_3_: Calcd.C, 70.38; H, 5.64; N, 11.19; O, 12.78; Found. C, 70.38; H, 5.64; N, 11.19; O, 12.78.

#### 3.3.18. Synthesis of 2-(2-(1-(3-bromophenyl)ethylidene)hydrazinyl)-2-oxoethyl 4-(1H-pyrrol-1-yl)benzoate (5f):.

Yellow crystalline solid. (Yield 67%). M.p 180–182 °C. FTIR (KBr-cm^-^): 3433 (NH), 2920, 2853 (Ar-C = CH), 1657 (C = O), 1607 (C = O), 1279 (C-O-C).

^1^H NMR (8 mg-CDCl_3_-*d*_*6*_, 400 MHz, T-18.85 ^o^C, *δ* ppm): 10.85 (1H, s, NH), 8.24–8.22 (2H,d, *J *= 8.50 Hz, bridging phenyl-C_8_, C_10_), 7.96–7.82 (4H,M, phenyl- C_23_, C_25,_ C_26_, C_27_), 7.74–7.72 (2H,d, *J *= 8.50 Hz, bridging phenyl-C_7_, C_11_), 7.50 (2H, m, pyrrole-C_2_, C_5_), 6.31–6.28 (2H, m, pyrrole-C_3_, C_4_), 4.51 (2H, s, oxoethyl-CH_2_-C_14_), 2.37 (3H, s, phenylethylidene-CH_3_-C_21_).

^13^C NMR (10 mg- CDCl_3_-*d*_*6*_, 100 MHz, T-18.85 ^o^C, *δ* ppm): 169.29, 167.25, 158.19, 141.23, 135.55, 133.19, 129.93, 128.29, 126.98, 125.12, 121.73, 119.56, 110.45, 66.46, 16.46.

Mass (ESI- *m/z*) = Found 439.08 (M^+^), 441.02 (M^+^+2), Calcd.439.05.

CHN Anal. For C_21_H_18_BrN_3_O_3_: Calcd.C, 57.29; H, 4.12; N, 9.54; Found. C, 57.26; H, 4.11; N, 9.52.

#### 3.3.19. Synthesis of 2-(2-(1-(3-nitrophenyl)ethylidene)hydrazinyl)-2-oxoethyl 4-(1H-pyrrol-1-yl)benzoate (5g):.

Yellow crystalline solid. (Yield 74%). M.p 190–192 °C. FTIR (KBr-cm^-^): 3430 (NH), 2921, 2853 (Ar-C = CH), 1658 (C = O), 1606(C = O), 1271 (C-O-C).

^1^H NMR (8 mg-CDCl_3_-*d*_*6*_, 400 MHz, T-18.85 ^o^C, *δ* ppm): 10.96 (1H, s, NH),8.69 (1H, s, phenyl-C_23_)_,_ 8.26–8.25 (2H,d, *J *= 6 Hz, bridging phenyl-C_8_, C_10_), 7.98 (2H,m, phenyl-C_26_, C_27_), 7.75–7.73 (3H,d, *J *= 8 Hz, bridging phenyl-C_7_, C_11,_ phenyl-C_25_), 7.51–7.50 (2H, m, pyrrole-C_2_, C_5_), 6.32–6.28 (2H, m, pyrrole-C_3_, C_4_), 4.48 (2H, s, oxoethyl-CH_2_-C_14_), 2.45 (3H, s, phenylethylidene-CH_3_-C_21_).

^13^C NMR (10 mg- CDCl_3_-*d*_*6*_, 100 MHz, T-18.85 ^o^C, *δ* ppm): 165.66, 165.10, 159.40, 158.11, 149.99, 140.40, 130.59, 129.16, 128.04, 127.99, 126.54, 123.78, 120.93, 107.15, 61.38, 14.57.

Mass (ESI- *m/z*) = Found 406.14 (M^+^), Calcd.406.13.

CHN Anal. For C_21_H_18_N_4_O_5_: Calcd.C, 62.07; H, 4.46; N, 13.79; Found. C, 62.05; H, 4.46; N, 13.77.

#### 3.3.20. Synthesis of 2-(2-(1-(3-methoxyphenyl)ethylidene)hydrazinyl)-2-oxoethyl 4-(1H-pyrrol-1-yl)benzoate (5h):.

Yellow crystalline solid. (Yield 76%). M.p 196–198 °C. FTIR (KBr-cm^-^): 3324 (NH), 2919, 2833 (Ar-C = CH), 1664 (C = O), 1605 (C = O), 1270 (C-O-C).

^1^H NMR (8 mg-CDCl_3_-*d*_*6*_, 400 MHz, T-18.85 ^o^C, *δ* ppm): 10.92 (1H, s, NH), 8.10–7.99 (2H,d, *J *= 5.55 Hz, bridging phenyl-C_8_, C_10_), 7.49–7.47 (2H,d, *J *= 8.50 Hz, bridging phenyl-C_7_, C_11_), 7.30–7.26 (3H,m, phenyl-C_25_, C_26,_ C_27_), 7.17 (2H,s, phenyl-C_23_), 7.16–7.14 (2H, m, pyrrole-C_2_, C_5_), 6.40–6.39 (2H, m, pyrrole-C_3_, C_4_), 4.63 (2H, s, oxoethyl-CH_2_-C_14_), 3.87 (3H, s, phenyl-OCH_3_-C_23_), 2.33 (3H, s, phenylethylidene-CH_3_-C_21_).

^13^C NMR (10 mg- CDCl_3_-*d*_*6*_, 100 MHz, T-18.85 ^o^C, *δ* ppm): 171.09, 168.00, 159.90, 144.92, 139.29, 132.53, 131.29, 129.68, 128.88, 119.27, 111.85, 55.67, 55.53, 25.25.

Mass (ESI-*m/z*) = Found 391.13 (M^+^). Calcd.391.15.

CHN Anal. For C_22_H_21_N_3_O_4_: Calcd.C, 67.51; H, 5.41; N, 10.74; Found. C, 67.50; H, 5.38; N, 10.71.

#### 3.3.21. Synthesis of 2-(2-(1-(4-methoxyphenyl)ethylidene)hydrazinyl)-2-oxoethyl 4-(1H-pyrrol-1-yl)benzoate (5i):.

Yellow crystalline solid. (Yield 61%). M.p 196–198 °C. FTIR (KBr-cm^-^): 3333 (NH), 3012, 2929 (Ar-C = CH), 1664(C = O), 1604 (C = O), 1254 (C-O-C).

^1^H NMR (8 mg-CDCl_3_-*d*_*6*_, 400 MHz, T-18.85 ^o^C, *δ* ppm): 10.52 (1H, s, NH), 8.06–7.99 (2H,d, *J *= 39.50 Hz, bridging phenyl-C_8_, C_10_), 7.49–7.47 (2H,d, *J *= 8.50 Hz, bridging phenyl-C_7_, C_11_), 7.30–7.26 (4H,m, *J *= 8.00 Hz, phenyl-C_23_, C_24_, C_26,_ C_27_), 7.17–7.16 (2H, m, pyrrole-C_2_, C_5_), 6.40–6.39 (2H, m, pyrrole-C_3_, C_4_), 4.77 (2H, s, oxoethyl-CH_2_-C_14_), 3.87 (3H, s, phenyl-OCH_3_-C_25_), 2.32 (3H, s, phenylethylidene-CH_3_-C_21_).

^13^C NMR (10 mg- CDCl_3_-*d*_*6*_, 100 MHz, T-18.85 ^o^C, *δ* ppm): 167.81, 165.77, 158.61, 141.89, 139.11, 132.01, 129.61, 128.66, 128.02, 123.33, 122.91, 113.12, 107.17, 66.43, 59.07, 15.07.

Mass (ESI-*m/z*) = Found 391.44 (M^+^). Calcd.391.15.

CHN Anal. For C_22_H_21_N_3_O_4_: Calcd.C, 67.51; H, 5.41; N, 10.74; Found. C, 67.48; H, 5.40; N, 10.73.

#### 3.3.22. Synthesis of 2-(2-(1-(4-hydroxyphenyl)ethylidene)hydrazinyl)-2-oxoethyl 4-(1H-pyrrol-1-yl)benzoate (5j):.

Yellow crystalline solid. (Yield 56%). M.p 128–130 °C. FTIR (KBr-cm^-^): 3295 (NH, OH), 2922, 2856 (Ar-C = CH), 1652 (C = O), 1606 (C = O), 1265 (C-O-C).

^1^H NMR (8 mg-CDCl_3_-*d*_*6*_, 400 MHz, T-18.85 ^o^C, *δ* ppm): 10.69 (1H, s, NH), 9.84 (1H, s, phenyl-C_25_-OH), 7.99–7.98 (2H,d, *J *= 6.50 Hz, bridging phenyl-C_8_, C_10_), 7.78–7.77 (2H,d, *J *= 6.50 Hz, bridging phenyl-C_7_, C_11_), 7.74–7.69 (2H,m, phenyl-C_23_, C_27_), 7.53–7.52 (2H,m, phenyl-C_24_, C_26_), 6.86–6.81 (2H, m, pyrrole-C_2_, C_5_), 6.32–6.31 (2H, m, pyrrole-C_3_, C_4_), 4.68 (2H, s, oxoethyl-CH_2_-C_14_), 2.32 (3H, s, phenylethylidene-CH_3_-C_21_).

^13^C NMR (10 mg- CDCl_3_-*d*_*6*_, 100 MHz, T-18.85 ^o^C, *δ* ppm): 168.68, 162.60, 158.84, 156.38, 141.90, 130.61, 129.35, 128.78, 127.98, 118.92, 115.05, 111.03, 68.41, 14.41.

Mass (ESI- *m/z*) = Found 377.14 (M^+^). Calcd. 377.14.

CHN Anal. For C_22_H_19_N_3_O_4_: Calcd.C, 66.83; H, 5.07; N, 11.13; Found. C, 66.82; H, 5.06; N, 11.11.

#### 3.3.23. Synthesis of 2-(2-(1-(3-chlorophenyl)ethylidene)hydrazinyl)-2-oxoethyl 4-(1H-pyrrol-1-yl)benzoate (5k):.

Yellow crystalline solid. (Yield 56%). M.p 162–164 °C. FTIR (KBr-cm^-^): 3309 (NH), 2921, 2857 (Ar-C = CH), 1660 (C = O), 1603 (C = O), 1276 (C-O-C).

^1^H NMR (8 mg-CDCl_3_-*d*_*6*_, 400 MHz, T-18.85 ^o^C, *δ* ppm): 10.85 (1H, s, NH), 7.97–7.95 (2H,d, *J *= 6.50 Hz, bridging phenyl-C_8_, C_10_), 7.78–7.72 (2H,m, bridging phenyl-C_7_, C_11,_ phenyl-C_23,_ C_25_, C_26_, C_27_), 7.75–7.74 (2H, m, pyrrole-C_2_, C_5_), 6.31 (2H, m, pyrrole-C_3_, C_4_), 4.71 (2H, s, oxoethyl-CH_2_-C_14_), 2.37 (3H, s, phenylethylidene-CH_3_-C_21_).

^13^C NMR (10 mg- CDCl_3_-*d*_*6*_, 100 MHz, T-18.85 ^o^C, *δ* ppm): 167.68, 165.38, 158.82, 141.83, 131.86, 130.61, 130.35, 129.01, 128.68, 128.03, 123.68, 113.49, 109.29, 66.59, 14.60.

Mass (ESI- *m/z*) = Found 395.18 (M^+^), 397.21 (M^+^+2). Calcd.395.10.

CHN Anal. For C_21_H_18_ClN_3_O_3_: Calcd.C, 63.72; H, 4.58; N, 10.62; Found. C, 63.70; H, 4.54; N, 10.60.

#### 3.3.24. Synthesis of 2-(2-(1-(4-fluorophenyl)ethylidene)hydrazinyl)-2-oxoethyl 4-(1H-pyrrol-1-yl)benzoate (5l):.

Yellow crystalline solid. (Yield 63%). M.p 134–136 °C. FTIR (KBr-cm^-^): 3330 (NH), 2921, 2857 (Ar-C = CH), 1664 (C = O), 1604 (C = O), 1275 (C-O-C).

^1^H NMR (8 mg-CDCl_3_-*d*_*6*_, 400 MHz, T-18.85 ^o^C, *δ* ppm): 10.79 (1H, s, NH), 7.96–7.95 (2H,d, *J *= 7.00 Hz, bridging phenyl-C_8_, C_10_), 7.90–7.89 (2H,d, *J *= 7.00 Hz, bridging phenyl-C_7_, C_11_), 7.73–7.72 (2H,d, *J *= 8.00 Hz, phenyl-C_23_, C_27_), 7.50–7.49 (2H,m, phenyl-C_24_, C_26_), 7.26 (2H, s, pyrrole-C_2_, C_5_), 6.31–6.30 (2H, m, pyrrole-C_3_, C_4_), 4.69 (2H, s, oxoethyl-CH_2_-C_14_), 2.36 (3H, s, phenylethylidene-CH_3_-C_21_).

^13^C NMR (10 mg- CDCl_3_-*d*_*6*_, 100 MHz, T-18.85 ^o^C, *δ* ppm): 168.08, 166.28, 158.81, 151.39, 141.78, 131.76, 130.61, 130.16, 129.01, 128.66, 128.13, 123.68, 113.50, 109.30, 66.69, 14.67.

Mass (ESI-*m/z*) = Found 379.05 (M^+^), 381.14 (M^+^+2). Calcd.379.13.

CHN Anal. of C_21_H_18_FN_3_O_3_: Calcd.C, 66.48; H, 4.78; N, 11.08; Found. C, 66.45; H, 4.77; N, 11.07.

#### 3.3.25. Synthesis of 2-(2-(1-(2,4-dibromophenyl)ethylidene)hydrazinyl)-2-oxoethyl 4-(1H-pyrrol-1-yl)benzoate (5m):.

Yellow crystalline solid. (Yield 68%). M.p 197–199 °C. FTIR (KBr-cm^-^): 3227 (NH), 2923, 2855 (Ar-C = CH), 1696 (C = O), 1647 (C = O), 1233 (C-O-C).

^1^H NMR (8 mg-CDCl_3_-*d*_*6*_, 400 MHz, T-18.85 ^o^C, *δ* ppm): 10.64 (1H, s, NH), 8.29–8.27 (2H,d, *J *= 8.40 Hz, bridging phenyl-C_8_, C_10_), 8.19–8.17 (2H,d, *J *= 8.00 Hz, bridging phenyl-C_7_, C_11_), 8.11–8.09 (1H,d, *J *= 8.00 Hz, phenyl-C_27_), 7.67–7.65 (2H,d, *J *= 8.00 Hz, phenyl-C_24_, C_26_), 7.62–7.47 (2H, m, pyrrole-C_2_, C_5_), 6.39 (2H, m, pyrrole-C_3_, C_4_), 4.39–4.38 (2H, d, *J *= 6.40 Hz, oxoethyl-CH_2_-C_14_), 2.29 (3H, s, phenylethylidene-CH_3_-C_21_).

^13^C NMR (10 mg- CDCl_3_-*d*_*6*_, 100 MHz, T-18.85 ^o^C, *δ* ppm): 170.06, 169.56, 153.55, 143.83, 140.70, 140.06, 133.04, 131.61, 131.45, 129.22, 128.43, 128.17, 126.93, 124.30, 122.33, 121.76, 118.75, 113.14, 61.56, 24.57.

Mass (ESI- *m/z*) = Found 518.98 (M^+^), 520.67 (M^+^+2), 524.37 (M^+^+4). Calcd. 518.96.

CHN Anal. For C_21_H_17_Br_2_N_3_O_3_: Calcd.C, 48.58; H, 3.30; Br, 30.78; N, 8.09; Found. C, 48.58; H, 3.30; Br, 30.78; N, 8.09.

#### 3.3.26. Synthesis of 2-(2-(1-(2,4-dichlorophenyl)ethylidene)hydrazinyl)-2-oxoethyl 4-(1H-pyrrol-1-yl)benzoate (5n):.

Yellow crystalline solid. (Yield 72%). M.p 194–196 °C. FTIR (KBr-cm^-^): 3391 (NH), 2920, 2858 (Ar-C = CH), 1698 (C = O), 1653 (C = O), 1235 (C-O-C).

^1^H NMR (8 mg-CDCl_3_-*d*_*6*_, 400 MHz, T-18.85 ^o^C, *δ* ppm): 10.60 (1H, s, NH), 8.12–8.10 (2H,d, *J *= 7.60 Hz, bridging phenyl-C_8_, C_10_), 7.98–7.96 (2H,d, *J *= 11.20 Hz, bridging phenyl-C_7_, C_11_), 7.44–7.42 (2H,d, *J *= 7.20 Hz, phenyl-C_27_), 7.41–7.32 (2H,m, phenyl-C_24_, C_26_), 7.19–7.15 (2H, m, pyrrole-C_2_, C_5_), 6.38–6.36 (2H, m, pyrrole-C_3_, C_4_), 4.53–4.50 (2H, d, *J *= 9.20 Hz, oxoethyl-CH_2_-C_14_), 2.35 (3H, s, phenylethylidene-CH_3_-C_21_).

^13^C NMR (10 mg- CDCl_3_-*d*_*6*_, 100 MHz, T-18.85 ^o^C, *δ* ppm): 171.09, 168.00, 158.98, 144.92, 139.29, 133.60, 132.52, 129.68, 128.88, 119.27, 115.51, 111.85, 55.67, 24.27.

Mass (ESI- *m/z*) = Found 429.06 (M^+^), 431.28 (M^+^+2). Calcd.429.06.

CHN Anal. For C_21_H_17_Cl_2_N_3_O_3_: Calcd.C, 58.62; H, 3.98; N, 9.77; Found. C, 58.61; H, 3.96; N, 9.74.

#### 3.3.27. Synthesis of 2-(2-(1-(4-aminophenyl)ethylidene)hydrazinyl)-2-oxoethyl 4-(1H-pyrrol-1-yl)benzoate (5o):.

Yellow crystalline solid. (Yield 43%). M.p 154–156 °C. FTIR (KBr-cm^-^): 3529 (NH_2_), 3350 (NH), 2921, 2856 (Ar-C = CH), 1700 (C = O), 1653 (C = O), 1251 (C-O-C).

^1^H NMR (8 mg-CDCl_3_-*d*_*6*_, 400 MHz, T-18.85 ^o^C, *δ* ppm): 10.89 (1H, s, NH), 7.81–7.79 (2H,d, *J *= 8.80 Hz, bridging phenyl-C_8_, C_10_), 7.76–7.74 (2H,d, *J *= 7.50 Hz, bridging phenyl-C_7_, C_11_), 7.70–7.69 (2H,d, *J *= 8.40 Hz, phenyl-C_23_, C_27_), 7.49–7.47 (2H,d, *J *= 8.00 Hz, phenyl-C_24_, C_26_), 7.25–7.17 (2H, m, pyrrole-C_2_, C_5_), 6.39 (2H, s, pyrrole-C_3_, C_4_), 4.69 (2H, s, oxoethyl-CH_2_-C_14_), 5.59 (3H, s, phenyl-NH_2_-C_28_), 2.30 (3H, s, phenylethylidene-CH_3_-C_21_).

^13^C NMR (10 mg- CDCl_3_-*d*_*6*_, 100 MHz, T-18.85 ^o^C, *δ* ppm): 170.29, 167.90, 158.97, 144.88, 138.29, 134.66, 132.25, 131.35, 129.71, 128.81, 119.28, 111.85, 56.67, 24.42.

Mass (ESI- *m/z*) = Found 376.18 (M^+^). Calcd. 376.15.

CHN Anal. For C_21_H_20_N_4_O_3_: Calcd.C, 67.01; H, 5.36; N, 14.88; Found: C, 66.97; H, 5.33; N, 14.85.

#### 3.3.28. Synthesis of 2-(2-(1-(3-hydroxyphenyl)ethylidene)hydrazinyl)-2-oxoethyl 4-(1H-pyrrol-1-yl)benzoate (5p):.

Yellow crystalline solid. (Yield 48%). M.p 142–144 °C. FTIR (KBr-cm^-^): 3351 (NH), 3253 (OH), 2923, 2854 (Ar-C = CH), 1679 (C = O), 1634 (C = O), 1323 (C-O-C).

^1^H NMR (8 mg-CDCl_3_-*d*_*6*_, 400 MHz, T-18.85 ^o^C, *δ* ppm): 10.72 (1H, s, NH), 9.51 (1H, s, OH), 7.98–7.96 (2H,d, *J *= 7.60 Hz, bridging phenyl-C_8_, C_10_), 7.77–7.75 (2H,d, *J *= 5.20 Hz, bridging phenyl-C_7_, C_11_), 7.74–7.72 (2H,d, *J *= 8.80 Hz, phenyl-C_23_, C_27_), 7.70–7.68 (2H,d, *J *= 7.20 Hz, phenyl-C_24_, C_26_), 7.33–7.17 (2H, m, pyrrole-C_2_, C_5_), 6.32–6.31 (2H, m, pyrrole-C_3_, C_4_), 4.45 (2H, s, oxoethyl-CH_2_-C_14_), 2.35–2.33 (3H, d, *J *= 8.00 Hz, phenylethylidene-CH_3_-C_21_).

^13^C NMR (10 mg- CDCl_3_-*d*_*6*_, 100 MHz, T-18.85 ^o^C, *δ* ppm):171.29, 167.07, 159.98, 145.02, 139.32, 133.63, 132.53, 131.40, 129.67, 128.82, 119.96, 115.61, 111.75, 56.97, 26.62.

Mass (ESI- *m/z*) = Found 377.17 (M^+^). Calcd. 377.14.

CHN Anal. For C_21_H_19_N_3_O_4_: Calcd.C, 66.83; H, 5.07; N, 11.13; Found. C, 66.80; H, 5.04; N, 11.12.

#### 3.3.29. Synthesis of 2-(2-benzylidenehydrazinyl)-2-oxoethyl 4-(1H-pyrrol-1-yl)benzoate (6a).

White crystalline solid. (Yield 52%). M.p 150–152 °C. FTIR (KBr-cm^-^): 3271 (NH), 2923, 2854 (Ar-C = CH), 1654 (C = O), 1607 (C = O), 1286 (C-O-C).

^1^H NMR (8 mg-CDCl_3_-*d*_*6*_, 400 MHz, T-18.85 ^o^C, *δ* ppm): 11.91 (1H, s, NH), 8.49 (1H, s, benzylidene-H-C_21_), 8.04–8.02 (2H,d, *J *= 8.60 Hz, bridging phenyl-C_8_, C_10_), 7.79–7.77 (2H,d, *J *= 8.84 Hz, bridging phenyl-C_7_, C_11_), 7.68–7.66 (3H,m, phenyl-C_23_, C_25_, C_27_), 7.66–7.63 (2H, m, pyrrole-C_2_, C_5_), 7.48–7.46 (2H,d, *J *= 7.20 Hz, phenyl-C_24_, C_26_), 6.33–6.32 (2H, m, pyrrole-C_3_, C_4_), 4.41 (2H, s, oxoethyl-CH_2_-C_14_).

^13^C NMR (10 mg- CDCl_3_-*d*_*6*_, 100 MHz, T-18.85 ^o^C, *δ* ppm): 173.47, 168.32, 162.24, 147.69, 143.30, 142.23, 134.32, 130.06, 129.95, 128.83, 127.06, 126.99, 119.00, 111.19, 61.23.

Mass (ESI- *m/z*) = Found 347.12 (M^+^). Calcd.347.13.

CHN Anal. For C_20_H_17_N_3_O_3_: Calcd.C, 69.15; H, 4.93; N, 12.10; Found. C, 69.12; H, 4.91; N, 11.08.

#### 3.3.30. Synthesis of 2-(2-(2-bromobenzylidene)hydrazinyl)-2-oxoethyl 4-(1H-pyrrol-1-yl)benzoate (6b).

White crystalline solid. (Yield 66%). M.p 166–168 °C. FTIR (KBr-cm^-^): 3223 (NH), 2922, 2855 (Ar-C = CH), 1690 (C = O), 1651 (C = O), 1244 (C-O-C).

^1^H NMR (8 mg-CDCl_3_-*d*_*6*_, 400 MHz, T-18.85 ^o^C, *δ* ppm): 12.14 (1H, s, NH), 8.84 (1H, s, benzylidene-H-C_21_), 8.05–8.03 (2H,d, *J *= 8.40 Hz, bridging phenyl-C_8_, C_10_), 7.79–7.77 (2H,d, *J *= 8.00 Hz, bridging phenyl-C_7_, C_11_), 7.71–7.69 (2H,d, *J *= 8.80 Hz, phenyl-C_26_, C_27_), 7.53–7.46 (2H, m, pyrrole-C_2_, C_5_), 7.39–7.37 (2H,d, *J *= 7.20 Hz, phenyl-C_24_, C_25_), 6.32 (2H, s, pyrrole-C_3_, C_4_), 4.04 (2H, s, oxoethyl-CH_2_-C_14_).

^13^C NMR (10 mg- CDCl_3_-*d*_*6*_, 100 MHz, T-18.85 ^o^C, *δ* ppm): 173.37, 168.28, 152.78, 138.45, 135.91, 132.64, 130.99, 129.67, 128.91, 124.38, 123.24, 119.36, 112.52, 62.63.

Mass (ESI- *m/z*) = Found 426.24 (M^+^), 428.20 (M^+^+2). Calcd. 426.26.

CHN Anal. For C_20_H_16_BrN_3_O_3_: Calcd.C, 56.35; H, 3.78; N, 9.86; Found. C, 56.32; H, 3.77; N, 9.85.

#### 3.3.31. Synthesis of 2-(2-(4-nitrobenzylidene)hydrazinyl)-2-oxoethyl 4-(1H-pyrrol-1-yl)benzoate (6c).

White crystalline solid. (Yield 62%). M.p 158–160 °C. FTIR (KBr-cm^-^): 3243 (NH), 2921, 2854 (Ar-C = CH), 1683 (C = O), 1629 (C = O), 1318 (C-O-C).

^1^H NMR (8 mg-CDCl_3_-*d*_*6*_, 400 MHz, T-18.85 ^o^C, *δ* ppm): 12.23 (1H, s, NH), 8.89 (1H, s, benzylidene-H-C_21_), 8.15–8.08 (2H,m, bridging phenyl-C_8_, C_10_), 8.05–8.03 (2H,d, *J *= 8.40 Hz, bridging phenyl-C_7_, C_11_), 7.85–7.71 (2H,m, phenyl-C_23_, C_27_), 7.54–7.53 (2H, m, pyrrole-C_2_, C_5_), 7.69–7.67 (2H,d, *J *= 8.00 Hz, phenyl-C_24_, C_26_), 6.33–6.32 (2H, m, pyrrole-C_3_, C_4_), 4.43 (2H, s, oxoethyl-CH_2_-C_14_).

^13^C NMR (10 mg- CDCl_3_-*d*_*6*_, 100 MHz, T-18.85 ^o^C, *δ* ppm): 169.04, 166.72, 152.95, 143.23, 137.46, 136.79, 135.93, 131.01, 130.88, 129.06, 128.70, 127.47, 127.29, 123.88, 121.49, 118.91, 112.57, 110.79, 65.66.

Mass (ESI- *m/z*) = Found 392.12 (M^+^+H). Calcd. 392.11.

CHN Anal. For C_20_H_16_N_4_O_5_: Calcd.C, 61.22; H, 4.11; N, 14.28; Found.C, 61.21; H, 4.11; N, 14.27.

#### 3.3.32. Synthesis of 2-(2-(4-chlorobenzylidene)hydrazinyl)-2-oxoethyl 4-(1H-pyrrol-1-yl)benzoate (6d).

White crystalline solid. (Yield 70%). M.p 162–164 °C. FTIR (KBr-cm^-^): 3384 (NH), 2925, 2859 (Ar-C = CH), 1681 (C = O), 1626 (C = O), 1312 (C-O-C).

^1^H NMR (8 mg-CDCl_3_-*d*_*6*_, 400 MHz, T-18.85 ^o^C, *δ* ppm): 11.95 (1H, s, NH), 8.46 (1H, s, benzylidene-H-C_21_), 8.02–8.00 (2H,d, *J *= 8.40 Hz, bridging phenyl-C_8_, C_10_), 7.91–7.89 (2H,d, *J *= 6.80 Hz, bridging phenyl-C_7_, C_11_), 7.78–7.76 (2H,d, *J *= 8.40 Hz, phenyl-C_23_, C_27_), 7.60–7.57 (2H,m, phenyl-C_24_, C_26_), 7.57–7.49 (2H, m, pyrrole-C_2_, C_5_), 6.32–6.31 (2H, m, pyrrole-C_3_, C_4_), 4.49 (2H, s, oxoethyl-CH_2_-C_14_).

^13^C NMR (10 mg- CDCl_3_-*d*_*6*_, 100 MHz, T-18.85 ^o^C, *δ* ppm): 167.37, 165.90, 156.01, 144.38, 142.28, 132.52, 131.87, 130.80, 129.14, 128.51, 127.44, 124.42, 118.97, 111.22, 65.66.

Mass (ESI- *m/z*) = Found 381.10 (M^+^), 383.20 (M^+^+2). Calcd.381.09.

CHN Anal. For C_20_H_16_ClN_3_O_3_: Calcd.C, 62.92; H, 4.22; N, 11.01; Found.C, 62.90; H, 4.21; N, 11.01.

#### 3.3.33. Synthesis of 2-(2-(4-methoxybenzylidene)hydrazinyl)-2-oxoethyl 4-(1H-pyrrol-1-yl)benzoate (6e).

White crystalline solid. (Yield 76%). M.p 172–174 °C. FTIR (KBr-cm^-^): 3351 (NH), 2923, 2855 (Ar-C = CH), 1709 (C = O), 1635 (C = O), 1314 (C-O-C).

^1^H NMR (8 mg-CDCl_3_-*d*_*6*_, 400 MHz, T-18.85 ^o^C, *δ* ppm): 11.74 (1H, s, NH),8.31 (1H, **s,** benzylidene-H-C_21_**),** 8.01–7.99 (2H,d, *J *= 8.80 Hz, bridging phenyl-C_8_, C_10_), 7.82–7.80 (2H,d, *J *= 8.80 Hz, bridging phenyl-C_7_, C_11_), 7.77–7.74 (2H,d, *J *= 8.80 Hz, phenyl-C_23_, C_27_), 7.69–7.67 (2H,d, *J *= 7.20 Hz, phenyl-C_24_, C_26_), 7.52–7.51 (2H, d, *J *= 2.00 Hz pyrrole-C_2_, C_5_), 6.32–6.31 (2H, m, pyrrole-C_3_, C_4_), 4.37 (2H, s, oxoethyl-CH_2_-C_14_), 3.8–3.78 (3H, s, phenyl-OCH_3_-C_25_).

^13^C NMR (10 mg- CDCl_3_-*d*_*6*_, 100 MHz, T-18.85 ^o^C, *δ* ppm): 167.37, 164.71, 157.74, 142.26, 138.62, 136.27, 134.42, 132.60, 129.16, 128.53, 122.82, 118.82, 112.45, 65.99, 56.69.

Mass (ESI- *m/z*) = Found 377.13 (M^+^). Calcd.377.14.

CHN Anal. For C_21_H_19_N_3_O_4_: Calcd.C, 66.83; H, 5.07; N, 11.13; Found.C, 66.82; H, 5.06; N, 11.10.

### 3.4 Molecular docking using surflex-dock

The study employed the patented Sybyl-X 2.0 search tool and Surflex-Dock for molecular docking analysis. The aim of this study was to provide a comprehensive understanding of the molecular interactions between chemicals and the active sites of the ENR enzyme and DHFR enzyme [22]. This work provides a thorough analysis that can be applied to enhance the future optimization of molecular architectures. The crystallographic structures of enoyl acyl carrier protein reductase InhA, in complex with 1-cyclohexyl-*N*-(3,5-dichlorophenyl)-5-oxopyrrolidine-3-carboxamide (PDB ID:4TZK, resolution of 1.62 Å by X-ray diffraction), and dihydrofolate reductase of *Mycobacterium tuberculosis*, bound to NADPH and methotrexate (PDB ID:1DF7, resolution of 1.70 Å by X-ray diffraction), were obtained from the Brookhaven Protein Database (PDB) located at http://www.rcsb.org/pdb and protein preparation was done by using Sybyl-X 2.0 standard protocol. The ligands and protein employed in our docking methods were produced using the known Sybyl-X 2.0 standard protocol [23,24]. The inclusion of hydrogen atoms was necessary in order to establish the accurate configuration and tautomeric states. Subsequently, the structural model underwent energy minimization via the Tripos force field, incorporating a distance-dependent dielectric function. Partial atomic charges were then computed using the AM-BER7F9902 method. Lastly, the model was purged of water molecules. The molecular geometry of CP was later refined to achieve minimal energy by the utilization of the Powell energy minimization method. This process involved employing the Tripos force field together with Gasteiger-Hückel charges. Subsequently, the CP molecule was individually inserted into the binding pocket to facilitate the investigation of docking and scoring. In order to ascertain the interactions between the ligand and protein, the highest-ranking posture and protein were imported into the working environment. The MOLCAD application, which is a tool for molecular computer-aided design, was utilized to depict the manner in which the protein and ligand bind together.

### 3.5. ADMET studies (Table S1 and Table S2 in S2 File)

The toxicities were predicted using ProTox-II, and the corresponding results are shown in Table S1 in S2 File. Additionally, the Molecular ADME properties were estimated using the in silico Swiss ADME online tool [25], and the results are presented in Table S2 in S2 File.

### 3.6. MTT based cytotoxic activity

The cytotoxic activity (IC_50_) of selected compounds against A549 (lung adenocarcinoma) MV cell-lines was evaluated by performing cellular conversion of MTT [3-(4,5-dimethylthiazo-2-yl)-2,5-diphenyl-tetrazolium bromide] into a formazan product. This evaluation was conducted up to a concentration of 50 mg/mL using the Promega Cell Titre 96 non-radioactive cell proliferation assay, with cisplatin serving as the positive control. Cytotoxicity is commonly quantified by determining the IC_50_ value, which represents the quantity of a substance that reduces the optical density of treated cells by 50% compared to untreated cells, as measured by the MTT experiment. The results are presented as IC_50_ values (mean ± standard error of the mean (SEM) obtained from three separate and independent measurements [26].

### 3.7. Antitubercular activity

The efficacy of the newly synthesized compounds was assessed against the *M. tuberculosis* strain H37Rv (Council of Scientific and Industrial Research (CSIR), India) using the Microplate Alamar Blue test (MABA). The obtained data, including the minimum inhibitory concentration (MIC) values, are presented in Table 3 [27].

### 3.8. Antibacterial activity

The antibacterial inhibitory effects of all compounds were assessed using the broth microdilution method, with ciprofloxacin serving as the reference medication. The study focused on comparing the inhibitory effects against *S. aureus* (Gram positive- ATCC 29213) and *E. coli* (Gram negative- ATCC 25922) bacteria [28–31]. The antibacterial activity data, including the minimum inhibitory concentration (MIC) values, was summarized in Table 3.

### 3.9. Ethical statement

Not applicable, since the study does not involve any research done on animals or humans.

## 4. Conclusion

The antitubercular and enzyme inhibitory effects of 28 newly synthesized pyrrolyl-benzoate derivatives were assessed. All the compounds had good potency against *M. tuberculosis*, as shown by MICs ranging from 0.8 to 3.12 µg/mL. The derivatives were subjected to molecular docking analysis, revealing that these newly identified inhibitors exhibited a close match inside the binding site of both the ENR-enzyme and DHFR enzyme, similar to the ML ligand and 1DF7 ligand. *In vitro* assays indicated that the compounds **4b, 4f, 5a, 5j, 5k, 6a, 6c** and **6d**, have significant enzyme inhibitory action (against both enzymes). It is therefore proposed that chemical scaffolds have generated new single molecules that exert antitubercular activity, at least partly through targeting DHFR and ENR-enzymes. We anticipate that the analogues disclosed in this work will aid global efforts to identify prospective lead compounds for further development of the new entities with dual DHFR and ENR-enzyme inhibitory properties.

## Supporting information

S1 File Spectrum files of reported compounds. (DOCX)

S2 File Table and Figure file for docked mode of all the 28 synthesized compounds at InhA active site of (PDB ID 4TZK) and (PDB ID1DF7). (DOC)
