# Supplementary material for: Design, synthesis and in silico molecular modelling studies of 2-Hydrazineyl-2-oxoethyl-4-(1H-pyrrol-1-yb) benzoate derivatives: a potent dual DHFR and ENR-reductase inhibitors with antitubercular, antibacterial and cytotoxic potential
Source: PLoS One. 2025 May 19;20(5):e0323702. doi: 10.1371/journal.pone.0323702 (PMC12088000; doi:10.1371/journal.pone.0323702)
Supplement: S1 File — (DOCX) [file pone.0323702.s001.docx]

**Design, Synthesisiandi*IniSilico*iMoleculariModelling StudiesiofiNovel Seriesiofi2-Hydrazineyl-2-oxoethyl-4-(1*H*-pyrrol-1-yb) Benzoate derivatives: A Potent Dual DHFR And ENR-Reductase Inhibitors with Antitubercular, Antibacterial and Cytotoxic Potential.**

**Table S1: Swiss ADME web tool's ADME properties for synthetic molecules**

| **Compound** | **Log P** | **Molar refractivity** | **TPSA** | **HBA** | **HBD** | **RB** | **GI Absorption** | **BBB Permeant** | **Log Kp cm/s** | **Solubility** | **CYP inhibitor** | | | | | **Lipinski violation** | **Synthetic accessibility** |
| --- | --- | --- | --- | --- | --- | --- | --- | --- | --- | --- | --- | --- | --- | --- | --- | --- | --- |
|  |  |  |  |  |  |  |  |  |  |  | **1A2** | **2C19** | **2C9** | **2D6** | **3A4** |  |  |
| **4a** | 2.27 | 105.45 | 103.16 | 5 | 1 | 6 | High | No | -6.98 | Soluble | No | Yes | Yes | No | No | 0 | 2.96 |
| **4b** | 2.35 | 125.49 | 103.16 | 5 | 1 | 6 | High | No | -6.04 | Poorly Soluble | Yes | Yes | Yes | No | No | 2 | 3.03 |
| **4c** | 2.88 | 136.25 | 103.16 | 5 | 1 | 6 | High | No | -6.94 | Poorly Soluble | Yes | Yes | No | No | No | 2 | 3.14 |
| **4d** | 2.01 | 93.10 | 97.71 | 5 | 1 | 6 | High | No | -7.93 | Soluble | No | No | No | No | No | 0 | 2.54 |
| **4e** | 2.05 | 87.94 | 103.16 | 5 | 1 | 6 | High | No | -7.68 | Soluble | No | Yes | No | No | No | 0 | 2.72 |
| **4f** | 2.23 | 92.91 | 103.16 | 5 | 1 | 6 | High | No | -7.50 | Soluble | No | Yes | No | No | No | 0 | 2.94 |
| **4g** | 2.05 | 97.43 | 97.71 | 5 | 1 | 6 | High | No | -7.56 | Soluble | No | No | No | No | No | 0 | 2.80 |
| **5a** | 3.31 | 102.64 | 72.69 | 4 | 1 | 8 | High | Yes | -6.11 | Moderately Soluble | No | Yes | Yes | No | No | 0 | 2.89 |
| **5b** | 3.77 | 110.34 | 72.69 | 4 | 1 | 8 | High | Yes | -6.10 | Moderately Soluble | Yes | Yes | Yes | No | Yes | 0 | 2.98 |
| **5c** | 3.52 | 107.65 | 72.69 | 4 | 1 | 8 | High | Yes | -5.87 | Moderately Soluble | Yes | Yes | Yes | No | Yes | 0 | 2.92 |
| **5d** | 2.89 | 111.47 | 118.51 | 6 | 1 | 9 | High | No | -6.51 | Moderately Soluble | No | Yes | Yes | No | Yes | 0 | 3.09 |
| **5e** | 3.66 | 107.61 | 72.69 | 4 | 1 | 8 | High | Yes | -5.93 | Moderately Soluble | No | Yes | Yes | No | Yes | 0 | 3.00 |
| **5f** | 3.82 | 110.34 | 72.69 | 4 | 1 | 8 | High | Yes | -6.10 | Moderately Soluble | Yes | Yes | Yes | No | Yes | 0 | 3.04 |
| **5g** | 2.86 | 111.47 | 118.51 | 6 | 1 | 9 | High | No | -6.51 | Moderately Soluble | No | Yes | Yes | No | Yes | 0 | 3.14 |
| **5h** | 3.15 | 109.14 | 81.92 | 5 | 1 | 9 | High | No | -6.32 | Moderately Soluble | No | Yes | Yes | No | Yes | 0 | 3.03 |
| **5i** | 3.57 | 109.14 | 81.92 | 5 | 1 | 9 | High | No | -6.32 | Moderately Soluble | No | Yes | Yes | No | Yes | 0 | 2.99 |
| **5j** | 2.95 | 104.67 | 92.92 | 5 | 2 | 8 | High | No | -6.46 | Moderately Soluble | No | No | Yes | No | No | 0 | 2.90 |
| **5k** | 3.69 | 107.65 | 72.69 | 4 | 1 | 8 | High | Yes | -5.87 | Moderately Soluble | Yes | Yes | Yes | No | Yes | 0 | 2.93 |
| **5l** | 3.36 | 102.60 | 72.69 | 5 | 1 | 8 | High | Yes | -6.15 | Moderately Soluble | No | Yes | Yes | No | Yes | 0 | 2.91 |
| **5m** | 3.91 | 118.04 | 72.69 | 4 | 1 | 8 | High | No | -6.09 | Moderately Soluble | Yes | Yes | Yes | No | Yes | 0 | 3.11 |
| **5n** | 3.62 | 112.66 | 72.69 | 4 | 1 | 8 | High | Yes | -5.64 | Moderately Soluble | Yes | Yes | Yes | No | Yes | 0 | 3.06 |
| **5o** | 2.96 | 107.05 | 98.71 | 4 | 2 | 8 | High | No | -6.69 | Soluble | No | No | Yes | No | No | 0 | 2.97 |
| **5p** | 2.93 | 104.67 | 92.92 | 5 | 2 | 8 | High | No | -6.46 | Moderately Soluble | No | No | Yes | No | No | 0 | 2.91 |
| **6a** | 3.17 | 97.84 | 72.69 | 4 | 1 | 8 | High | Yes | -6.18 | Soluble | No | Yes | Yes | No | Yes | 0 | 2.83 |
| **6b** | 3.50 | 105.54 | 72.69 | 4 | 1 | 8 | High | Yes | -6.17 | Moderately Soluble | Yes | Yes | Yes | No | Yes | 0 | 2.93 |
| **6c** | 2.67 | 106.66 | 118.51 | 6 | 1 | 9 | High | No | -6.58 | Soluble | No | Yes | Yes | No | Yes | 0 | 3.02 |
| **6d** | 3.27 | 102.85 | 72.69 | 4 | 1 | 8 | High | Yes | -5.95 | Moderately Soluble | Yes | Yes | Yes | No | No | 0 | 2.85 |
| **6e** | 3.21 | 104.33 | 81.92 | 5 | 1 | 9 | High | No | -6.39 | Moderately Soluble | No | Yes | Yes | No | Yes | 0 | 2.91 |

**Table S2: Toxicity studies of synthesized compounds**

| **Compound code** | **LD50**  **mg/kg** | **Hepatotoxicity** | **Carcinogenicity** | **Immunotoxicity** | **Mutagenicity** | **Cytotoxicity** | **Aryl hydrocarbon Receptor** | **Androgen Receptor (AR)** | **Androgen Receptor Ligand Binding Domain** | **Aromatase** | **Estrogen Receptor Ligand Binding Domain** | **Peroxisome Proliferator Activated Receptor Gamma** | **Nuclear factor** | **Heat shock factor response element** | **Mitochondrial Membrane Potential** | **Phosphoprotein** | **ATPase family AAA domain containing protein 5** |
| --- | --- | --- | --- | --- | --- | --- | --- | --- | --- | --- | --- | --- | --- | --- | --- | --- | --- |
| **4a** | 750 | Active | Inactive | Inactive | Inactive | Inactive | Inactive | Inactive | Inactive | Inactive | Inactive | Inactive | Inactive | Inactive | Inactive | Inactive | Inactive |
| **4b** | 750 | Active | Inactive | Inactive | Inactive | Inactive | Inactive | Inactive | Inactive | Inactive | Inactive | Inactive | Inactive | Inactive | Inactive | Inactive | Inactive |
| **4c** | 750 | Active | Inactive | Inactive | Inactive | Inactive | Inactive | Inactive | Inactive | Inactive | Inactive | Inactive | Inactive | Inactive | Inactive | Inactive | Inactive |
| **4d** | 5000 | Inactive | Inactive | Inactive | Inactive | Inactive | Inactive | Inactive | Inactive | Inactive | Inactive | Inactive | Inactive | Inactive | Inactive | Inactive | Inactive |
| **4e** | 750 | Active | Inactive | Inactive | Inactive | Inactive | Inactive | Inactive | Inactive | Inactive | Inactive | Inactive | Inactive | Inactive | Inactive | Inactive | Inactive |
| **4f** | 540 | Active | Inactive | Inactive | Inactive | Inactive | Inactive | Inactive | Inactive | Inactive | Inactive | Inactive | Inactive | Inactive | Inactive | Inactive | Inactive |
| **4g** | 2300 | Active | Inactive | Inactive | Inactive | Inactive | Inactive | Inactive | Inactive | Inactive | Inactive | Inactive | Inactive | Inactive | Inactive | Inactive | Inactive |
| **5a** | 1000 | Active | Active | Inactive | Inactive | Inactive | Inactive | Inactive | Inactive | Inactive | Inactive | Inactive | Inactive | Inactive | Inactive | Inactive | Inactive |
| **5b** | 1000 | Active | Inactive | Inactive | Inactive | Inactive | Inactive | Inactive | Inactive | Inactive | Inactive | Inactive | Inactive | Inactive | Inactive | Inactive | Inactive |
| **5c** | 1830 | Active | Inactive | Inactive | Inactive | Inactive | Inactive | Inactive | Inactive | Inactive | Inactive | Inactive | Inactive | Inactive | Inactive | Inactive | Inactive |
| **5d** | 5000 | Active | Active | Inactive | Active | Inactive | Inactive | Inactive | Inactive | Inactive | Inactive | Inactive | Inactive | Inactive | Inactive | Inactive | Inactive |
| **5e** | 1000 | Active | Active | Inactive | Inactive | Inactive | Inactive | Inactive | Inactive | Inactive | Inactive | Inactive | Inactive | Inactive | Inactive | Inactive | Inactive |
| **5f** | 1000 | Active | Inactive | Inactive | Inactive | Inactive | Inactive | Inactive | Inactive | Inactive | Inactive | Inactive | Inactive | Inactive | Inactive | Inactive | Inactive |
| **5g** | 3000 | Active | Active | Inactive | Active | Inactive | Inactive | Inactive | Inactive | Inactive | Inactive | Inactive | Inactive | Inactive | Inactive | Inactive | Inactive |
| **5h** | 4920 | Active | Inactive | Inactive | Inactive | Inactive | Inactive | Inactive | Inactive | Inactive | Inactive | Inactive | Inactive | Inactive | Inactive | Inactive | Inactive |
| **5i** | 1120 | Active | Inactive | Inactive | Inactive | Inactive | Inactive | Inactive | Inactive | Inactive | Inactive | Inactive | Inactive | Inactive | Inactive | Inactive | Inactive |
| **5j** | 1000 | Active | Inactive | Inactive | Inactive | Inactive | Inactive | Inactive | Inactive | Inactive | Inactive | Inactive | Inactive | Inactive | Inactive | Inactive | Inactive |
| **5k** | 1830 | Active | Inactive | Inactive | Inactive | Inactive | Inactive | Inactive | Inactive | Inactive | Inactive | Inactive | Inactive | Inactive | Inactive | Inactive | Inactive |
| **5l** | 3000 | Active | Inactive | Inactive | Inactive | Inactive | Inactive | Inactive | Inactive | Inactive | Inactive | Inactive | Inactive | Inactive | Inactive | Inactive | Inactive |
| **5m** | 1000 | Active | Inactive | Inactive | Inactive | Inactive | Inactive | Inactive | Inactive | Inactive | Inactive | Inactive | Inactive | Inactive | Inactive | Inactive | Inactive |
| **5n** | 1830 | Active | Inactive | Inactive | Inactive | Inactive | Inactive | Inactive | Inactive | Inactive | Inactive | Inactive | Inactive | Inactive | Inactive | Inactive | Inactive |
| **5o** | 710 | Active | Inactive | Inactive | Inactive | Inactive | Inactive | Inactive | Inactive | Inactive | Inactive | Inactive | Inactive | Inactive | Inactive | Inactive | Inactive |
| **5p** | 4540 | Active | Inactive | Inactive | Inactive | Inactive | Inactive | Inactive | Inactive | Inactive | Inactive | Inactive | Inactive | Inactive | Inactive | Inactive | Inactive |
| **6a** | 3000 | Active | Active | Inactive | Inactive | Inactive | Inactive | Inactive | Inactive | Inactive | Inactive | Inactive | Inactive | Inactive | Inactive | Inactive | Inactive |
| **6b** | 3000 | Active | Inactive | Inactive | Inactive | Inactive | Inactive | Inactive | Inactive | Inactive | Inactive | Inactive | Inactive | Inactive | Inactive | Inactive | Inactive |
| **6c** | 5000 | Active | Active | Inactive | Active | Inactive | Inactive | Inactive | Inactive | Inactive | Inactive | Inactive | Inactive | Inactive | Inactive | Inactive | Inactive |
| **6d** | 5000 | Active | Inactive | Inactive | Inactive | Inactive | Inactive | Inactive | Inactive | Inactive | Inactive | Inactive | Inactive | Inactive | Inactive | Inactive | Inactive |
| **6e** | 1120 | Active | Inactive | Inactive | Inactive | Inactive | Inactive | Inactive | Inactive | Inactive | Inactive | Inactive | Inactive | Inactive | Inactive | Inactive | Inactive |

**Spectrum 1: IR Spectrum of compound 1**
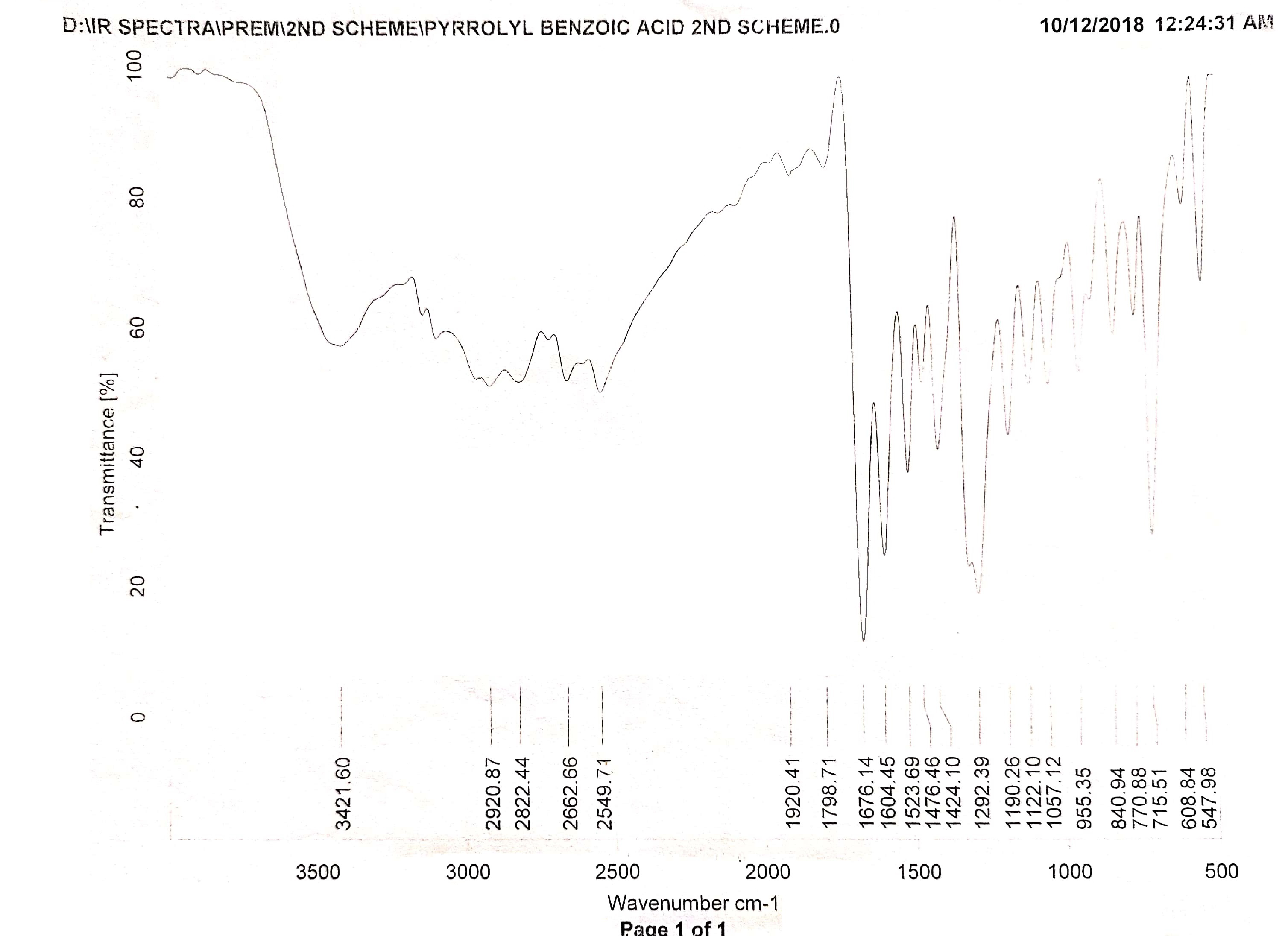

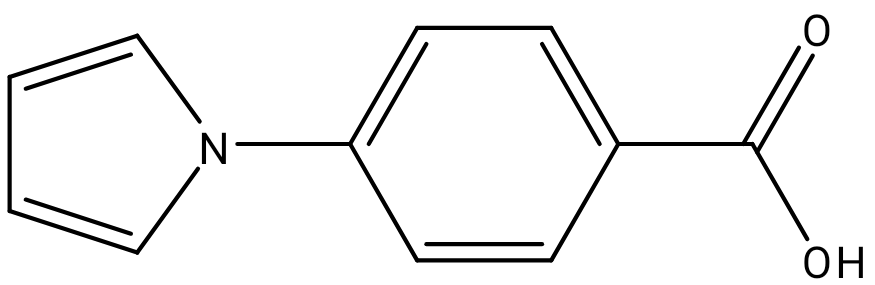


**Spectrum 2: IR Spectrum of compound 2**


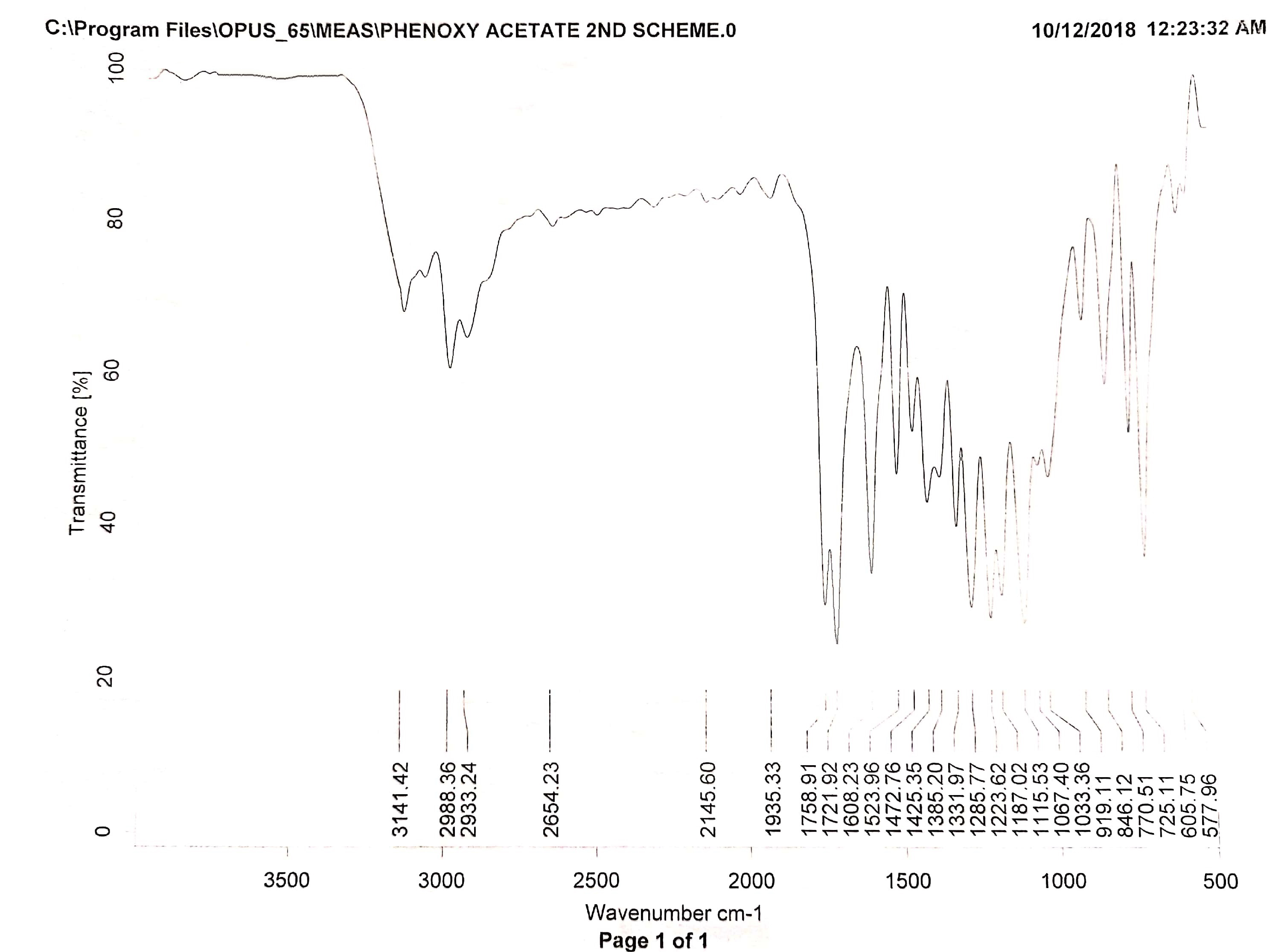

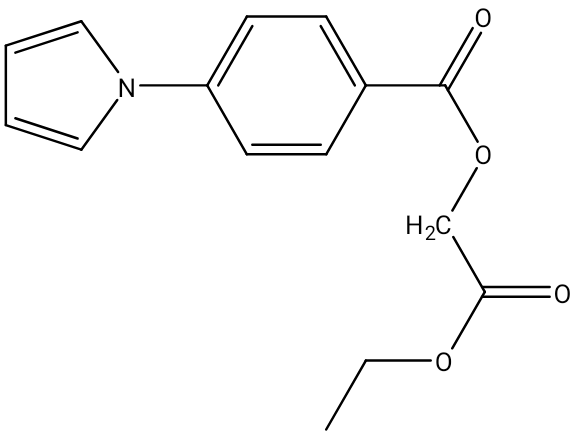


**Spectrum 3: ^1^H NMR Spectrum of compound 2**


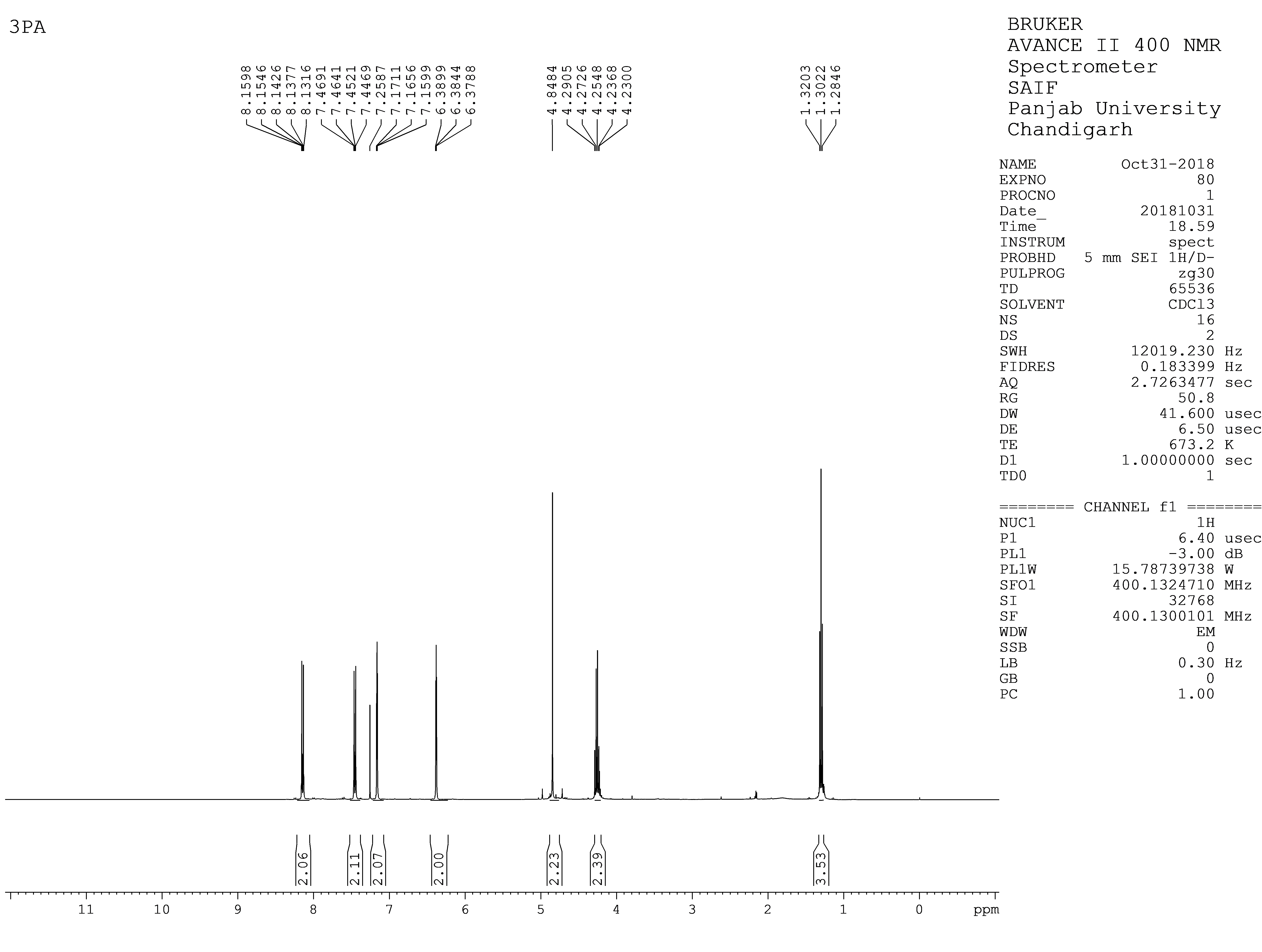

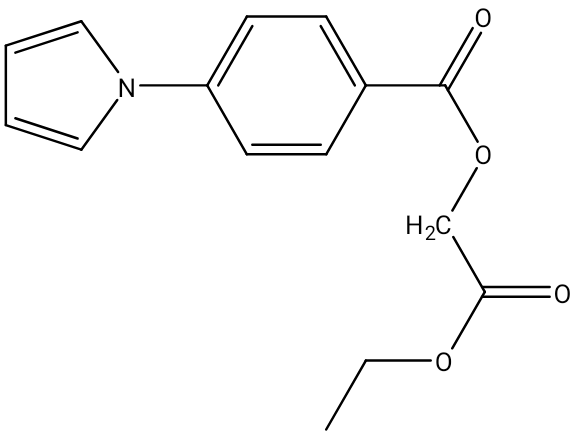


**Spectrum 4: IR Spectrum of compound 3**


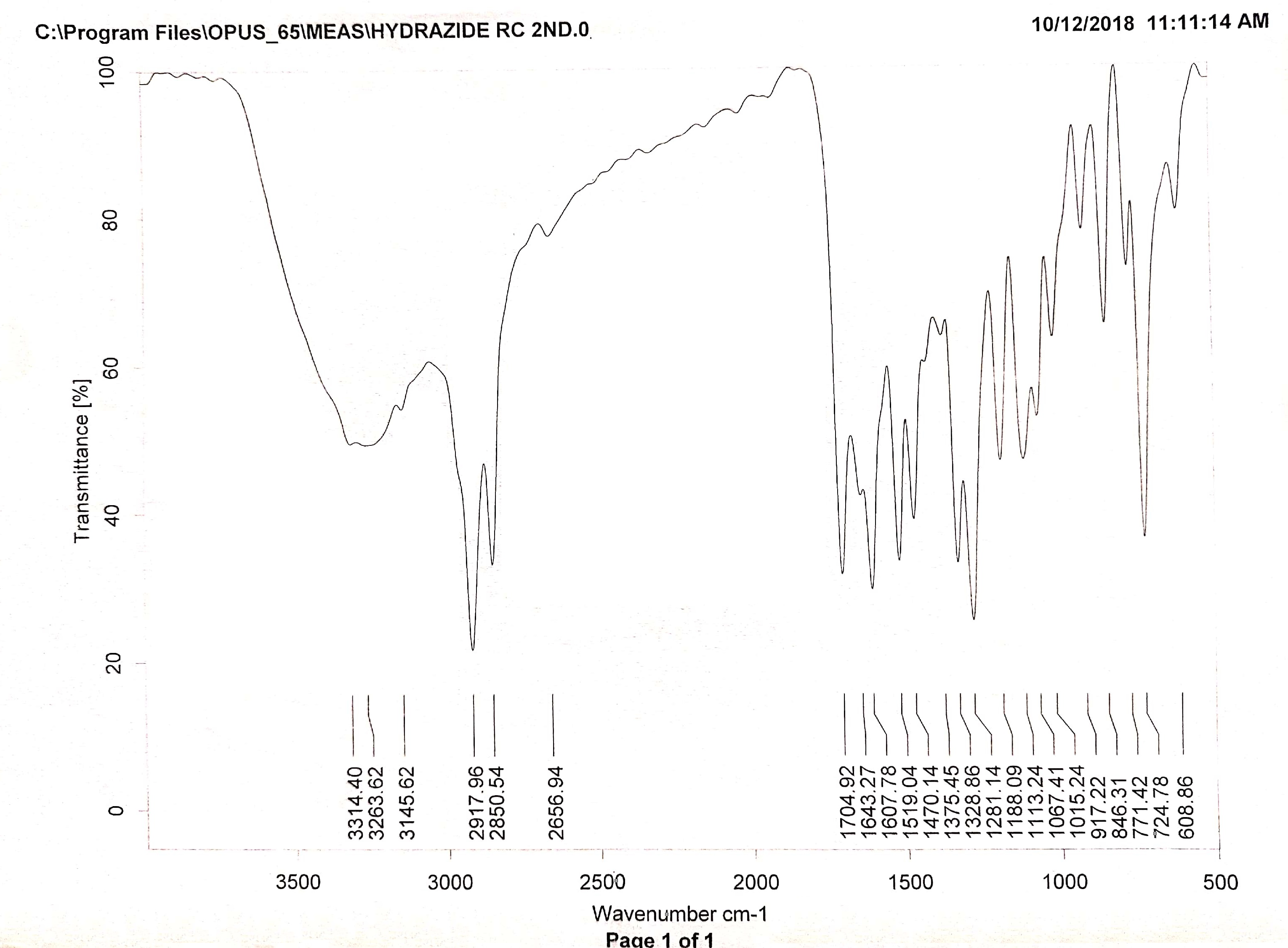

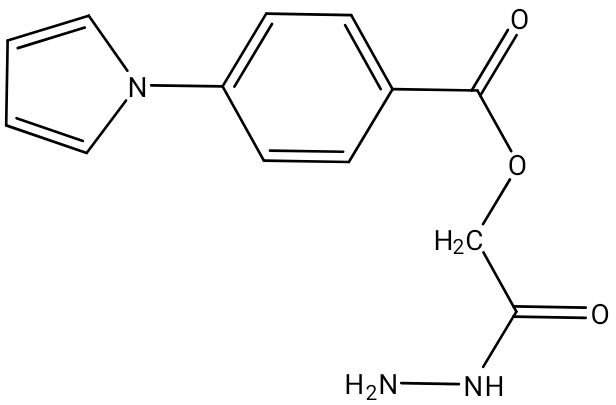


**Spectrum 5: ^1^H NMR Spectrum of compound 3**


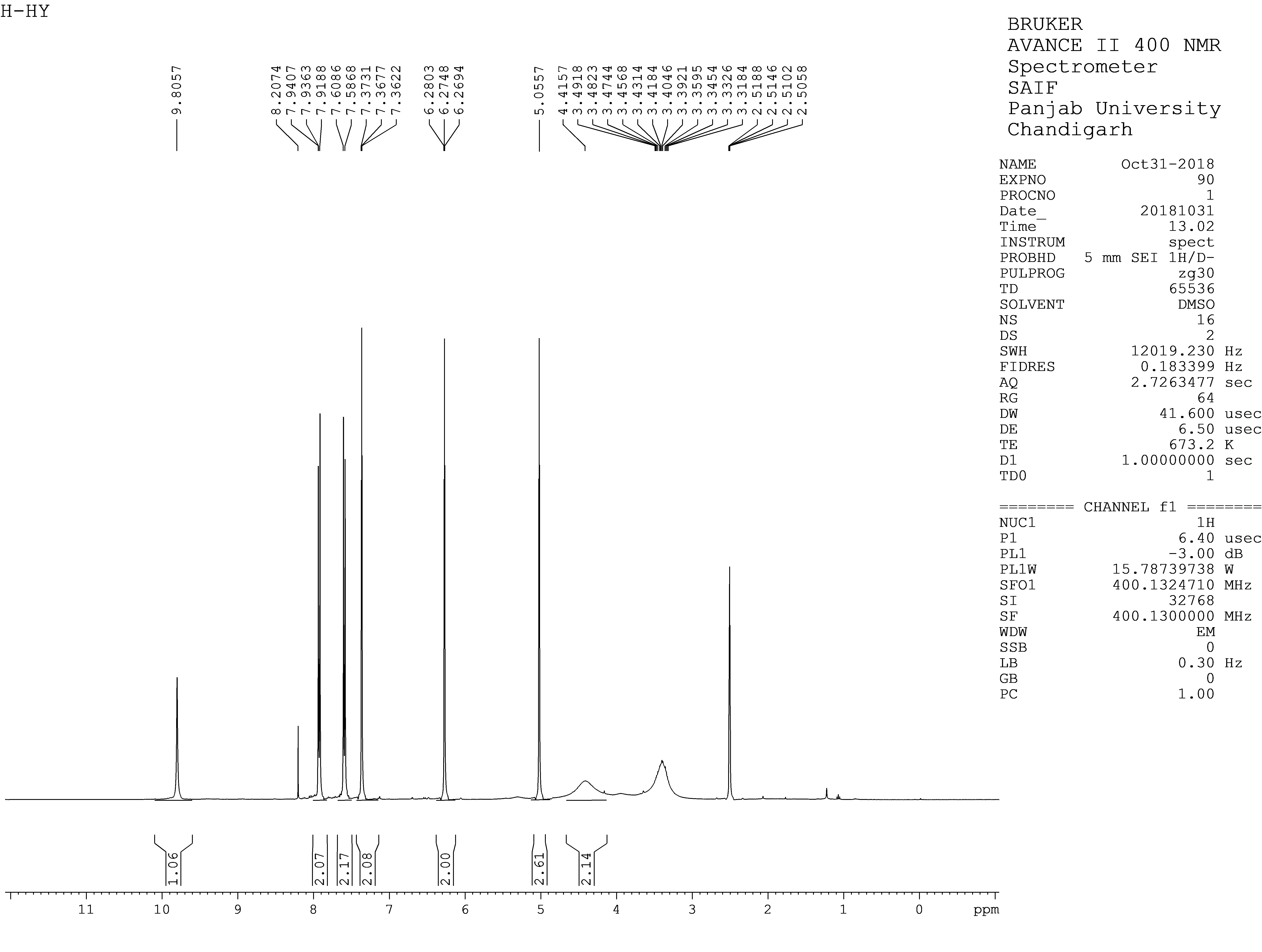

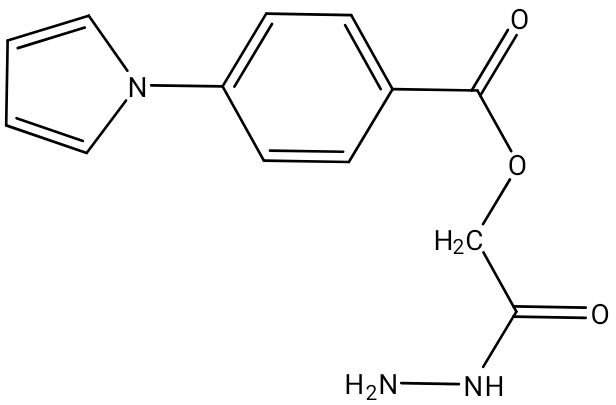


**Spectrum 6: Mass Spectrum of compound 3**


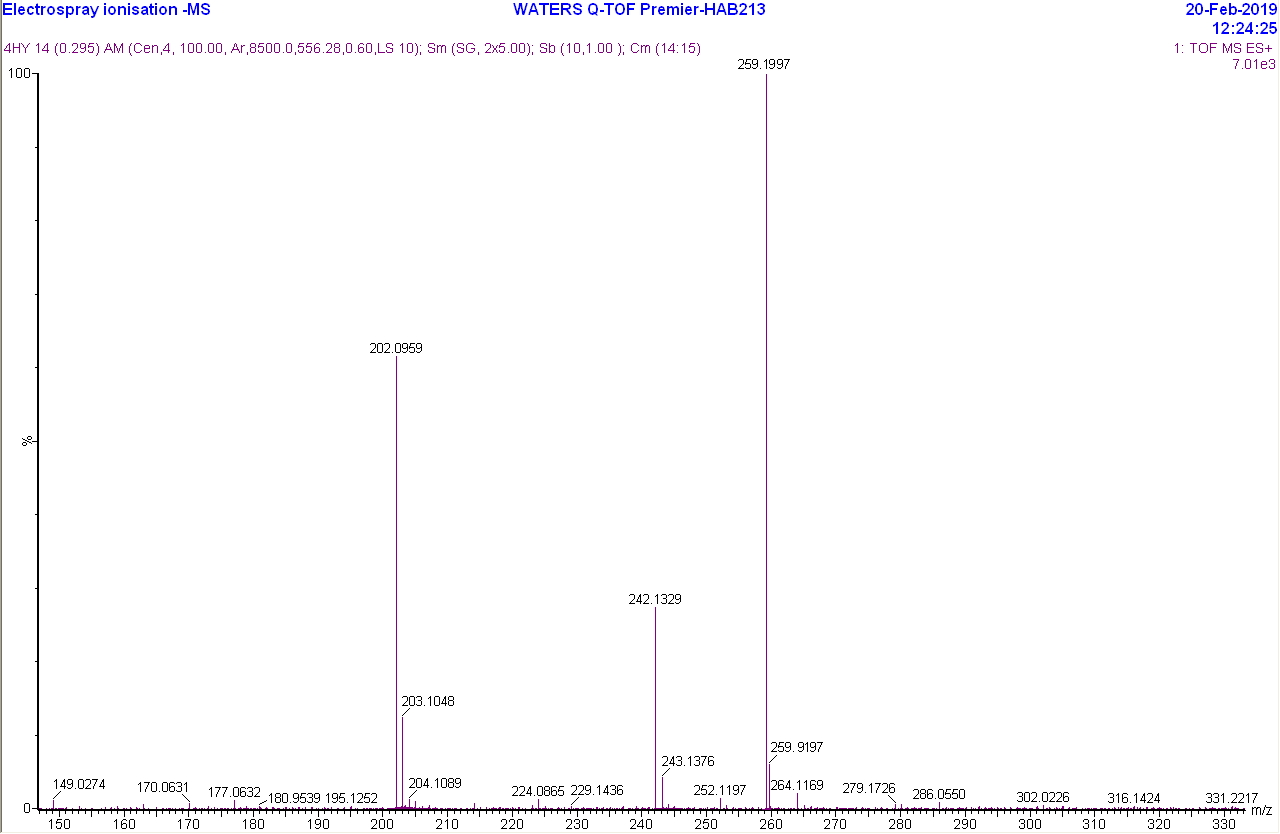

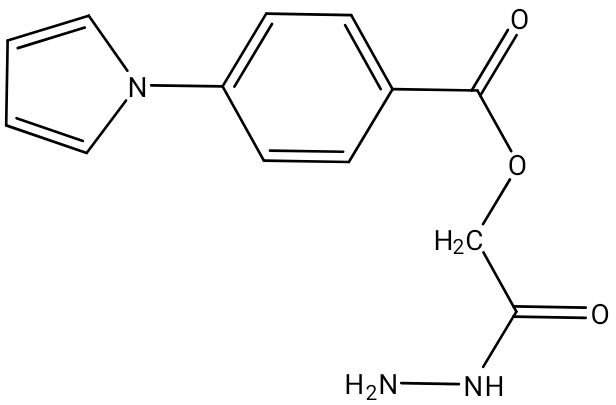


**Spectrum 7: IR Spectrum of compound 4a**


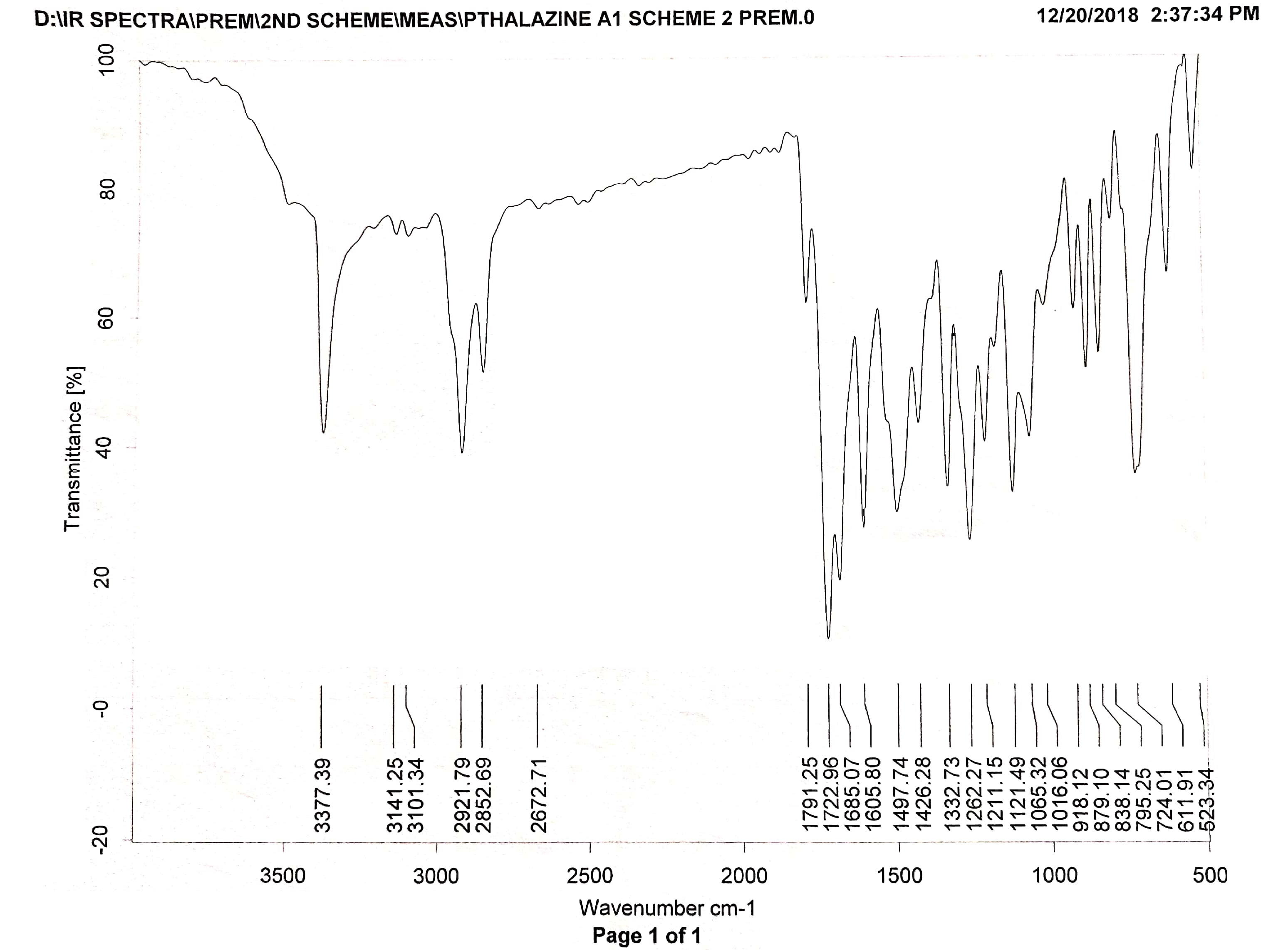

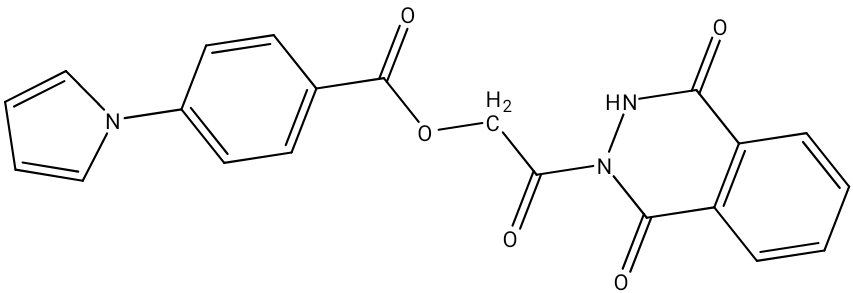


**Spectrum 8: ^1^H NMR Spectrum of compound 4a**


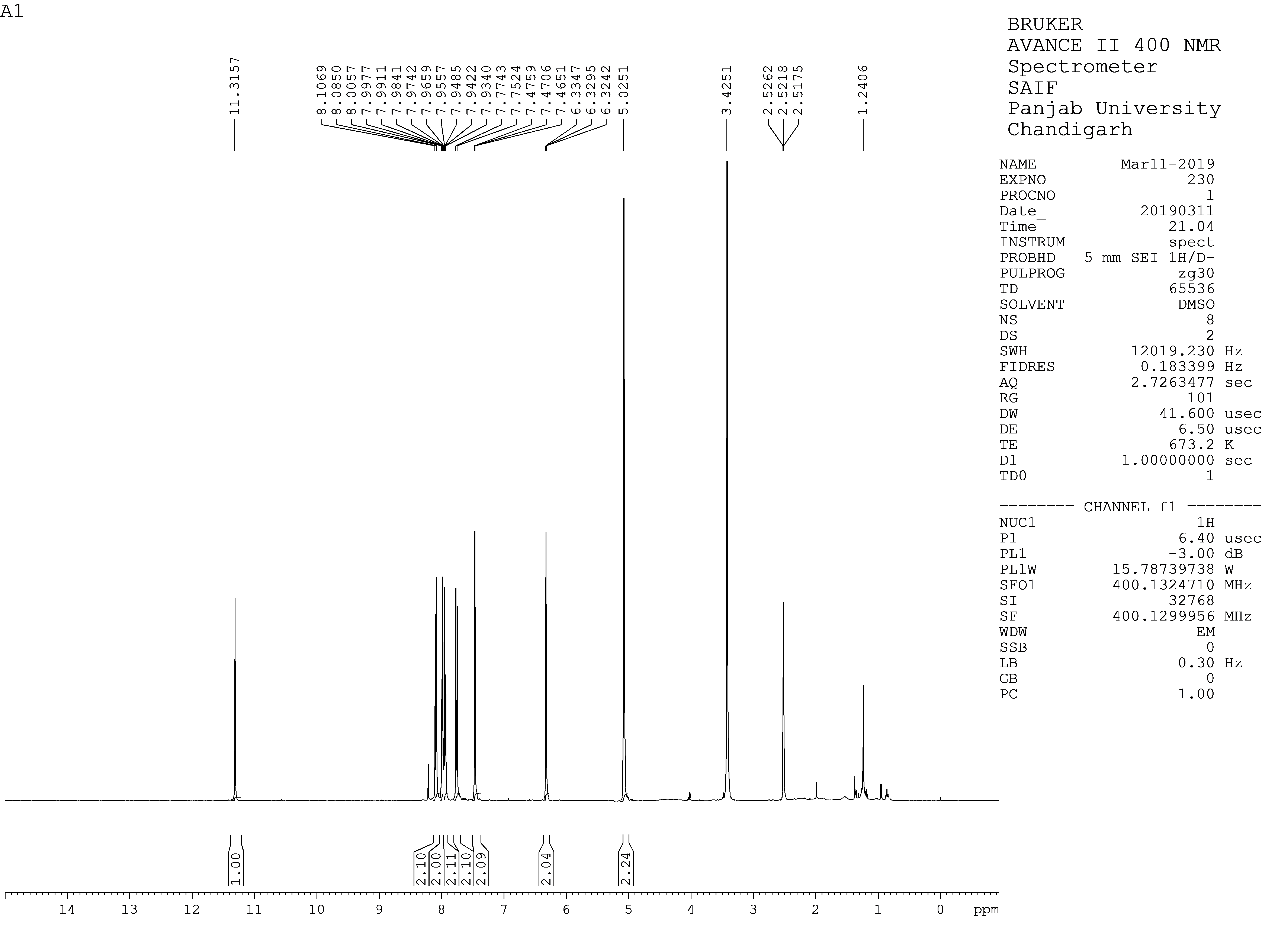

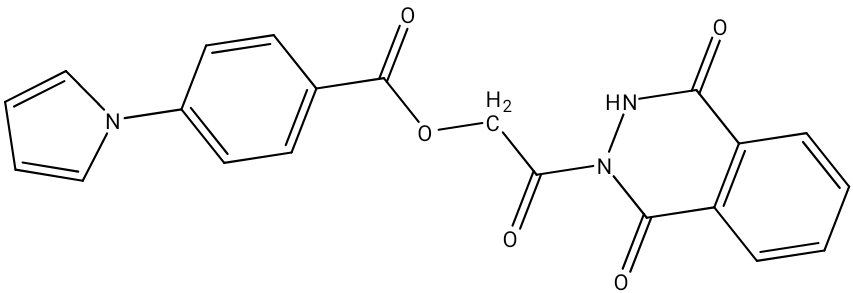


**Spectrum 9: ^13^C NMR Spectrum of compound 4a**


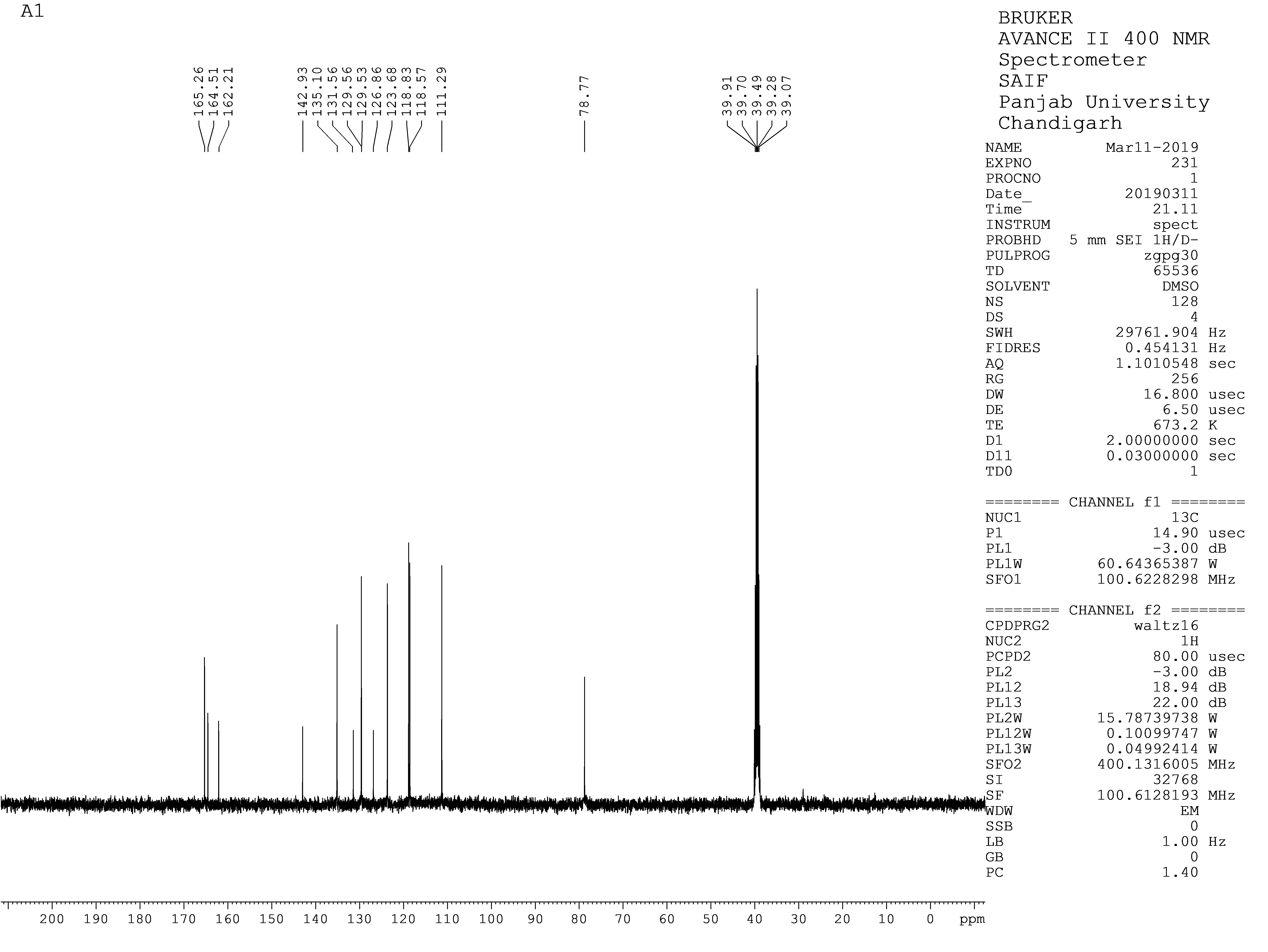

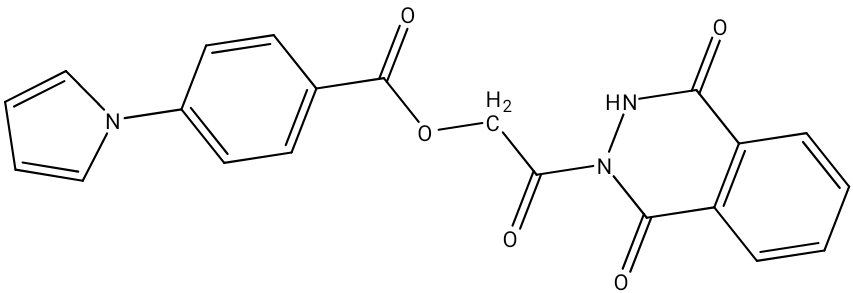


**Spectrum 10: Mass Spectrum of compound 4a**

**
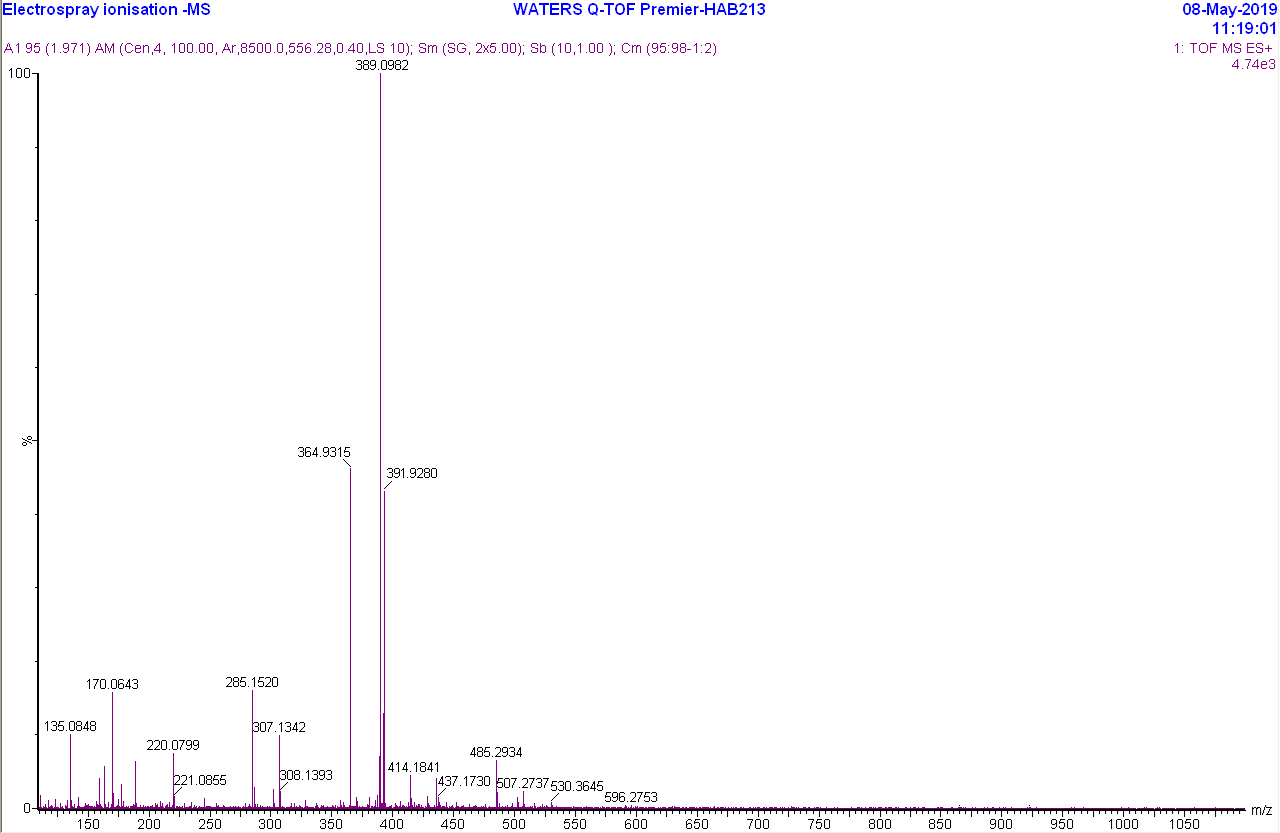
**
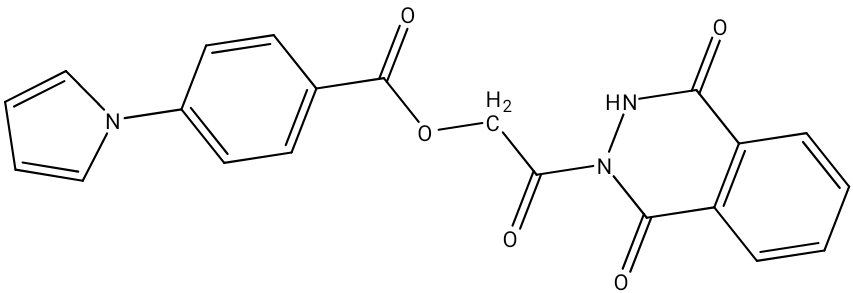


**Spectrum 11: IR Spectrum of compound 4b**


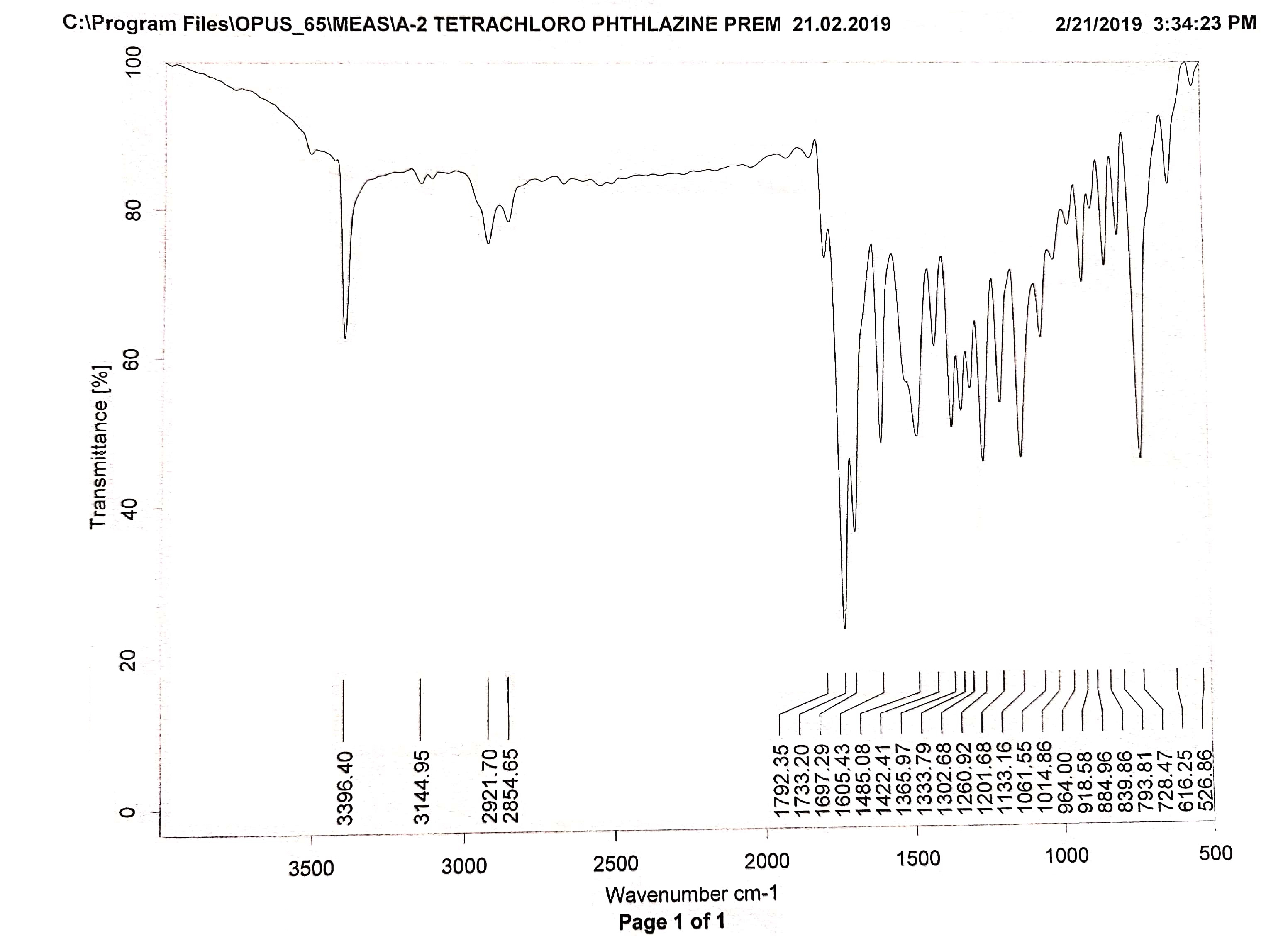

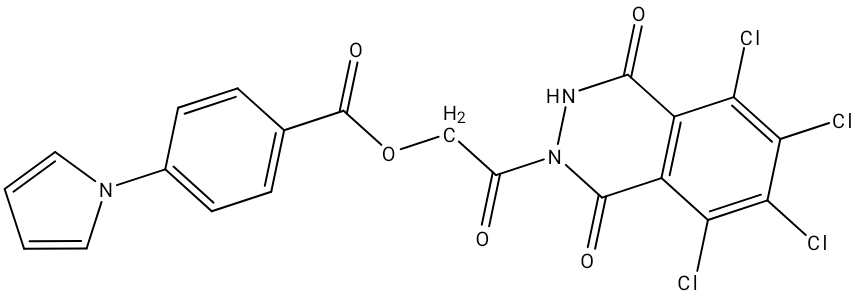


**Spectrum 12: ^1^H NMR Spectrum of compound 4b**


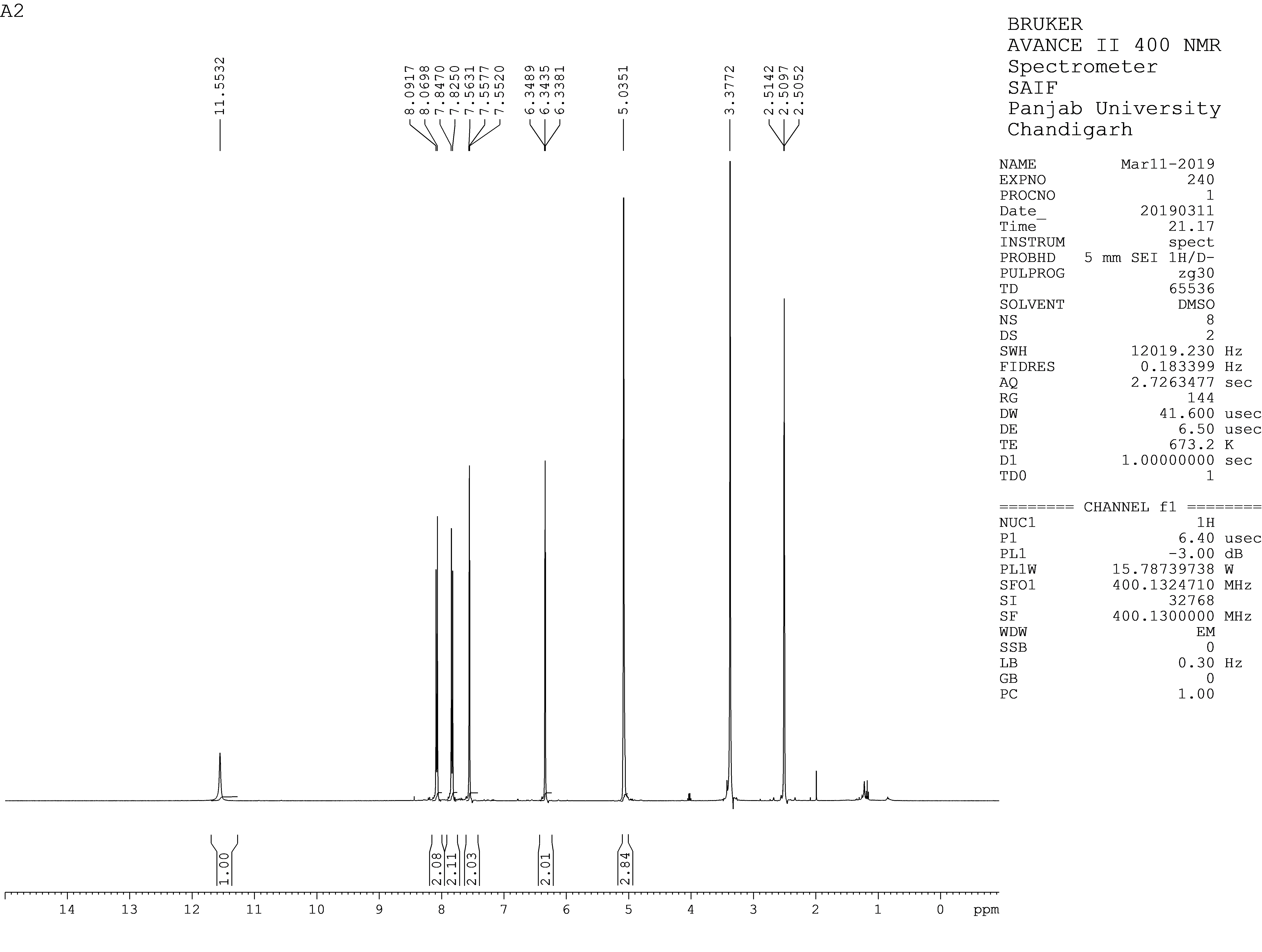

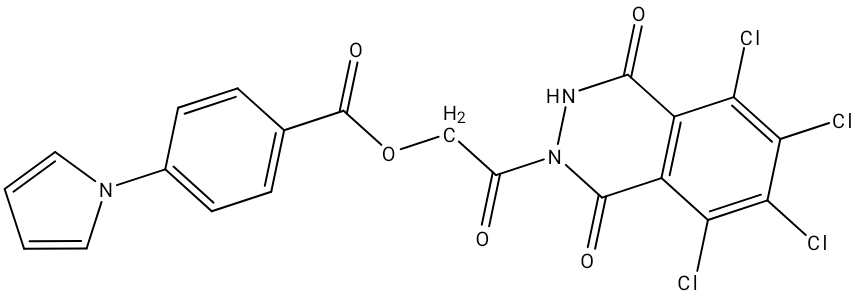


**Spectrum 13: ^13^C NMR Spectrum of compound 4b**


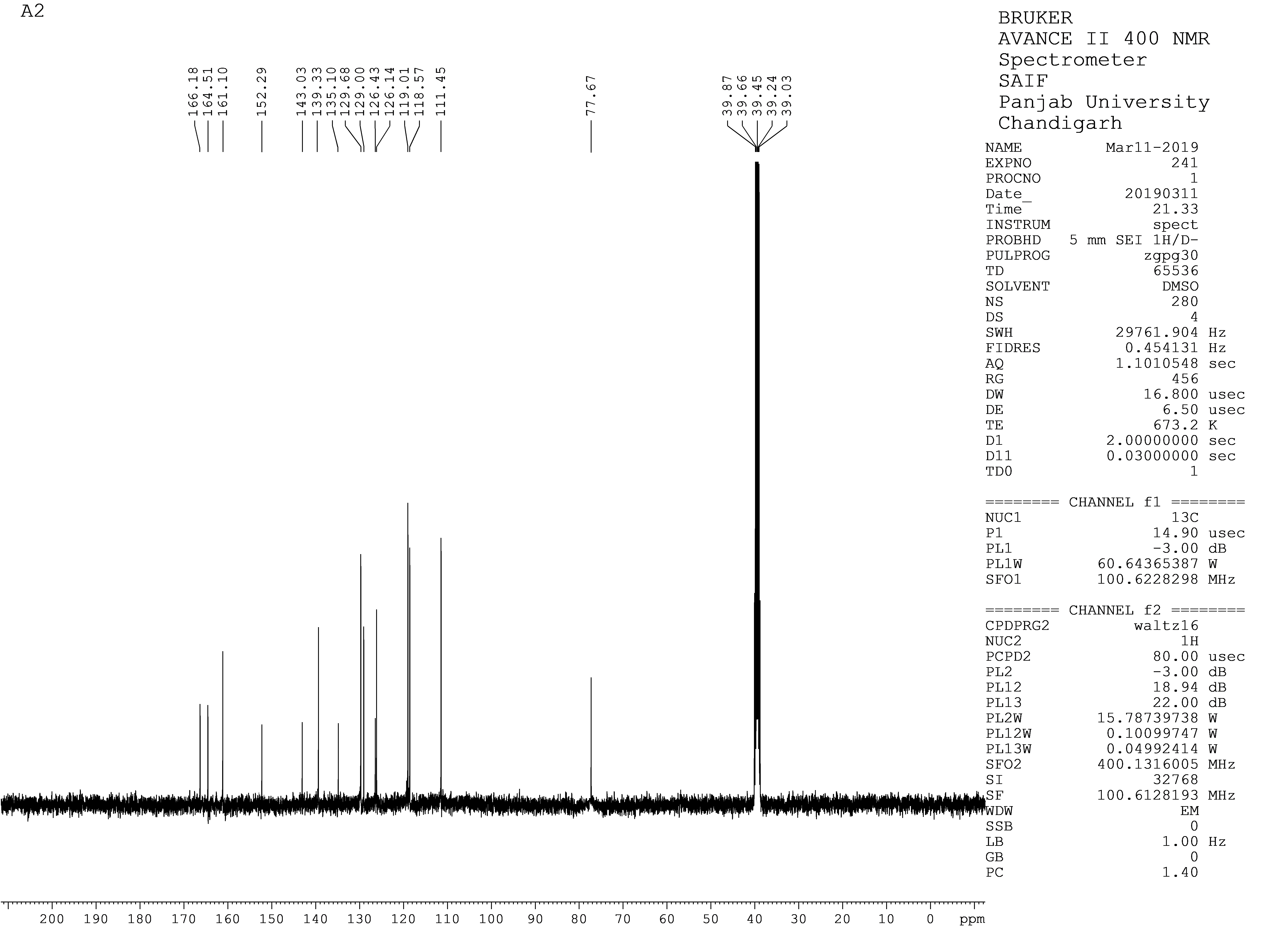

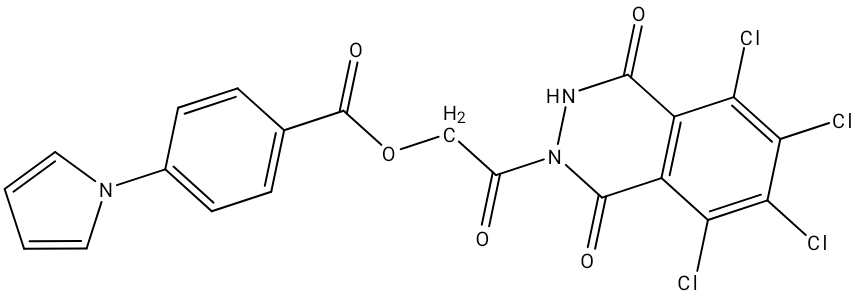


**Spectrum 14: Mass Spectrum of compound 4b**


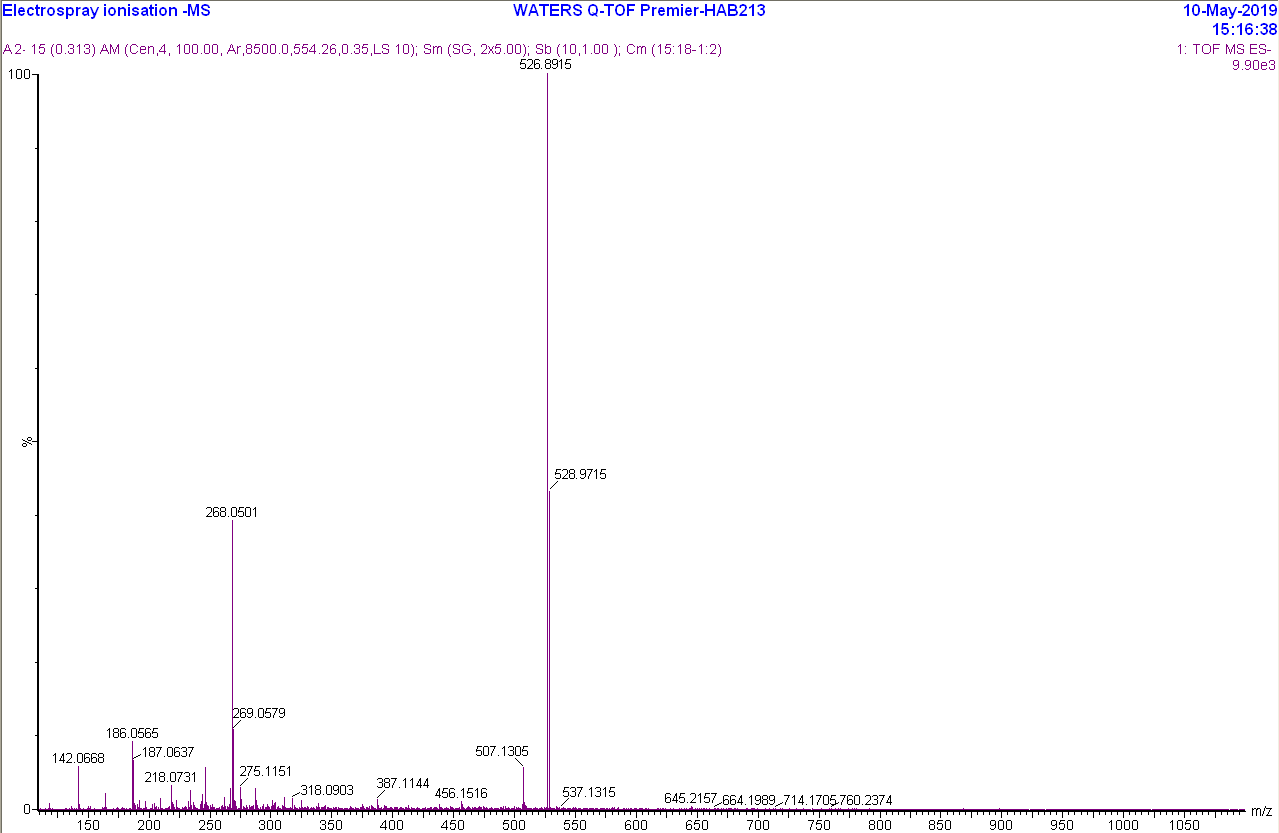

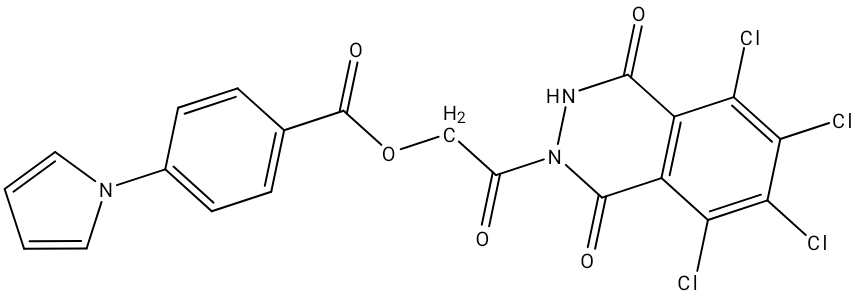


**Spectrum 15: IR Spectrum of compound 4c**


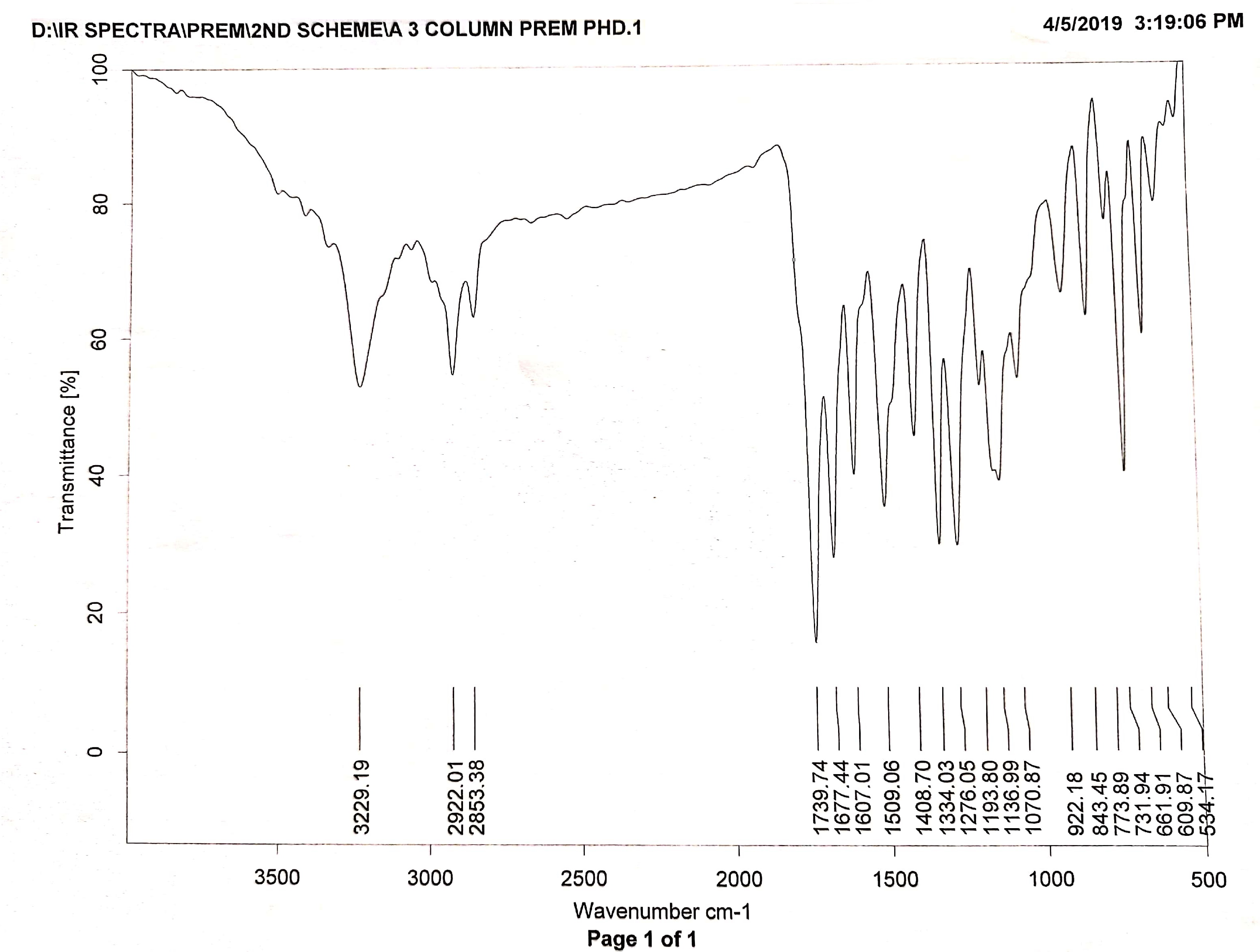

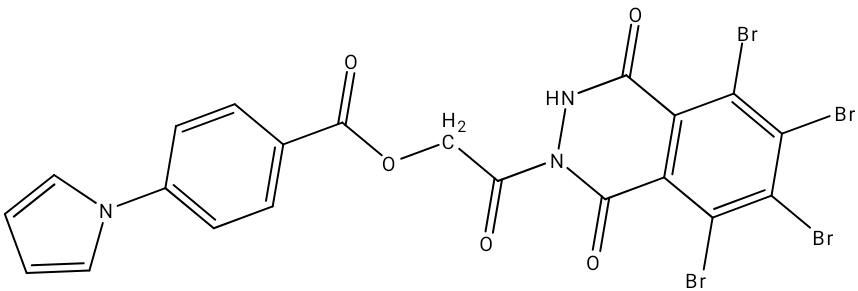


**Spectrum 16: ^1^H NMR Spectrum of compound 4c**


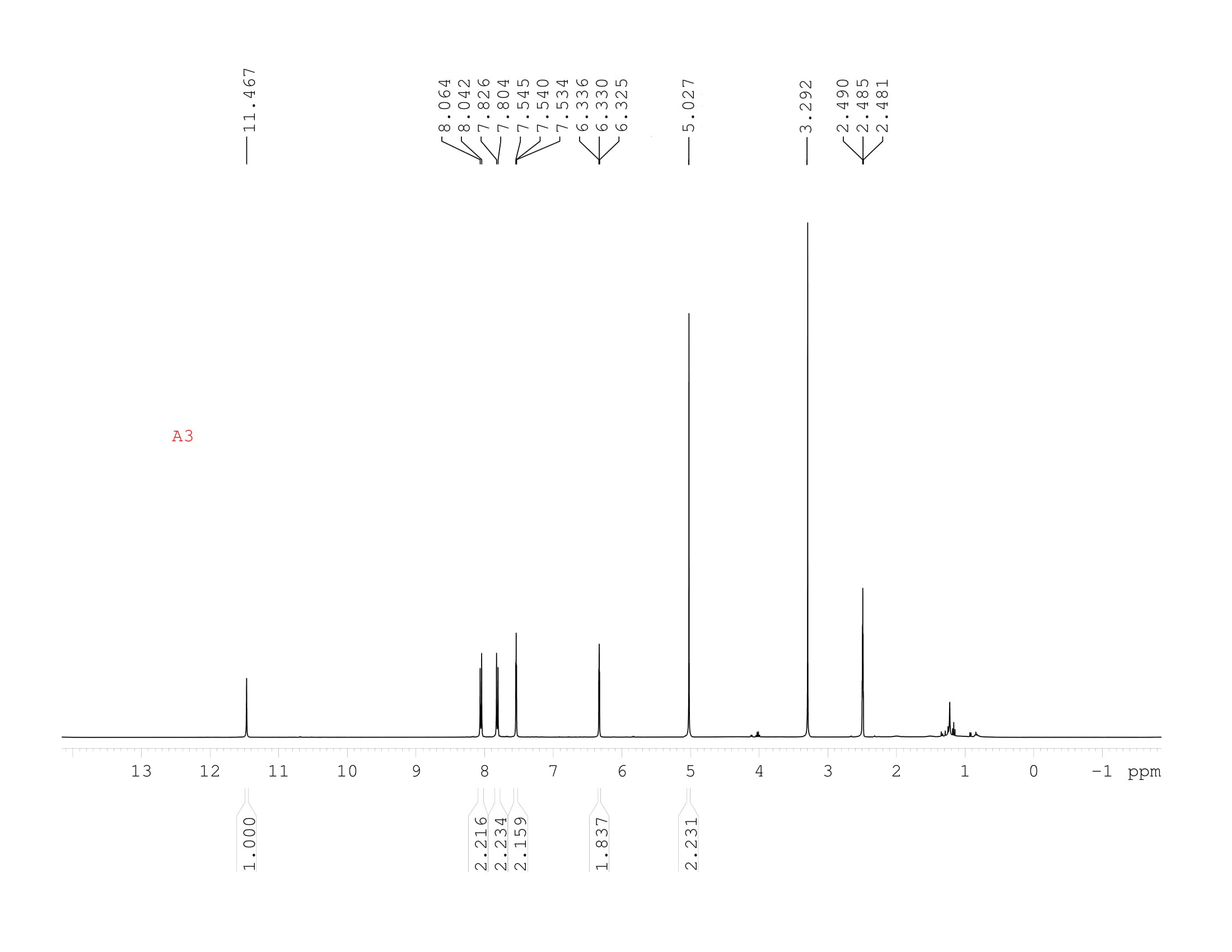

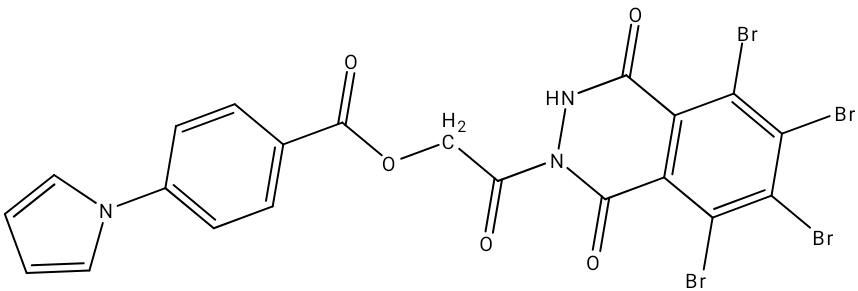


**Spectrum 17: ^13^C NMR Spectrum of compound 4c**


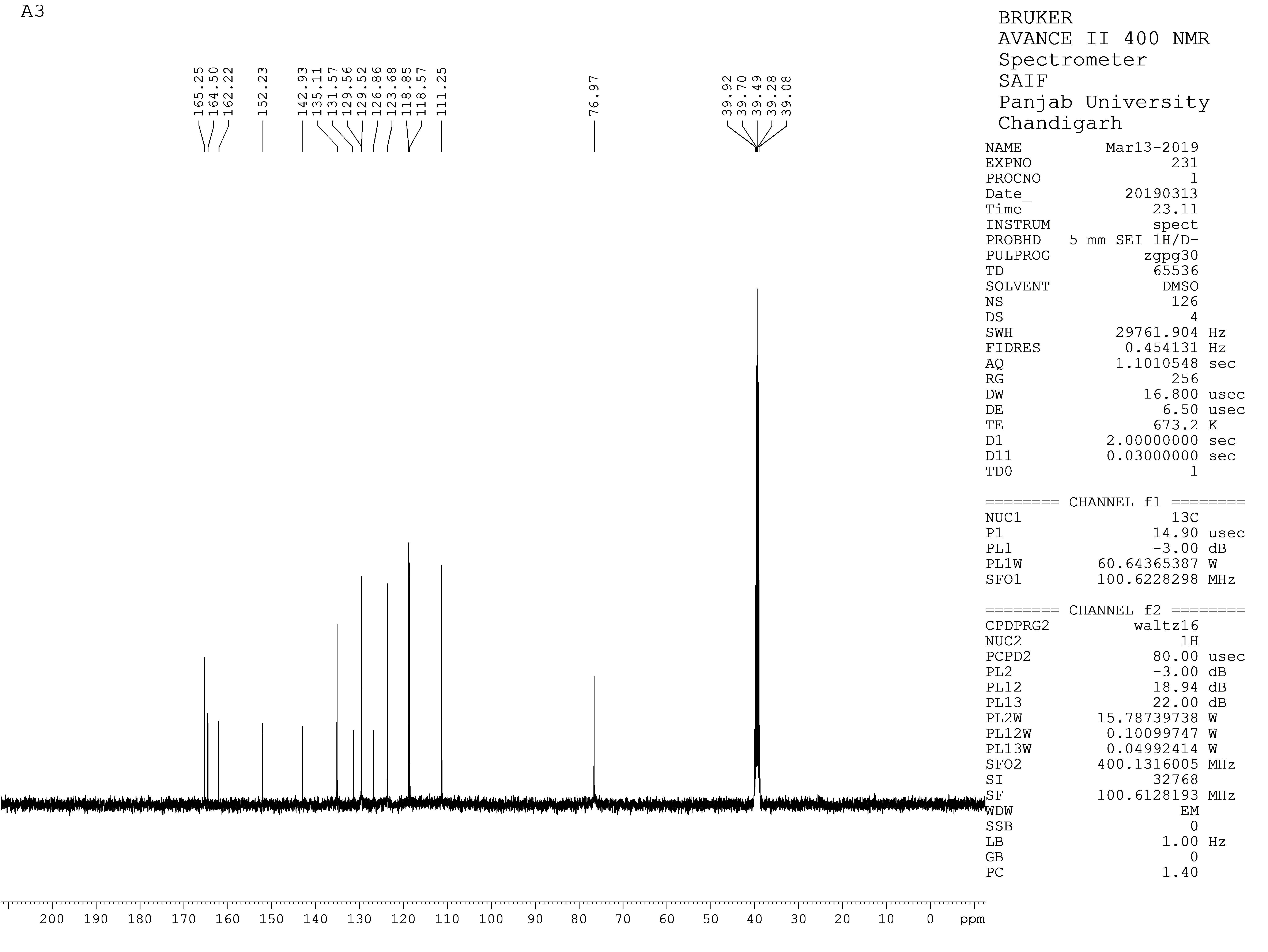

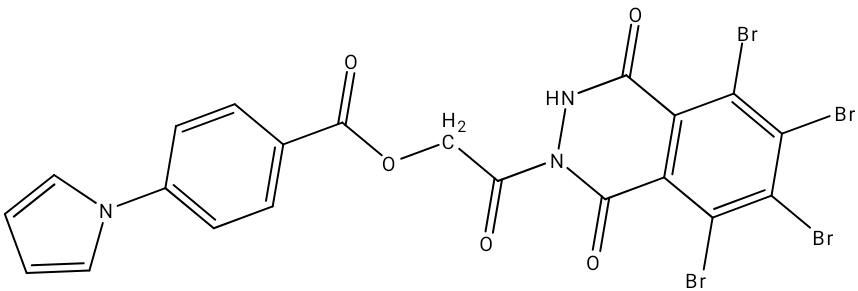


**Spectrum 18: Mass Spectrum of compound 4c**

**
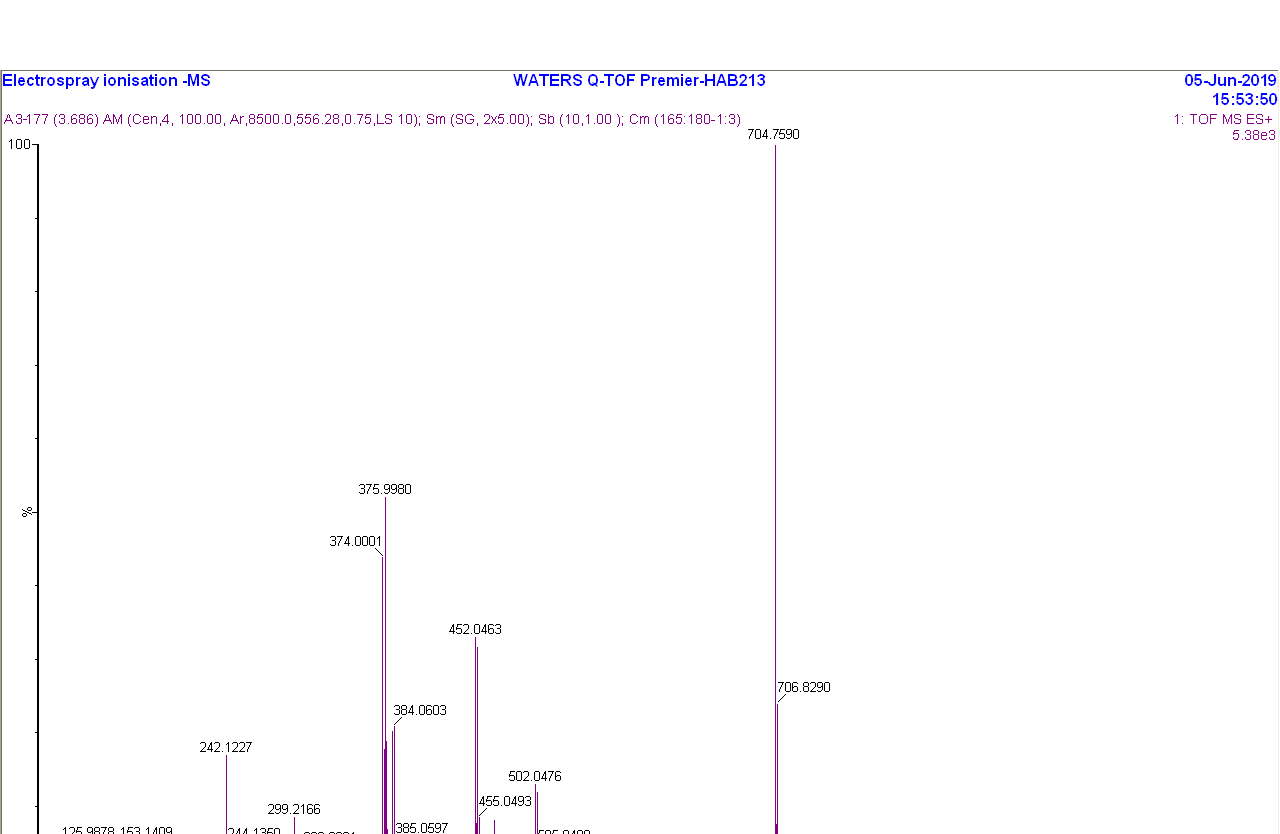
**
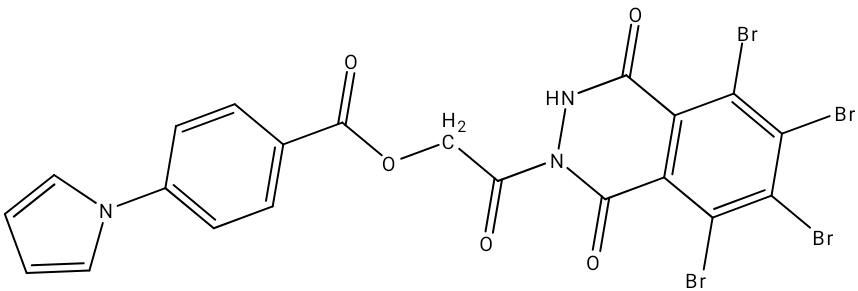


**Spectrum 19: IR Spectrum of compound 4d**


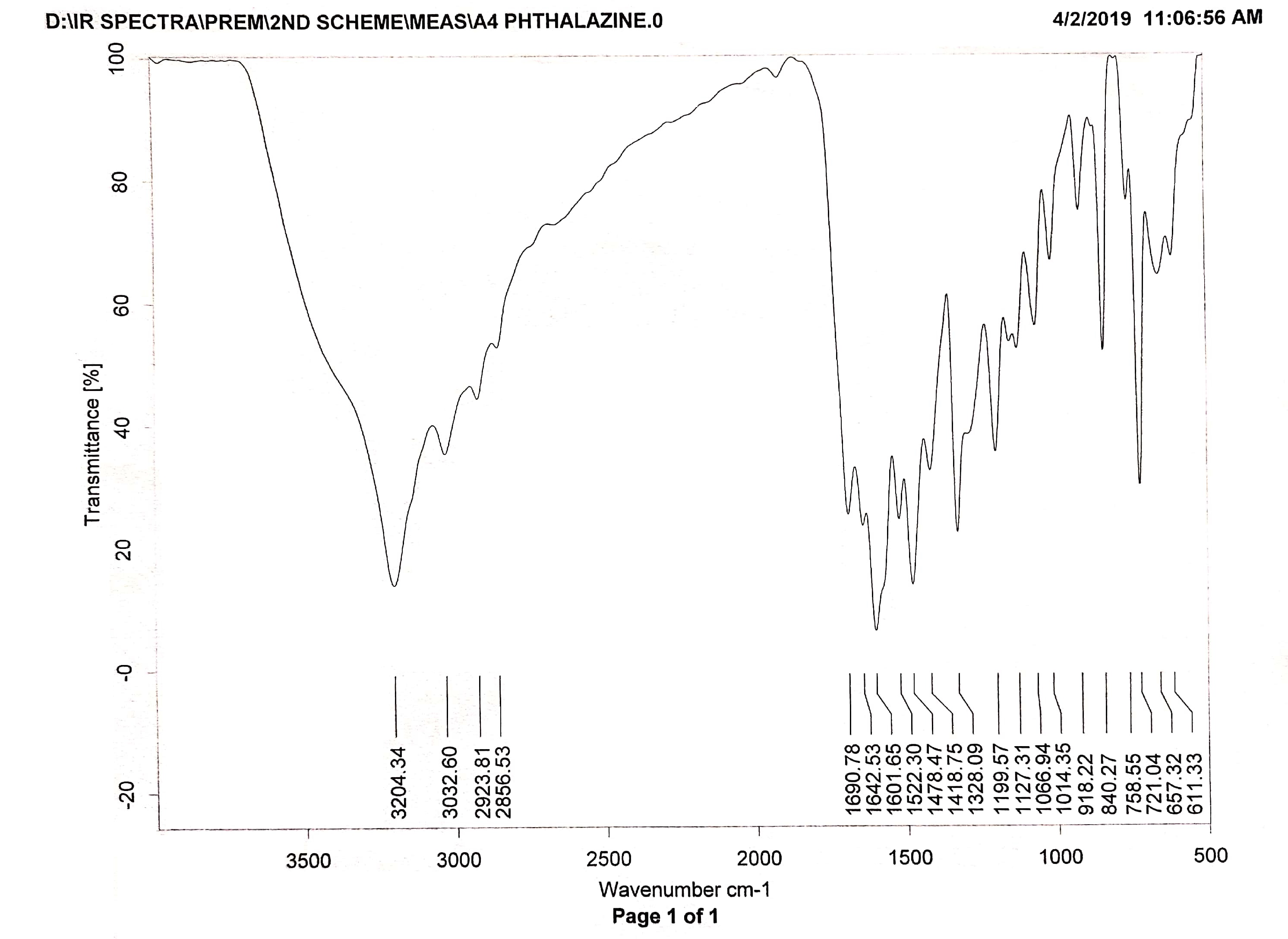

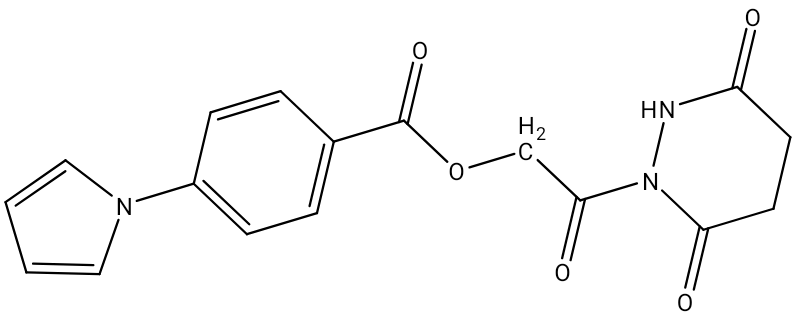


**Spectrum 20: ^1^H NMR Spectrum of compound 4d**


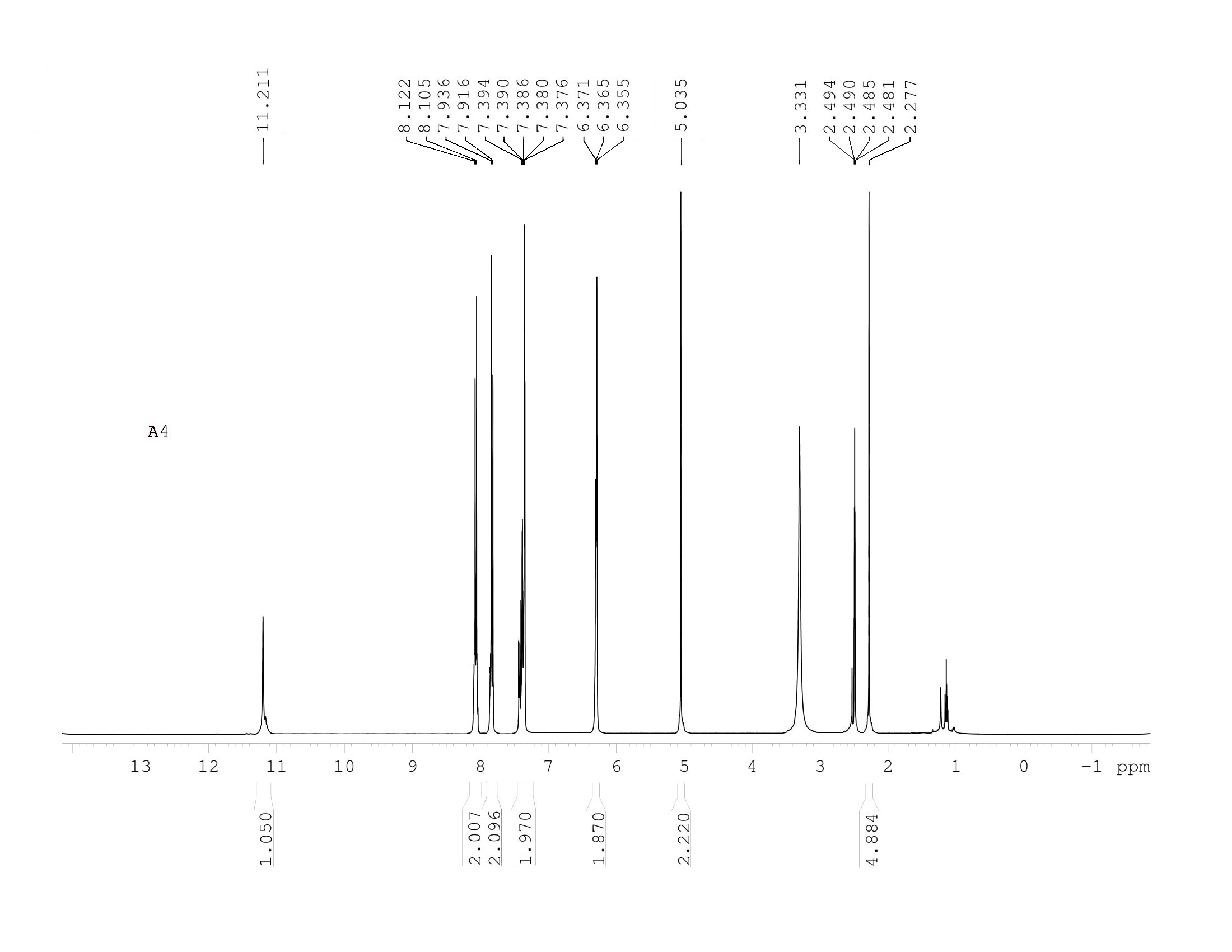

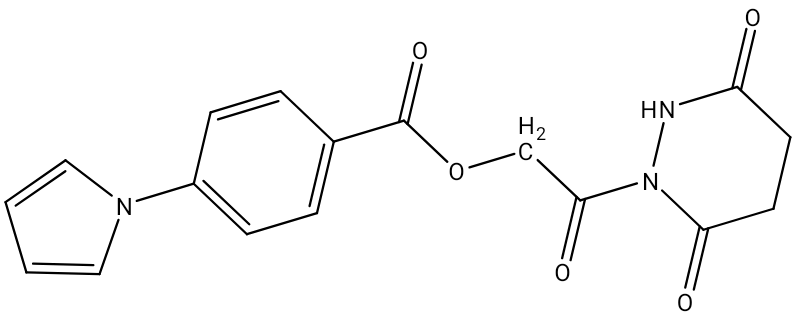


**Spectrum 21: ^13^C NMR Spectrum of compound 4d**


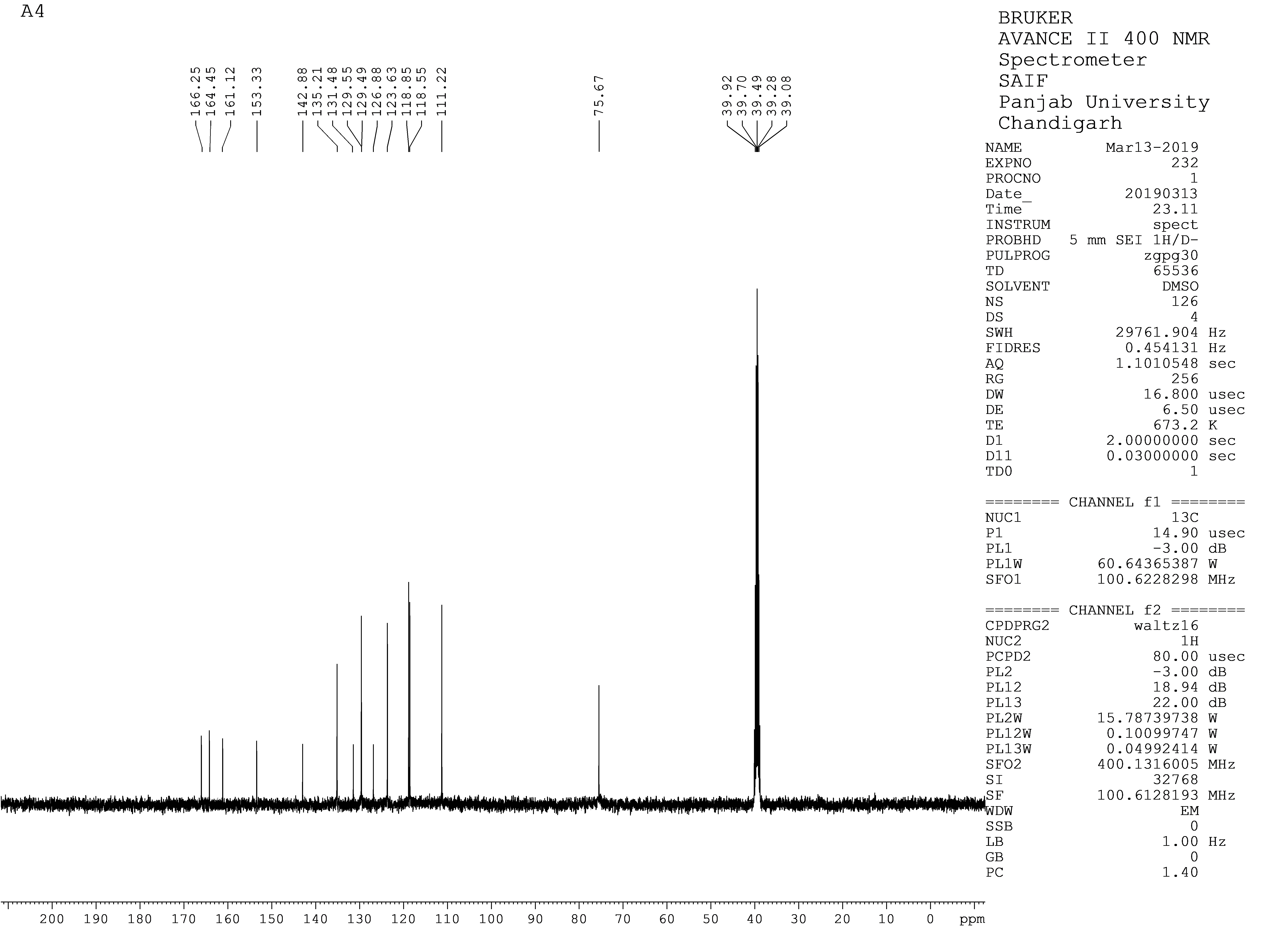

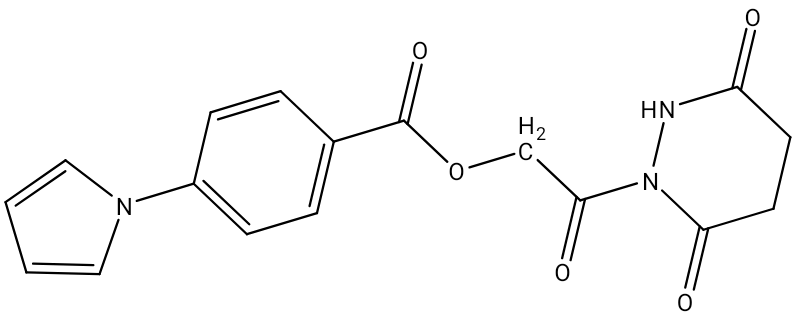


**Spectrum 22: Mass Spectrum of compound 4d**

**
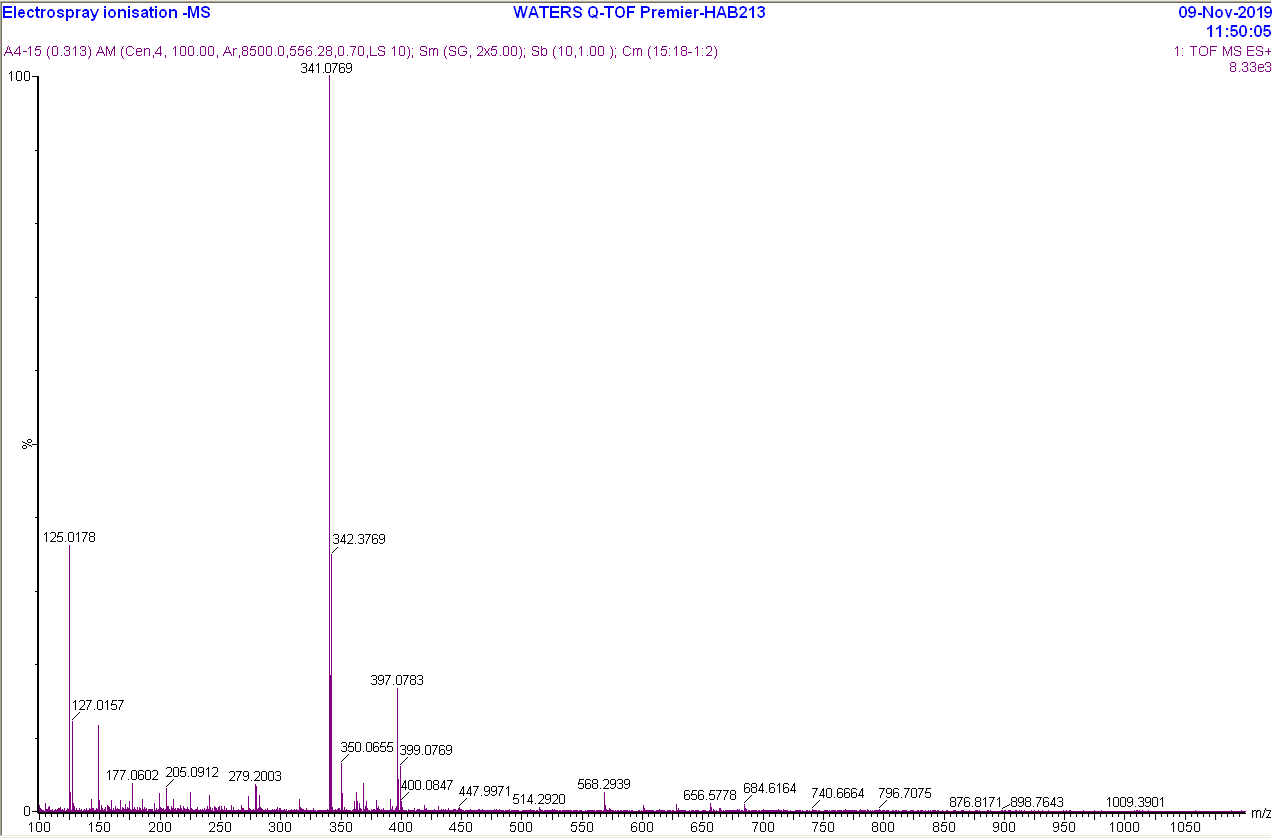
**
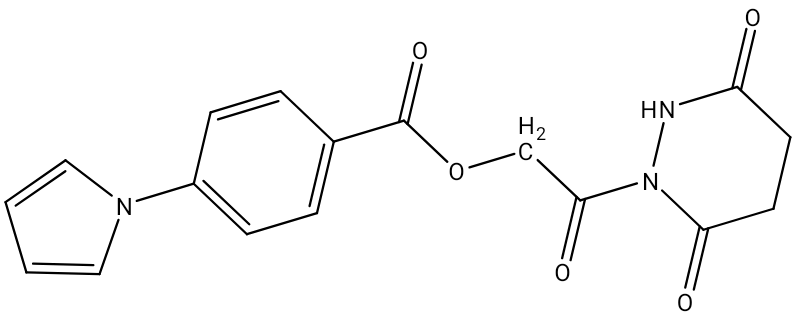


**Spectrum 23: IR Spectrum of compound 4e**


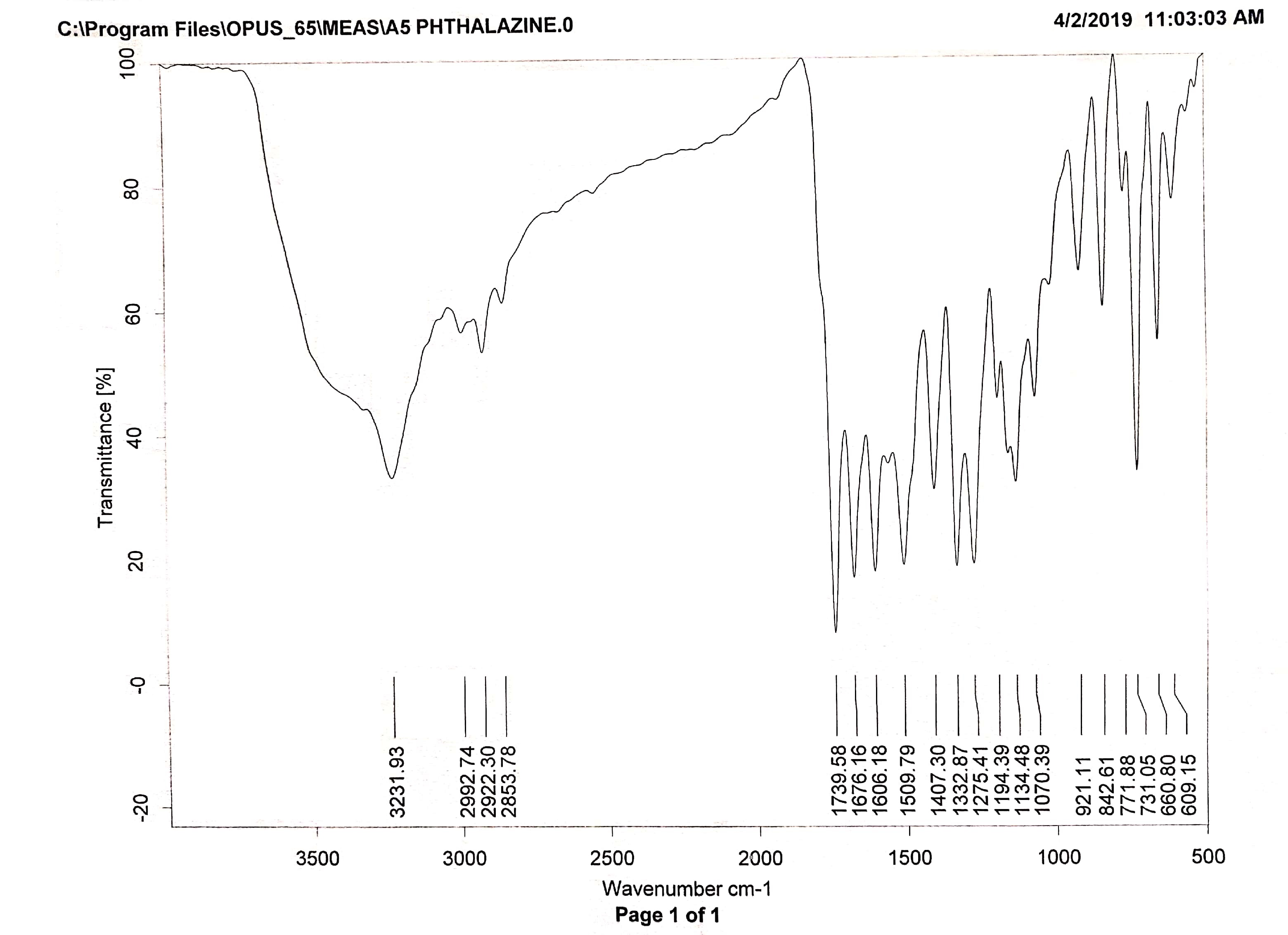

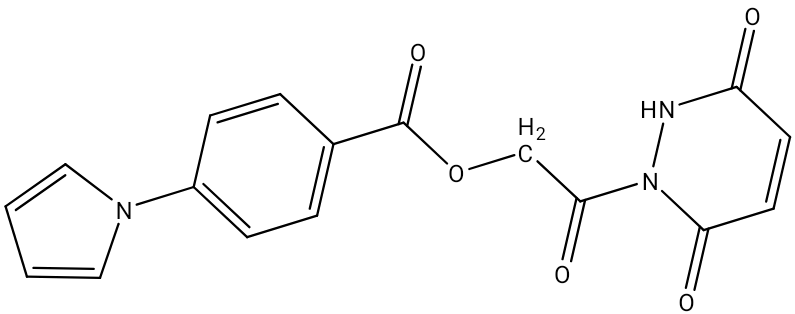


**Spectrum 24: ^1^H NMR Spectrum of compound 4e**


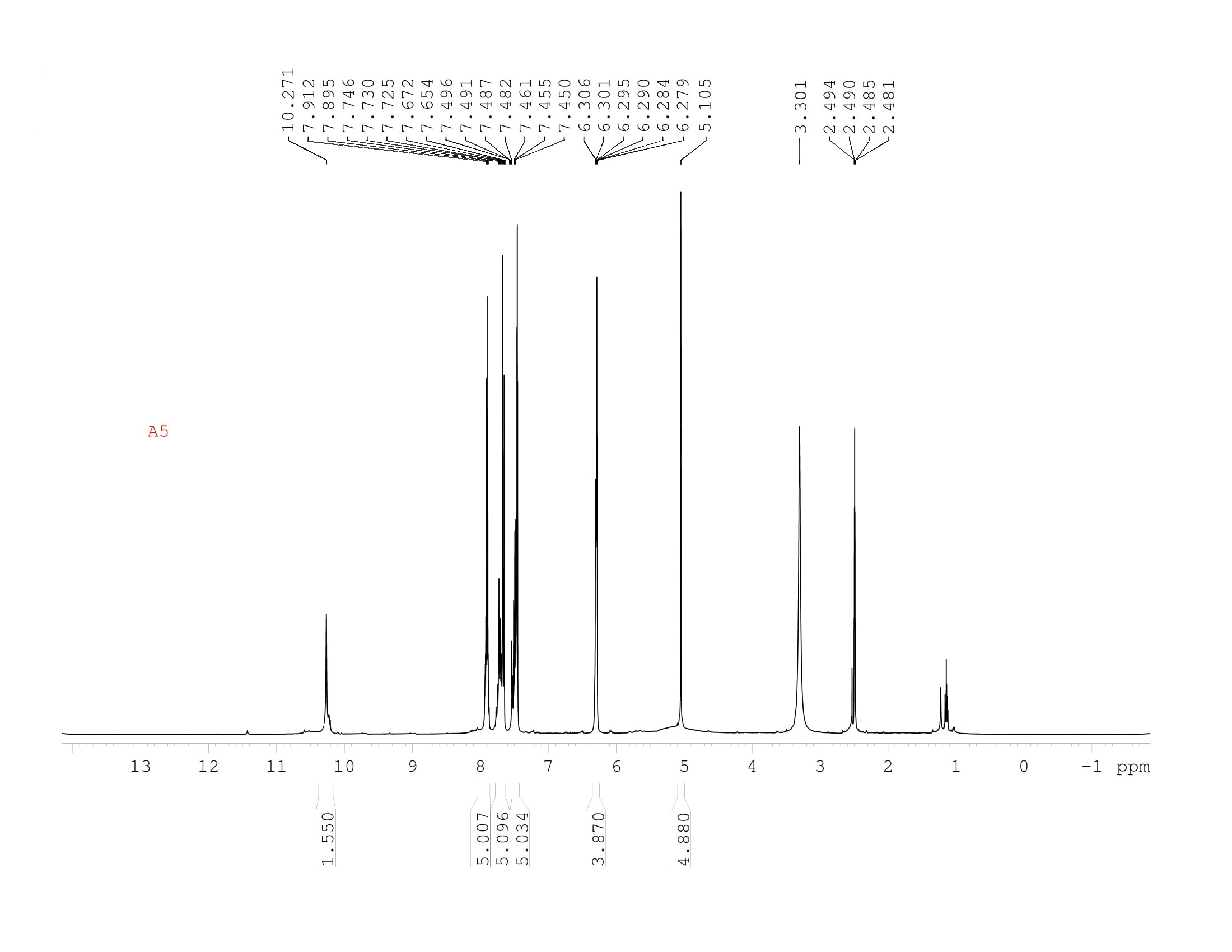

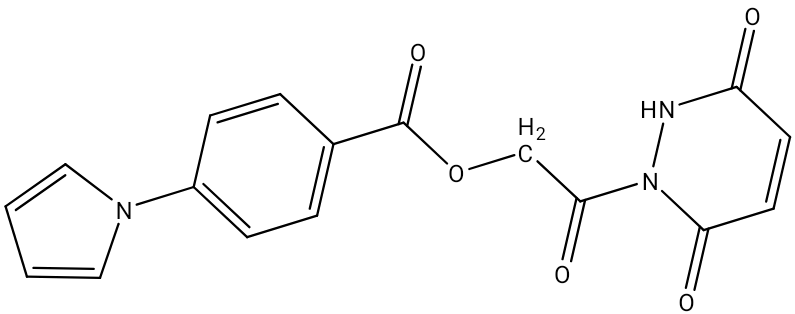


**Spectrum 25: ^13^C NMR Spectrum of compound 4e**
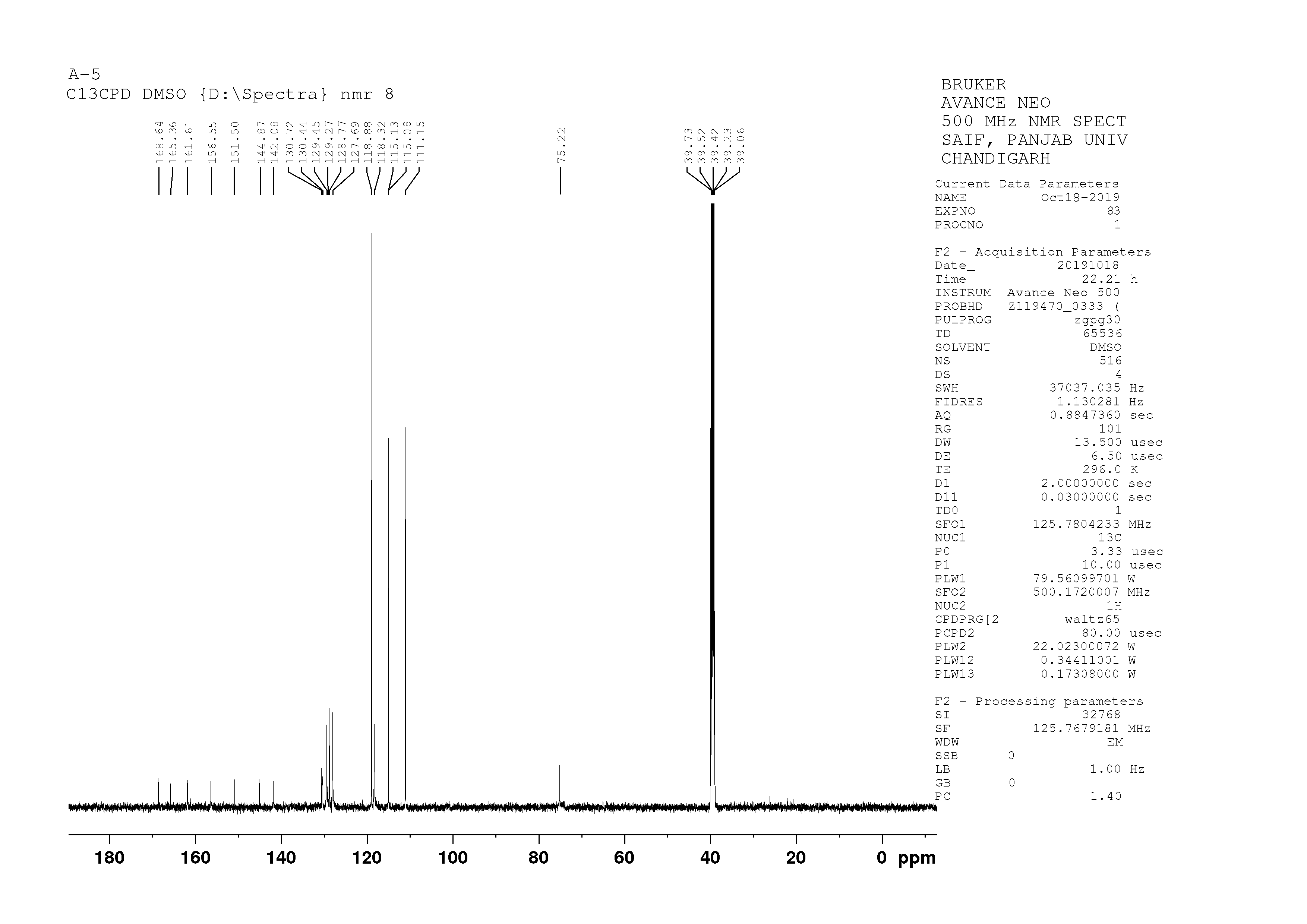

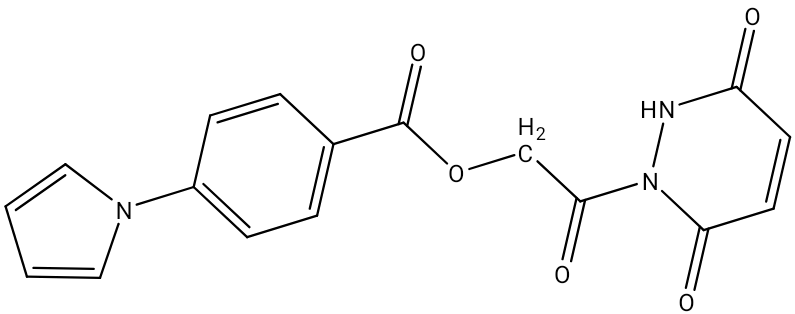


**Spectrum 26: Mass Spectrum of compound 4e**

**
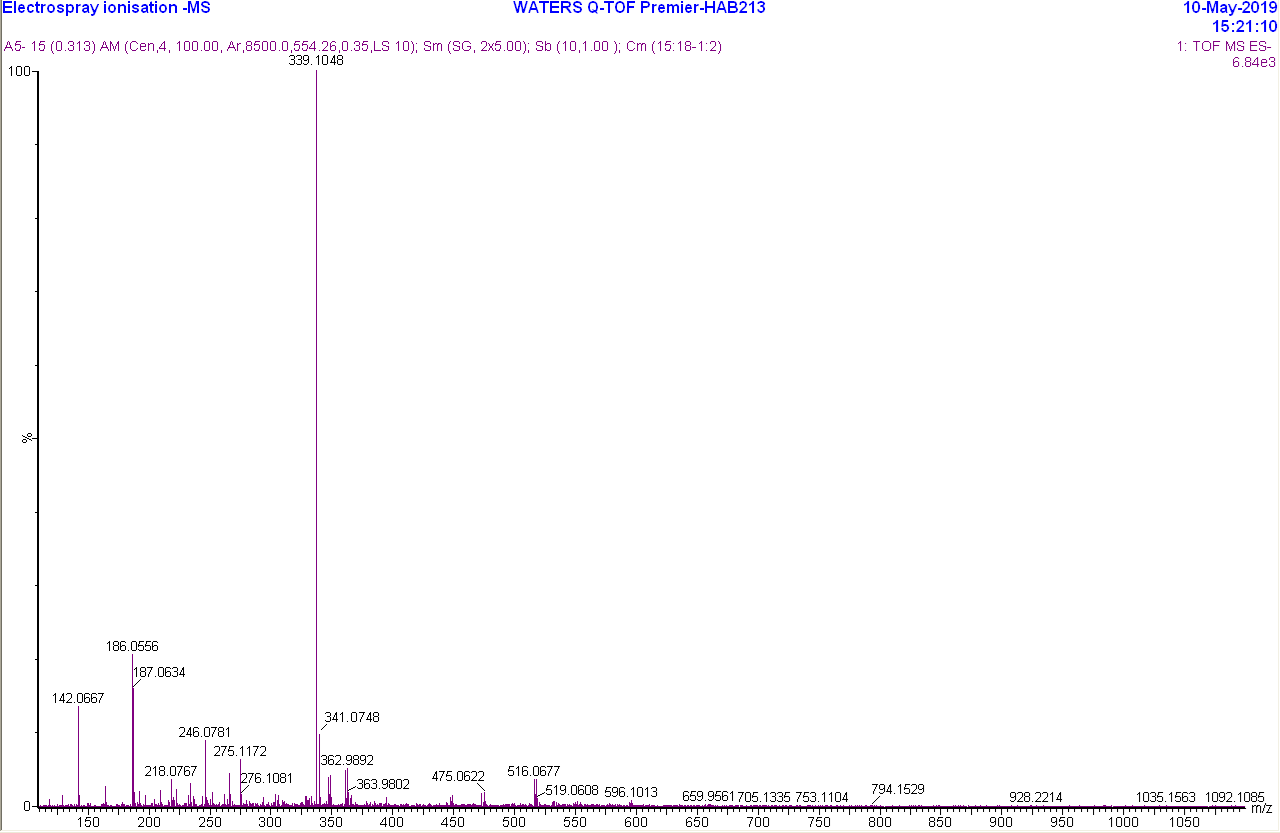
**
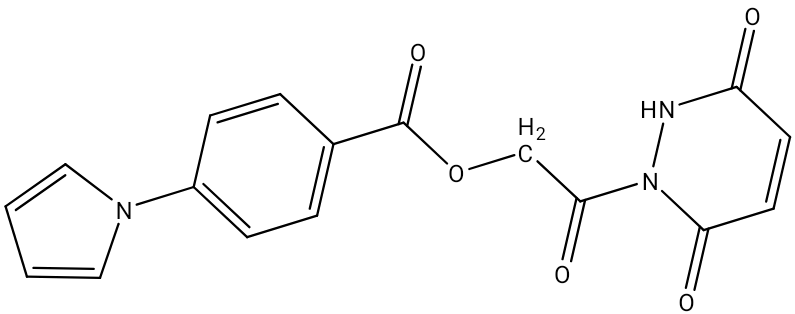


**Spectrum 27: IR Spectrum of compound 4f**


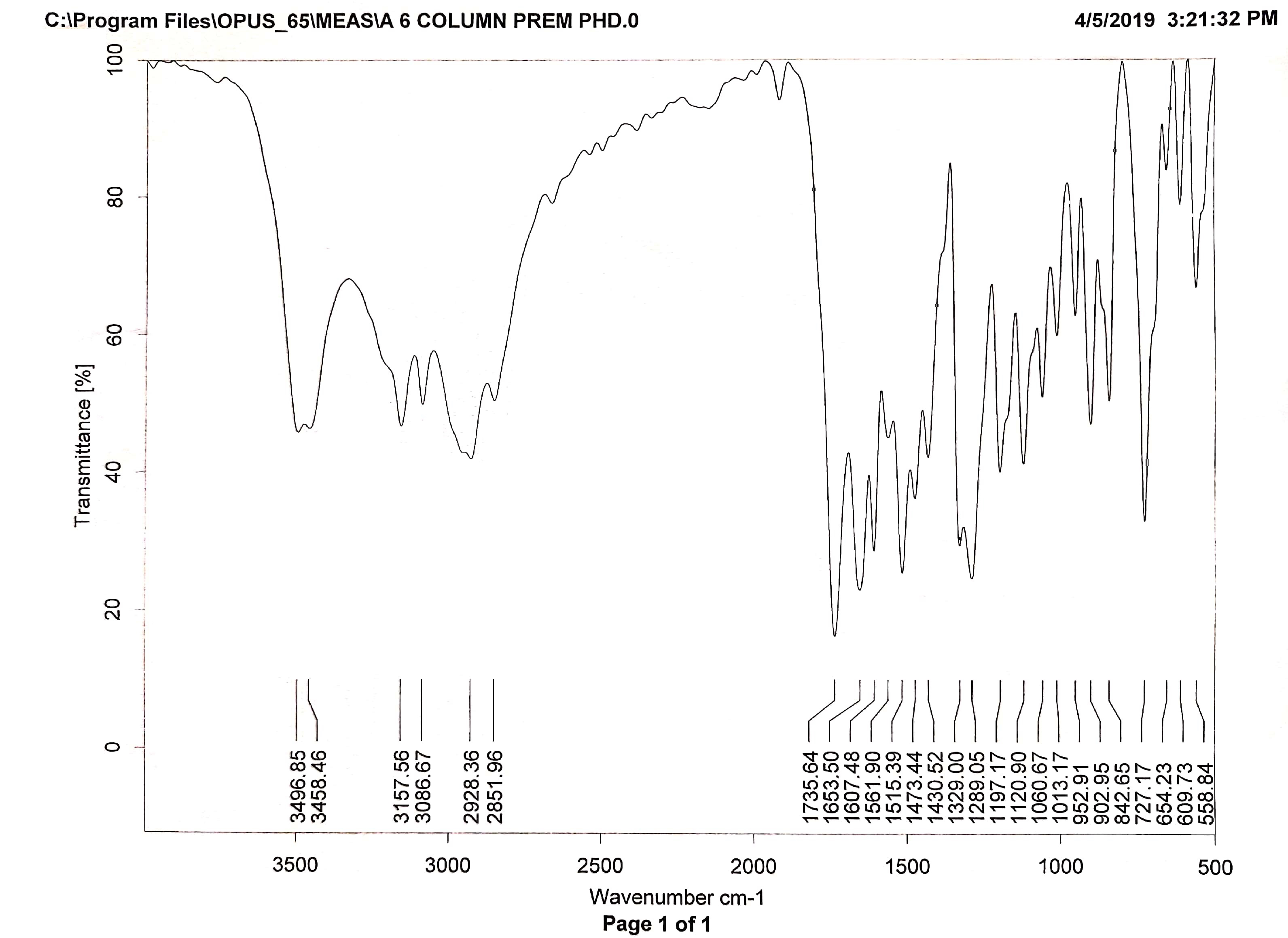

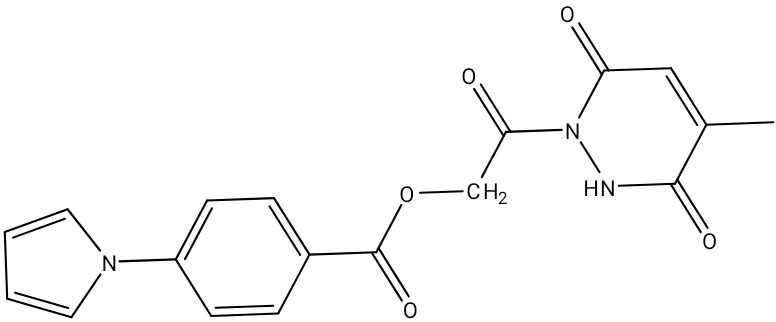


**Spectrum 28: ^1^H NMR Spectrum of compound 4f**


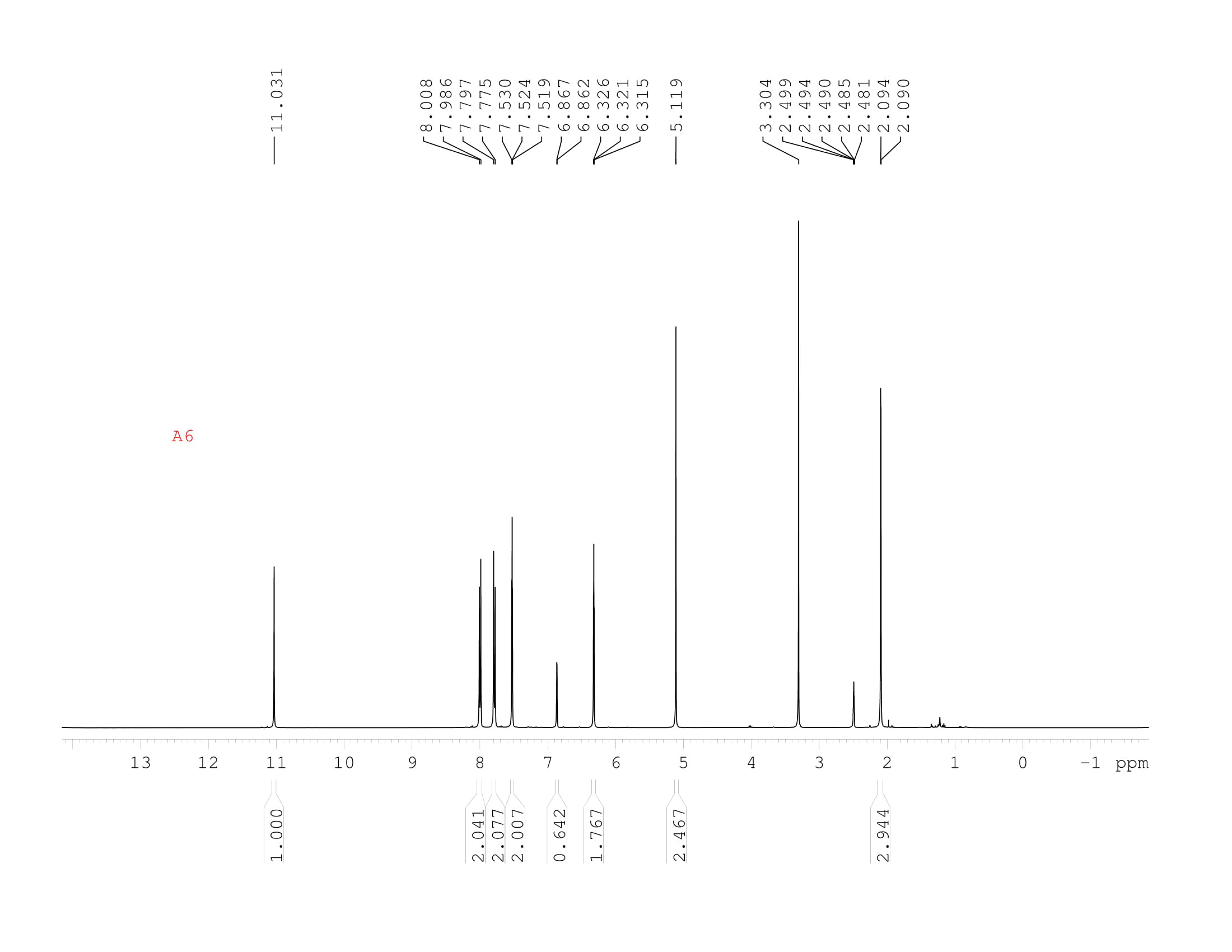

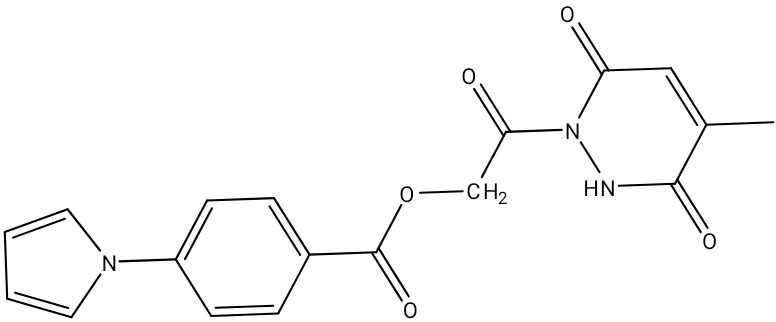


**Spectrum 29: ^13^C NMR Spectrum of compound 4f**


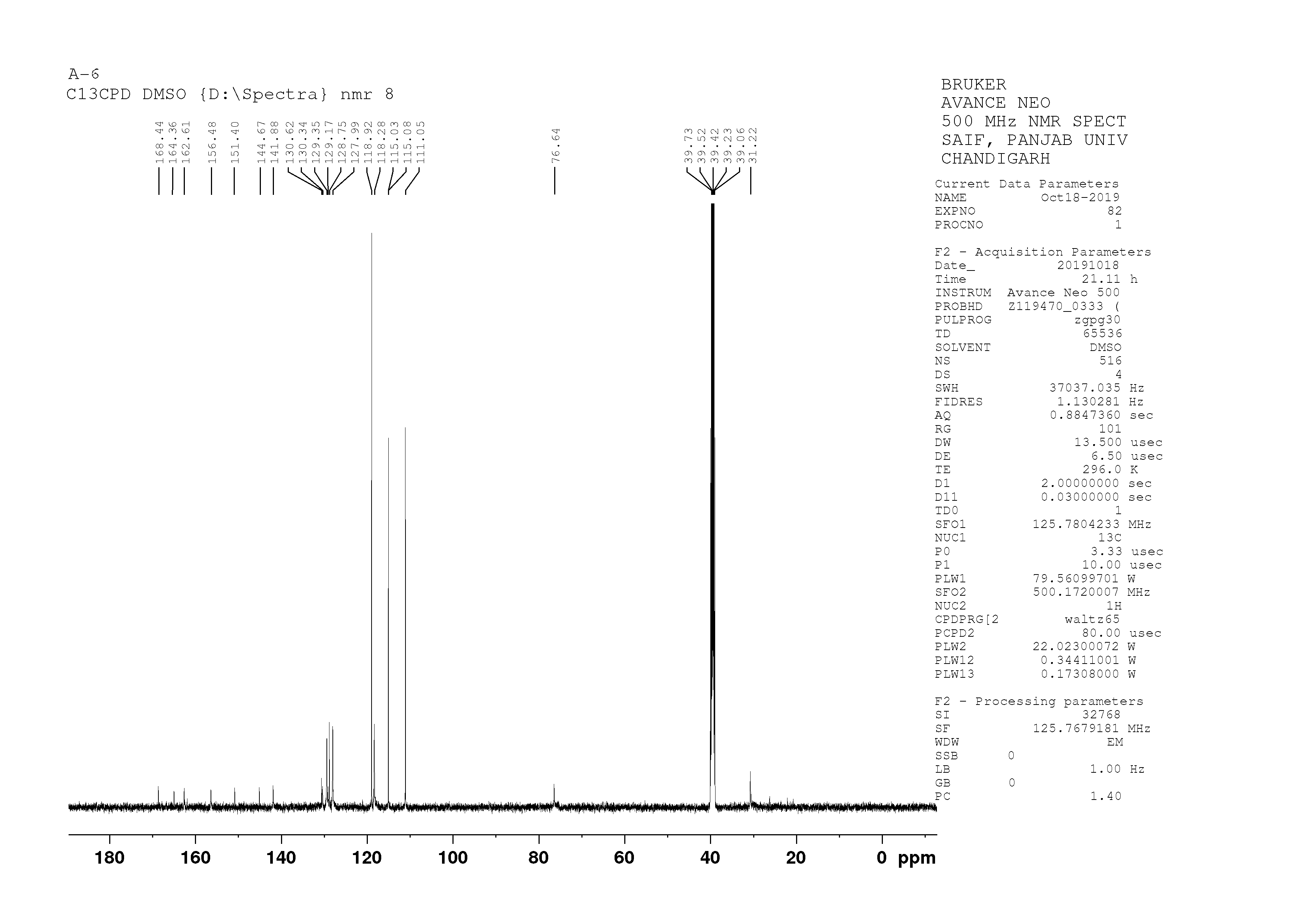

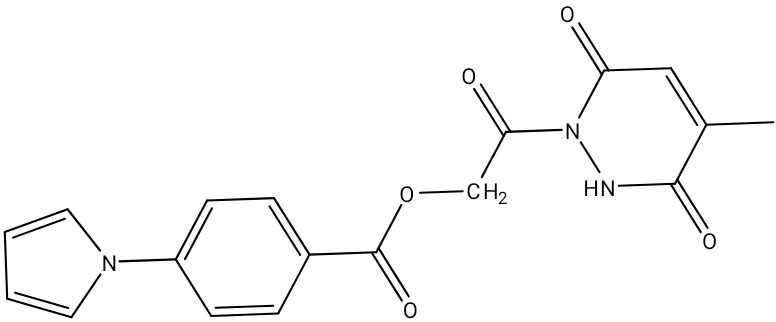


**Spectrum 30: Mass Spectrum of compound 4f**

**
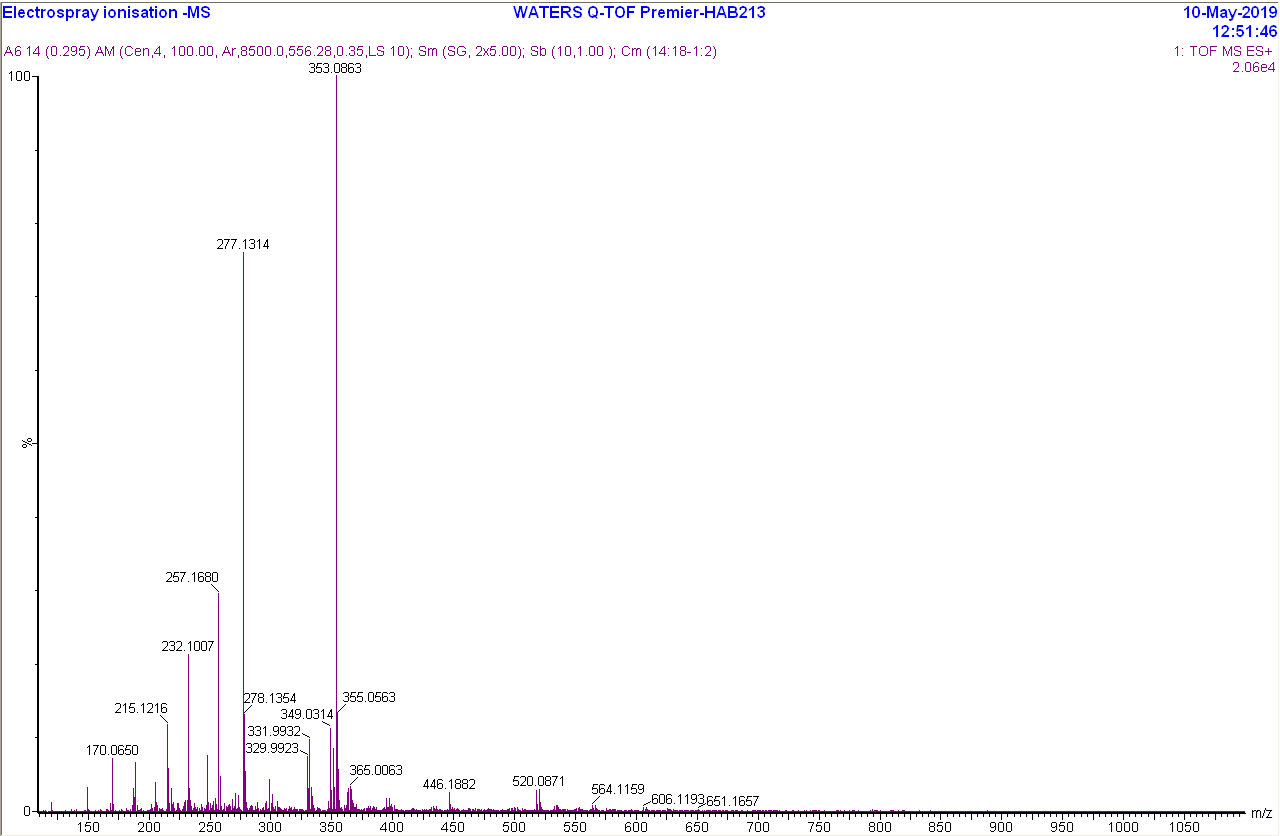
**
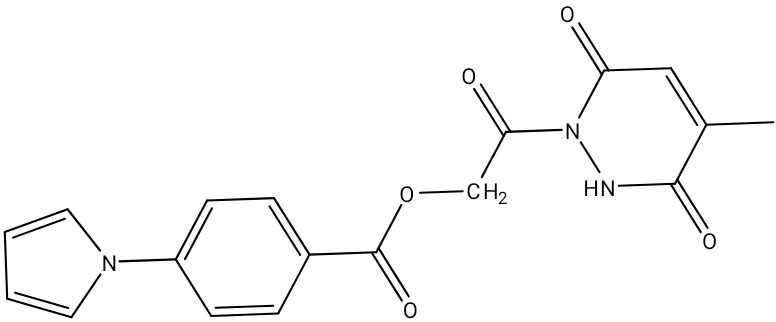


**Spectrum 31: IR Spectrum of compound 4g**


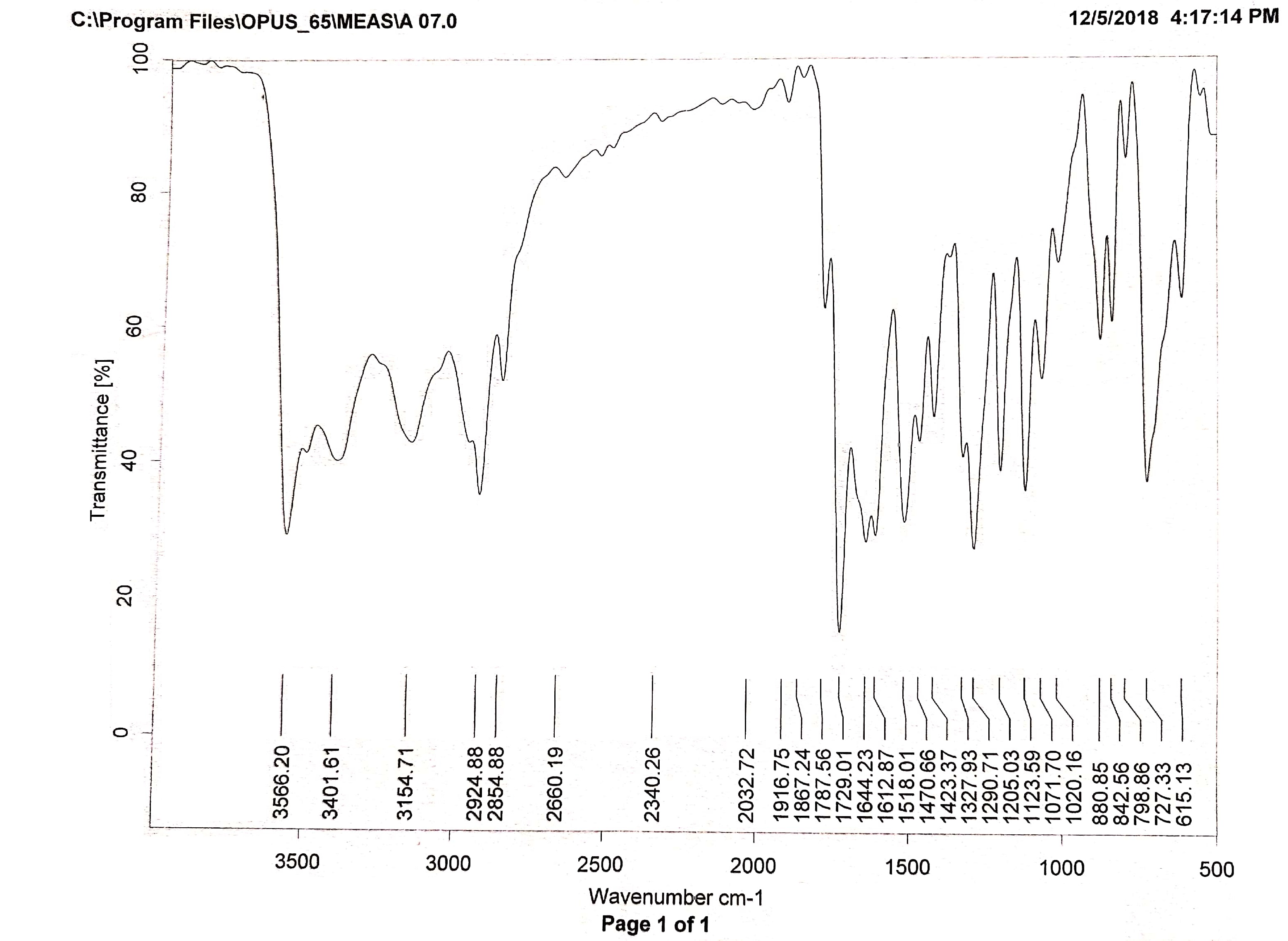

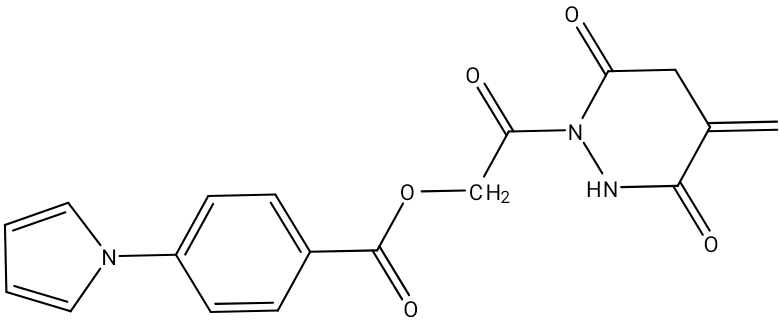


**Spectrum 32: ^1^H NMR Spectrum of compound 4g**


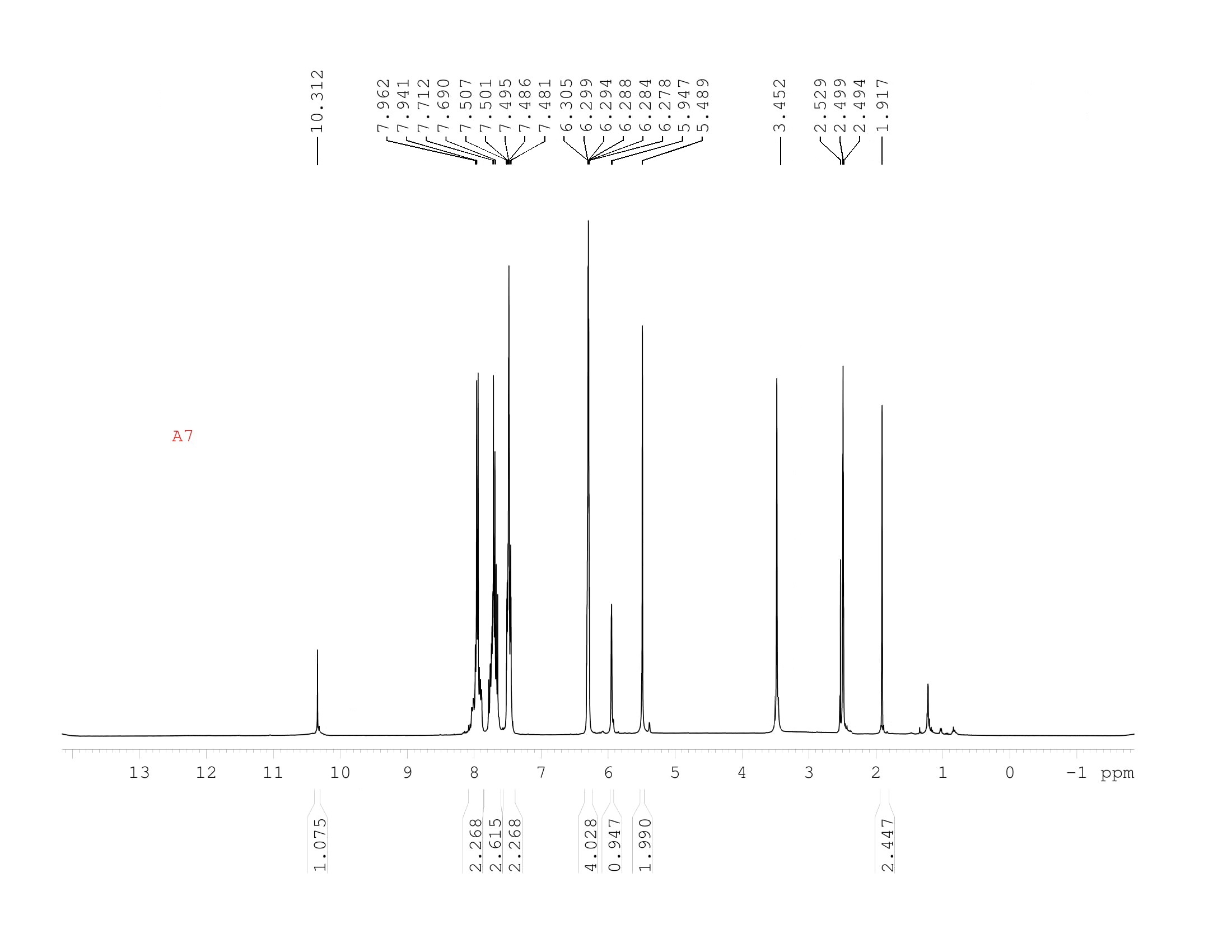

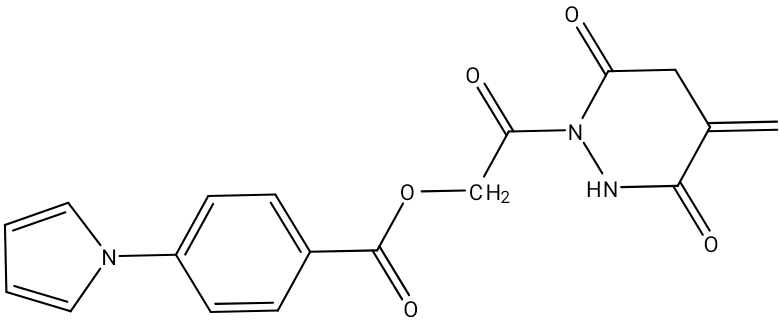


**Spectrum 33: ^13^C NMR Spectrum of compound 4g**


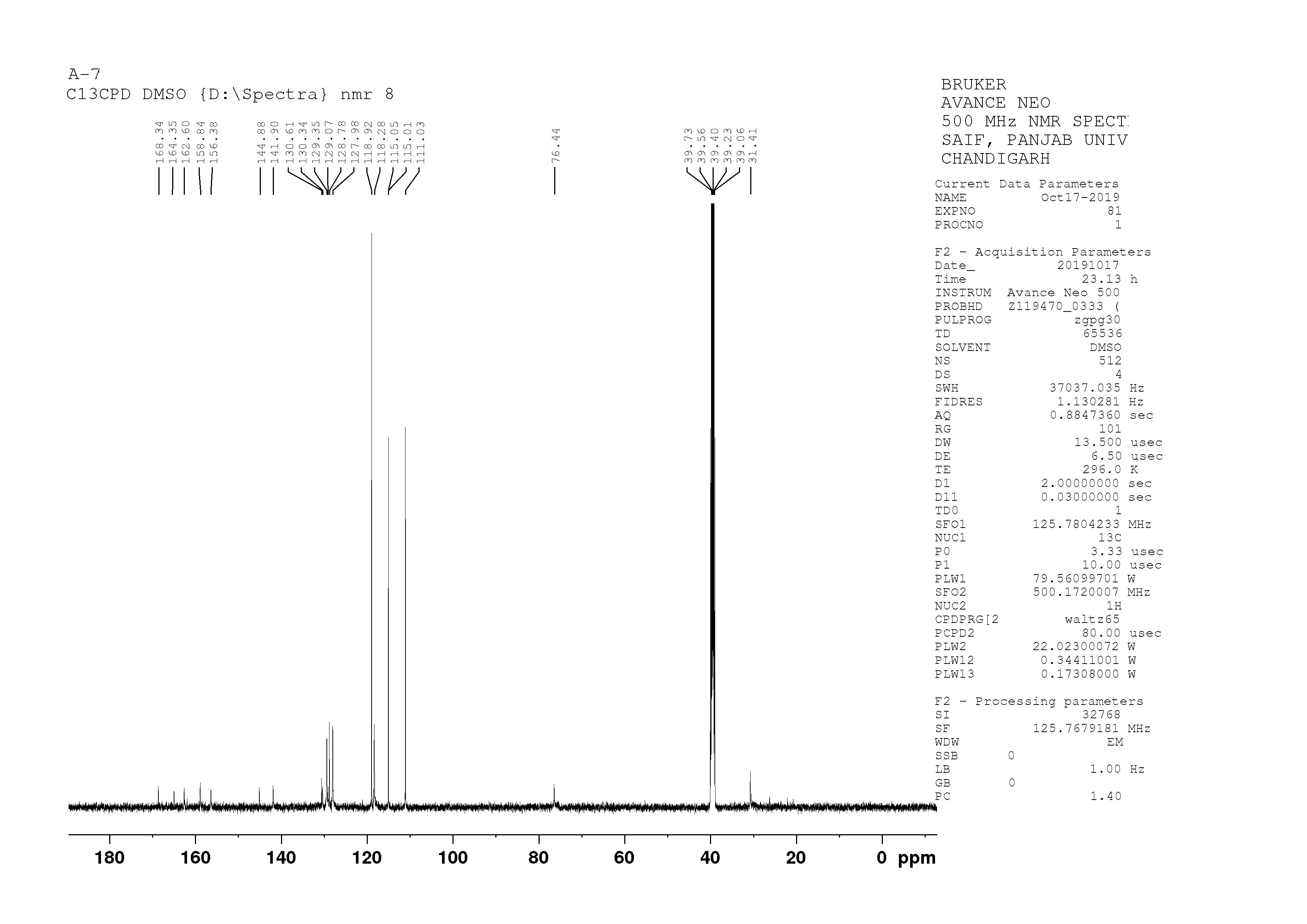

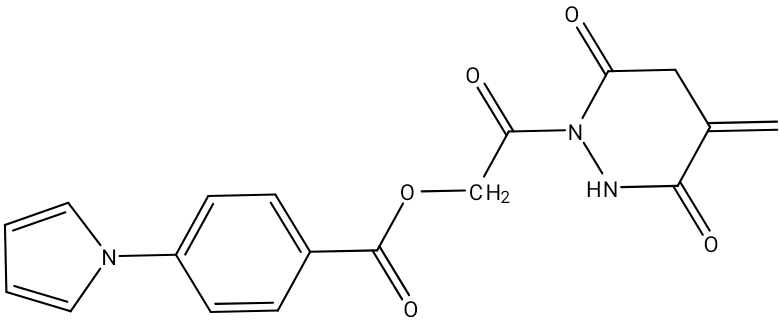


**Spectrum 34: Mass Spectrum of compound 4g**

**
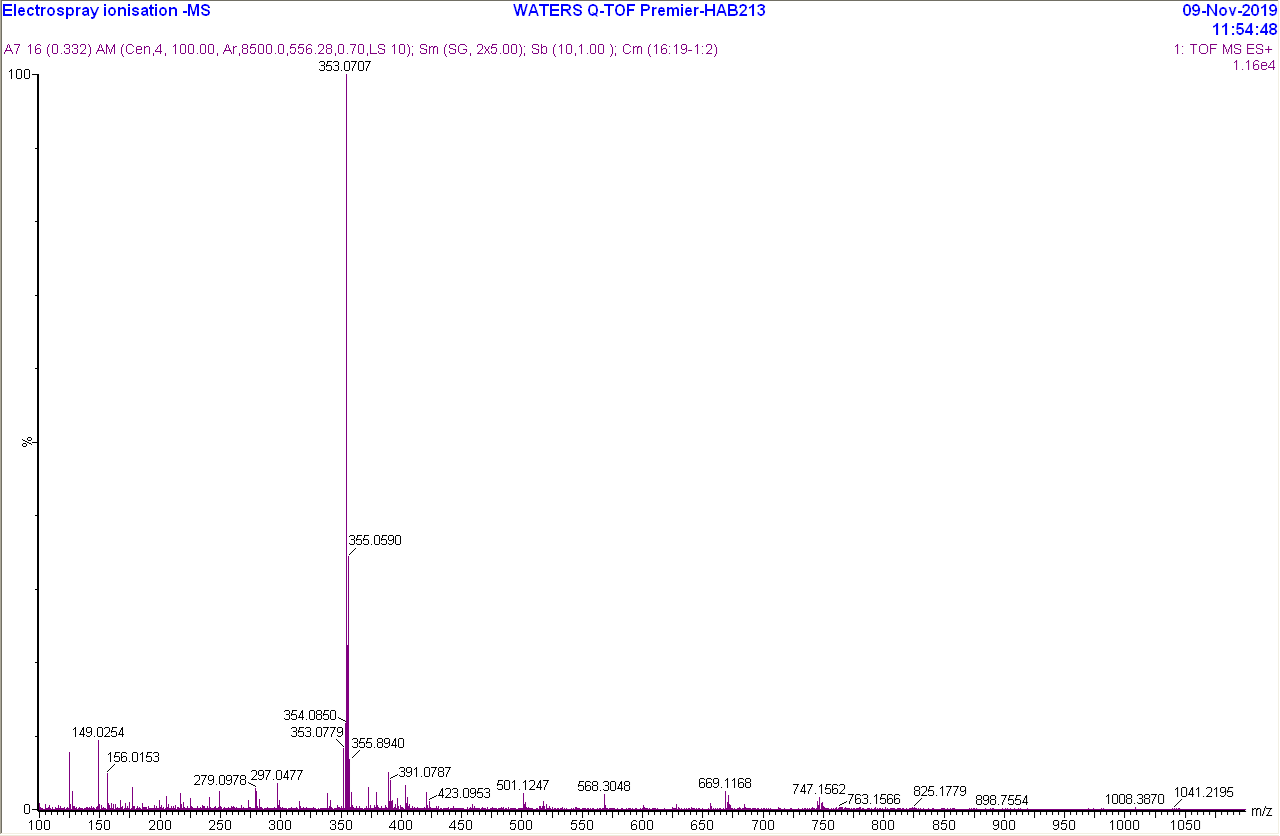
**
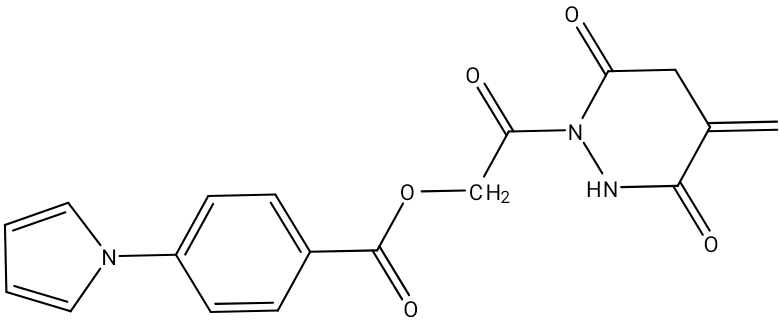


**Spectrum 35: IR Spectrum of compound 5a**


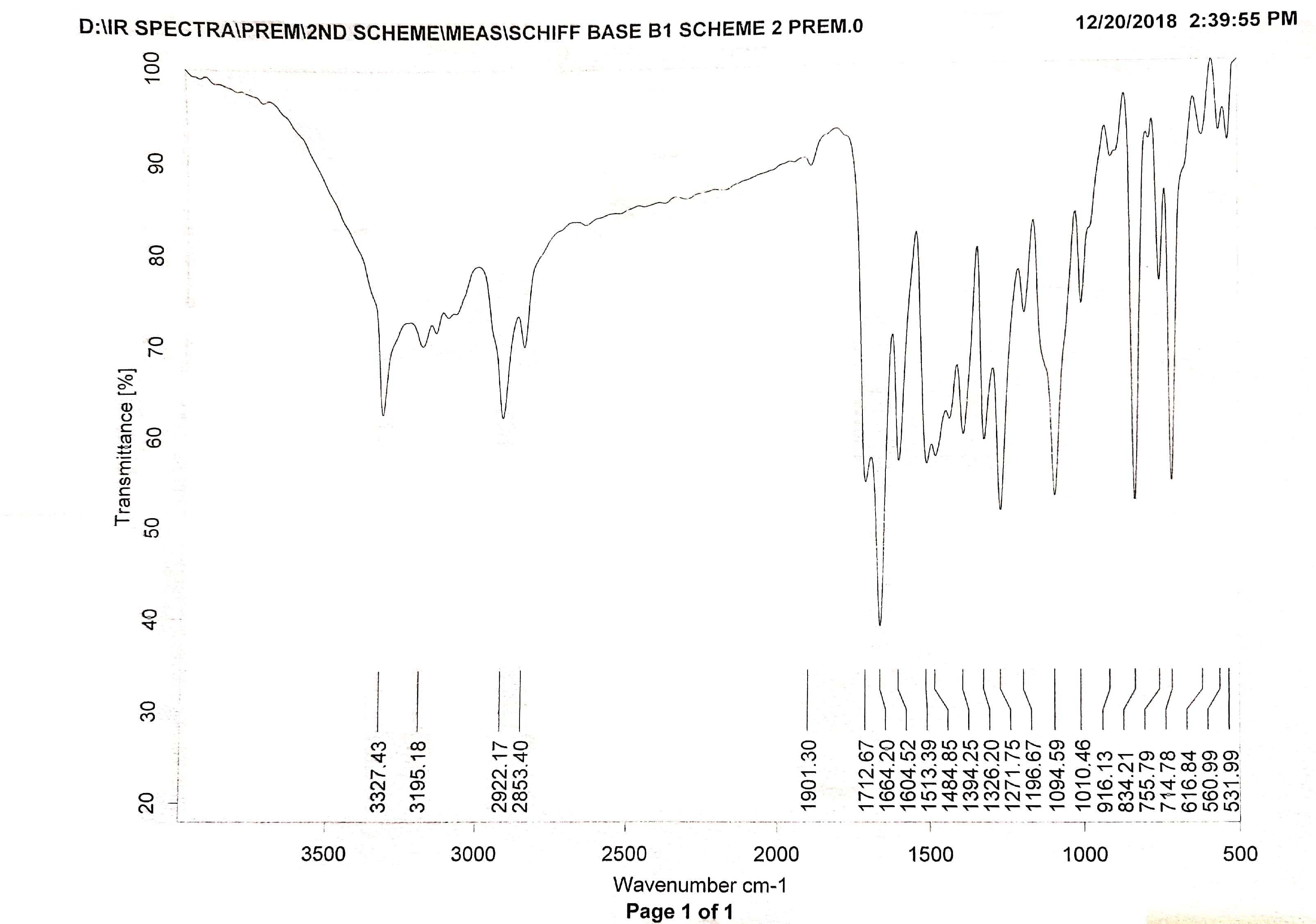

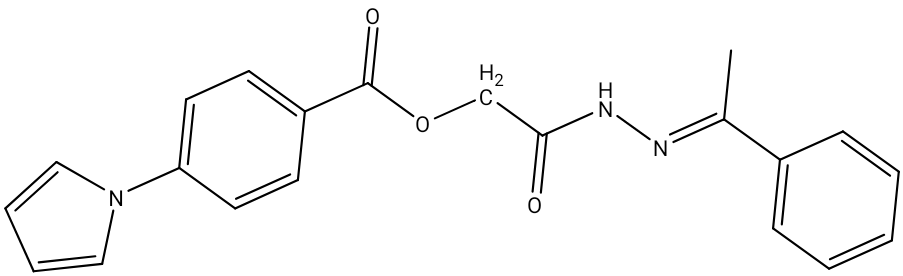


**Spectrum 36: ^1^H NMR Spectrum of compound 5a**


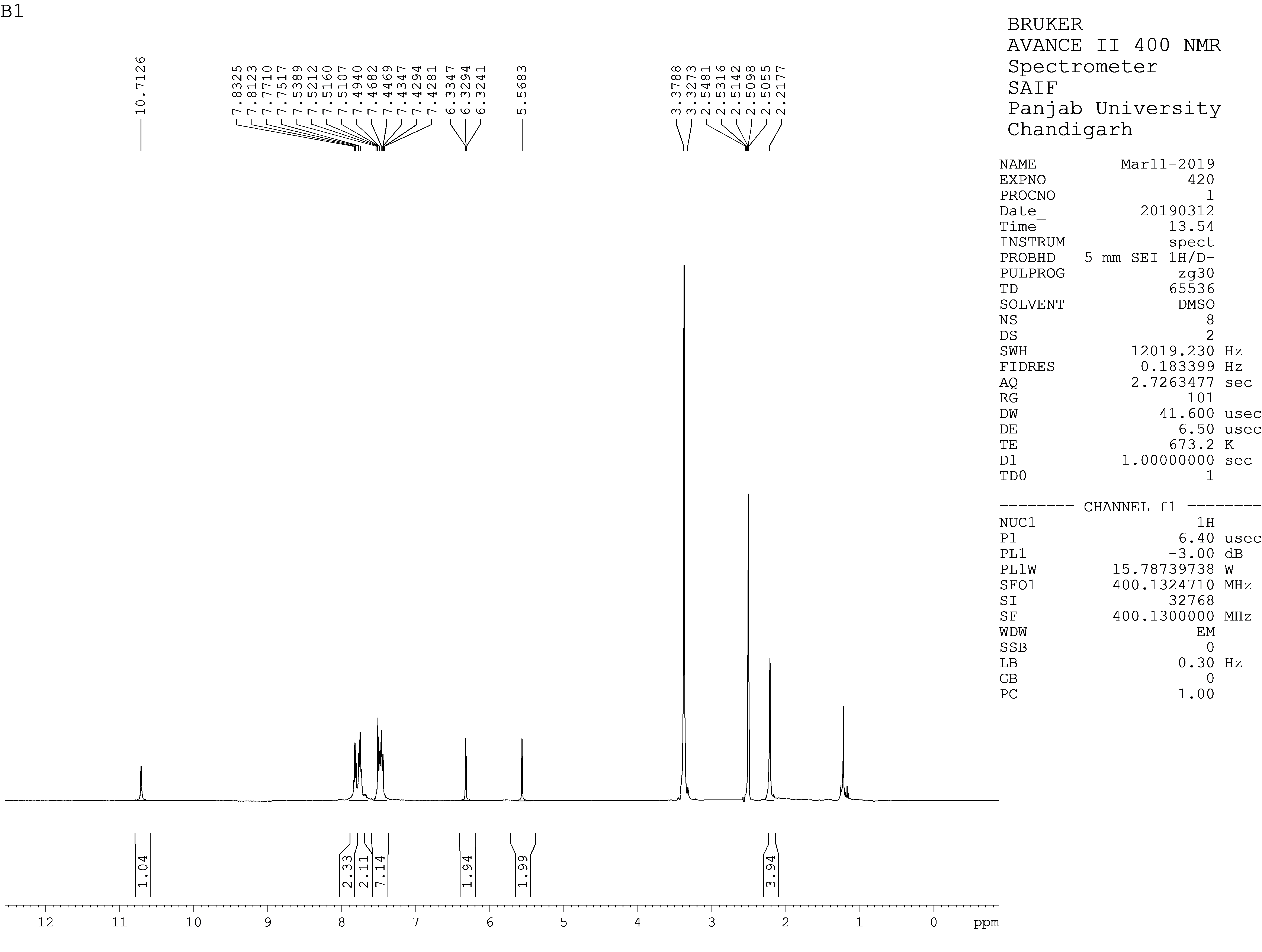

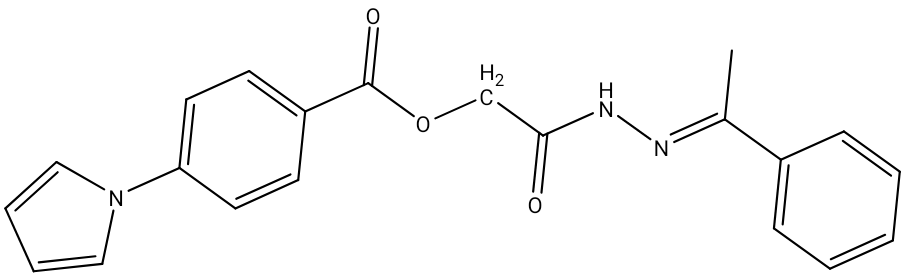


**Spectrum 37: ^13^C NMR Spectrum of compound 5a**
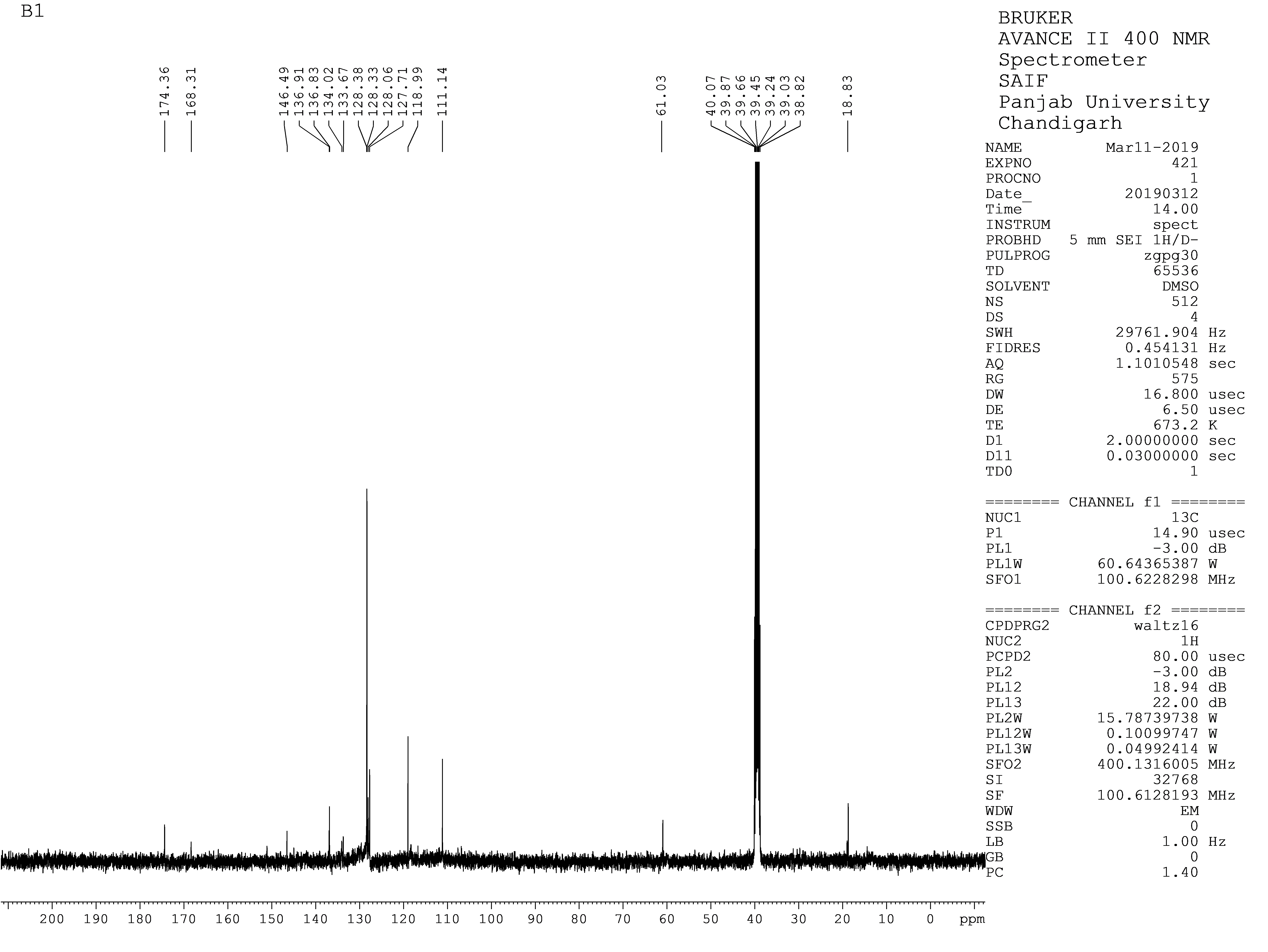

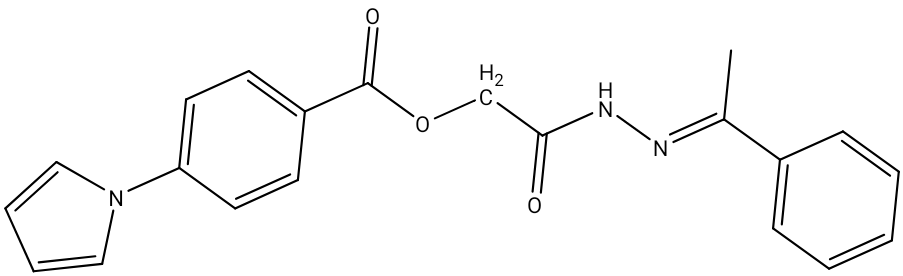


**Spectrum 37: Mass Spectrum of compound 5a**

**
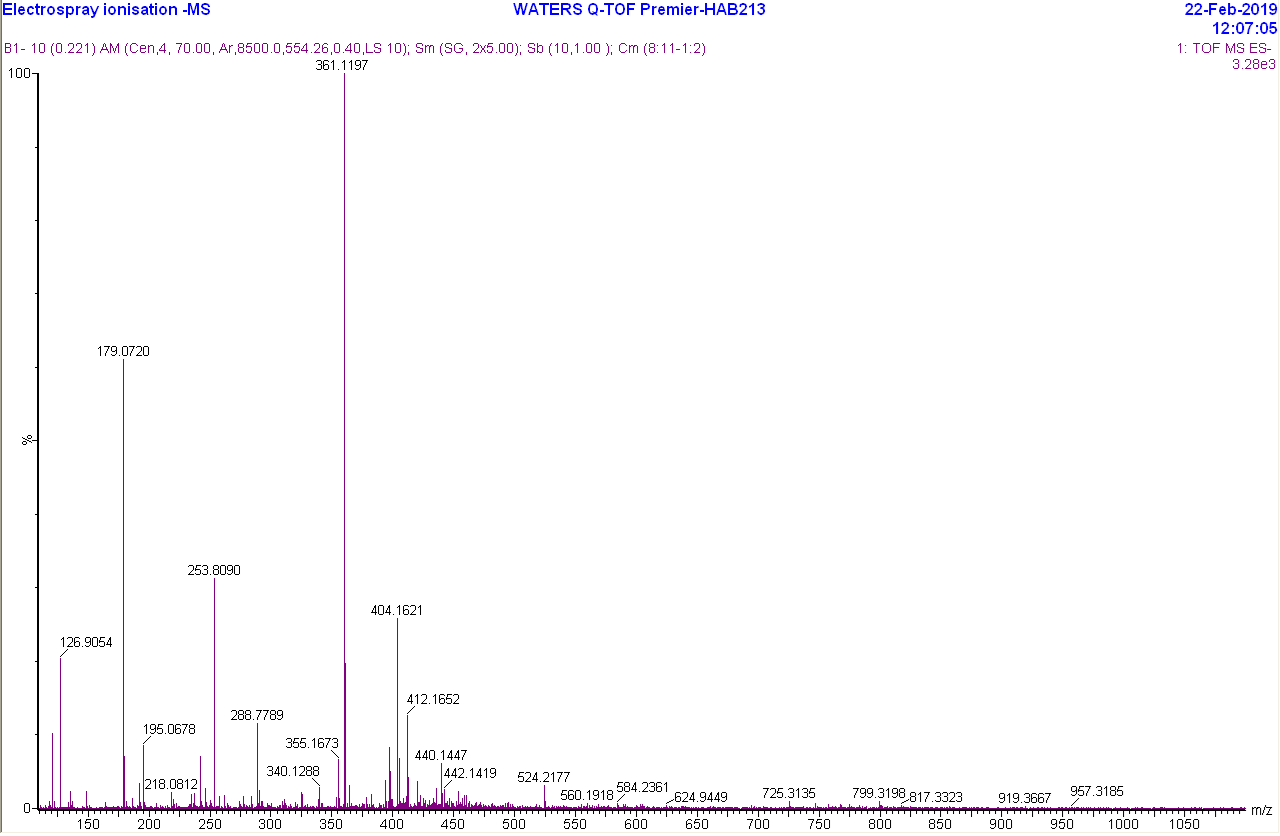
**
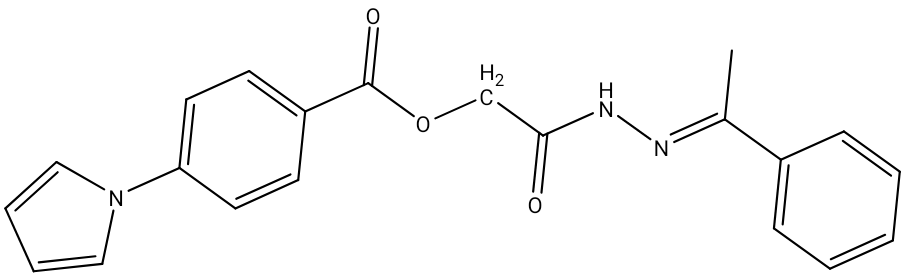


**Spectrum 38: IR Spectrum of compound 5b**


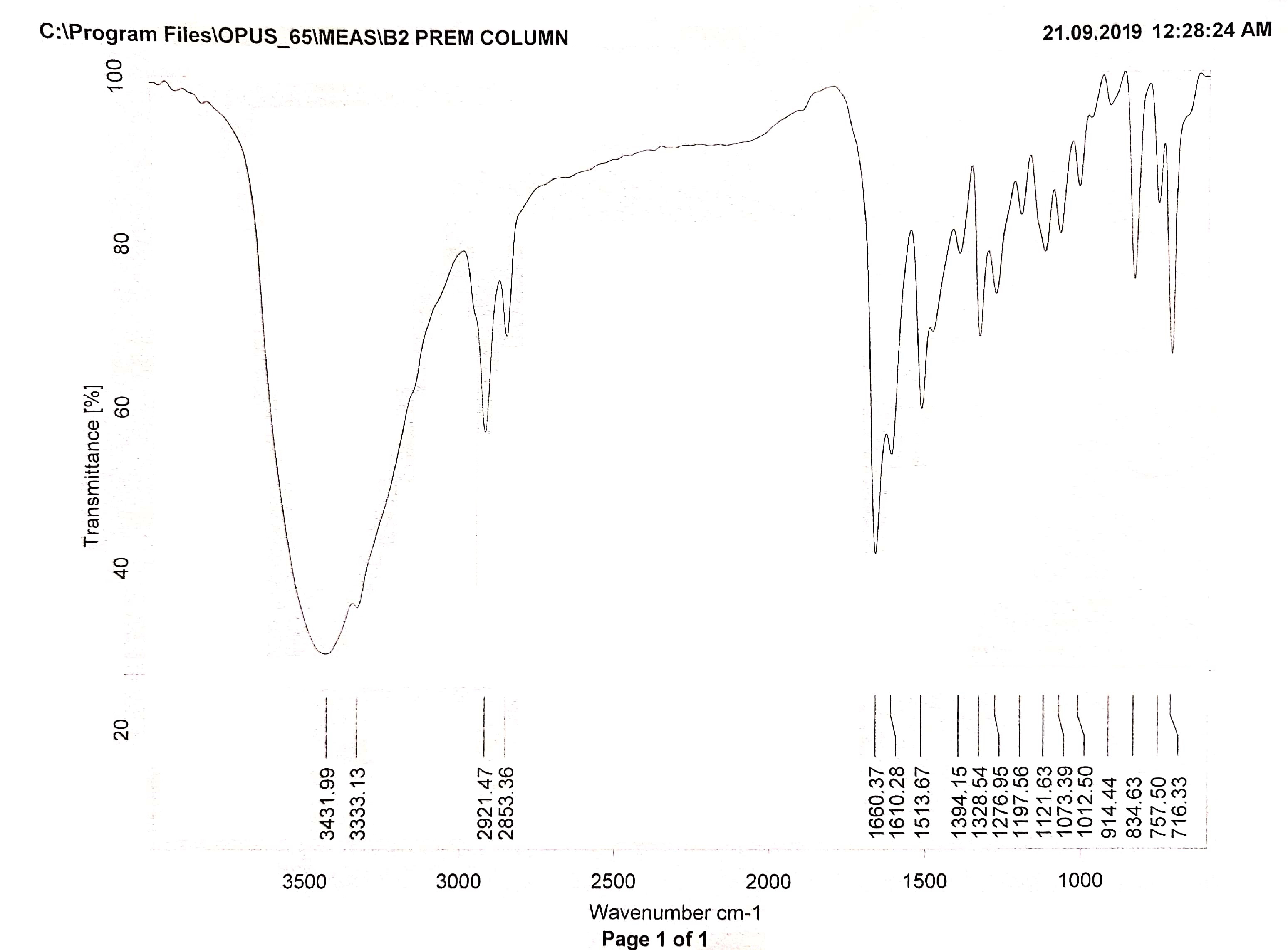

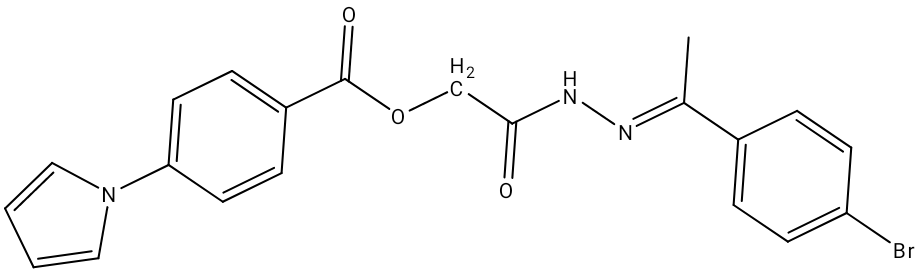


**Spectrum 39: ^1^H NMR Spectrum of compound 5b**


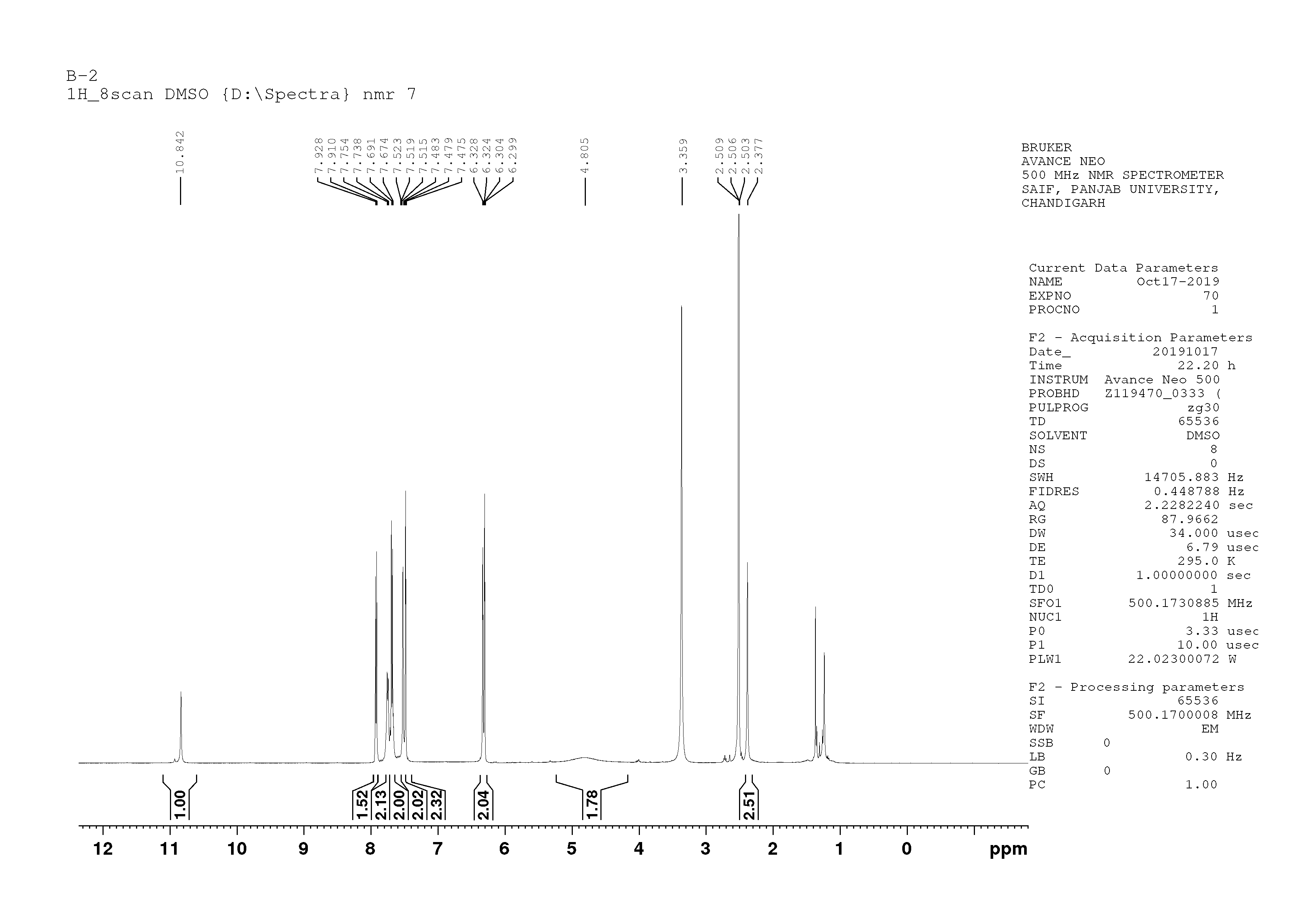

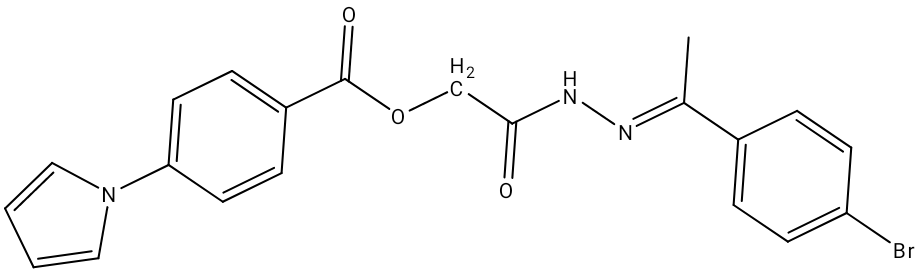


**Spectrum 40: ^13^C NMR Spectrum of compound 5b**


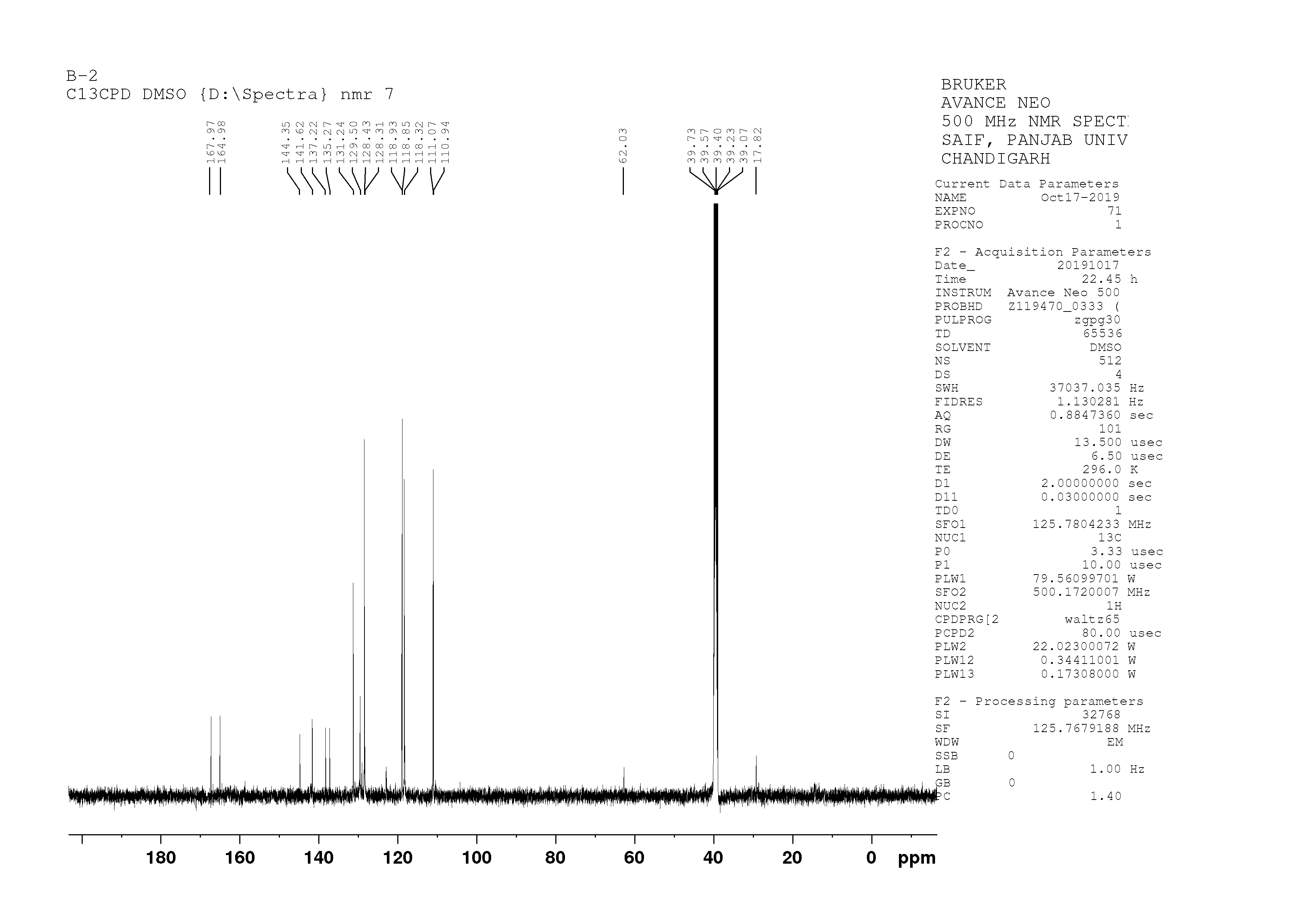

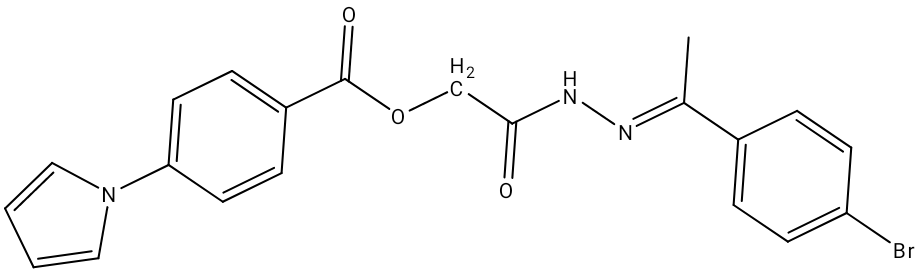


**Spectrum 41: Mass Spectrum of compound 5b**

**
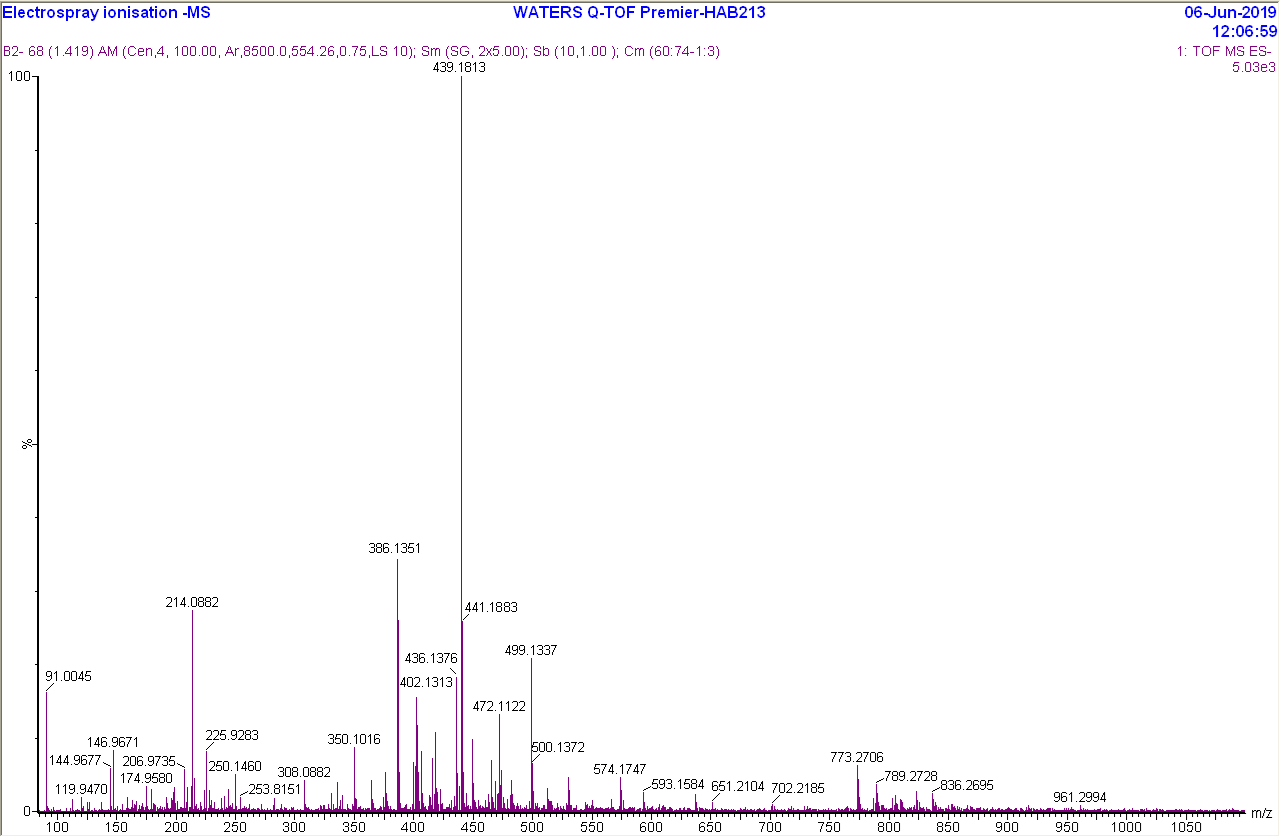
**
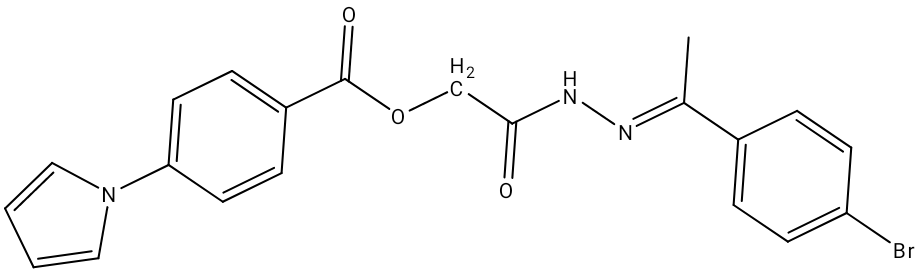


**Spectrum 42: IR Spectrum of compound 5c**


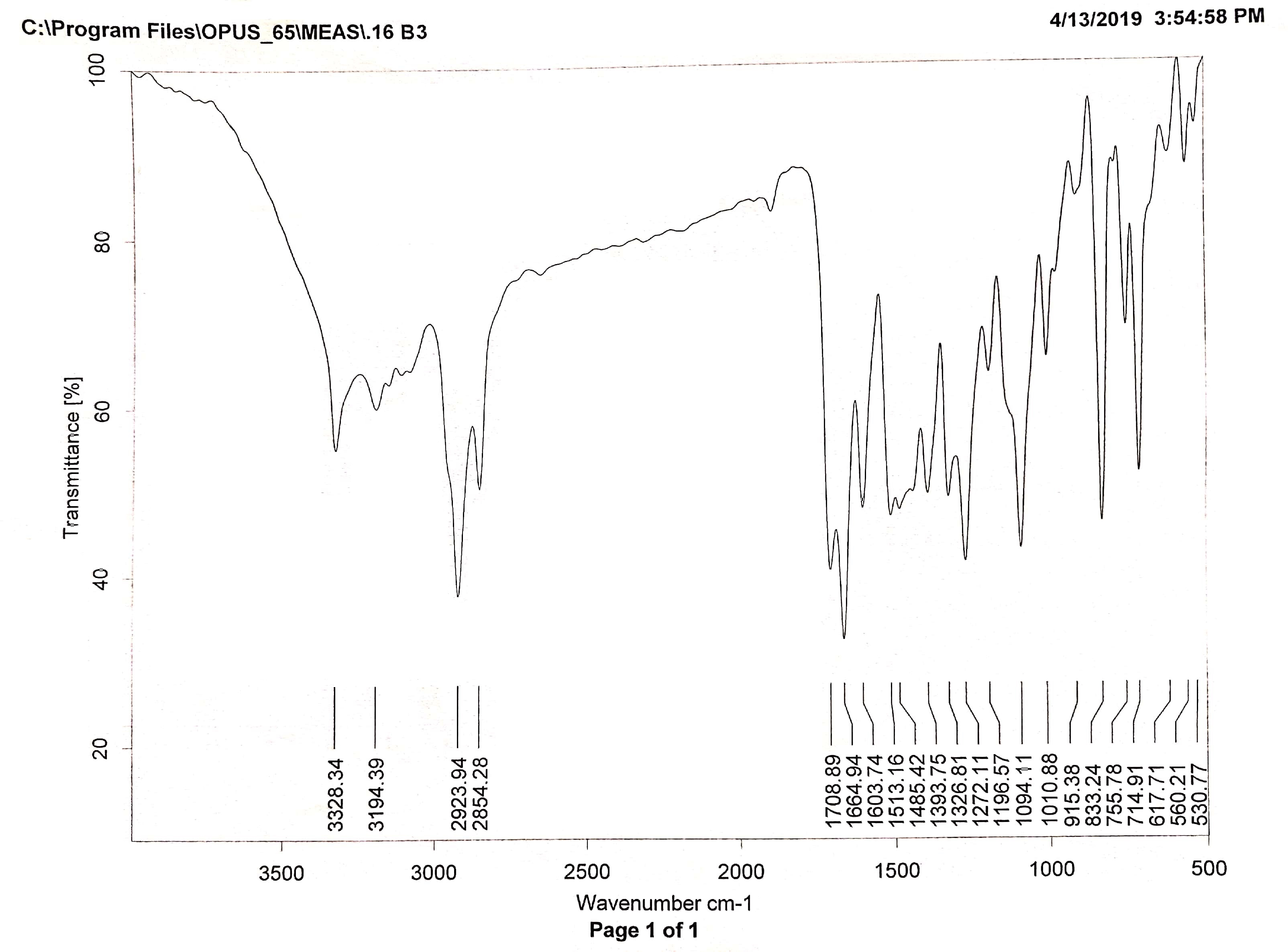

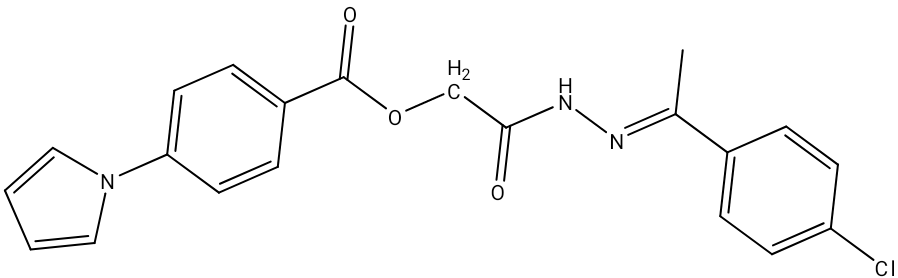


**Spectrum 43: ^1^H NMR Spectrum of compound 5c**


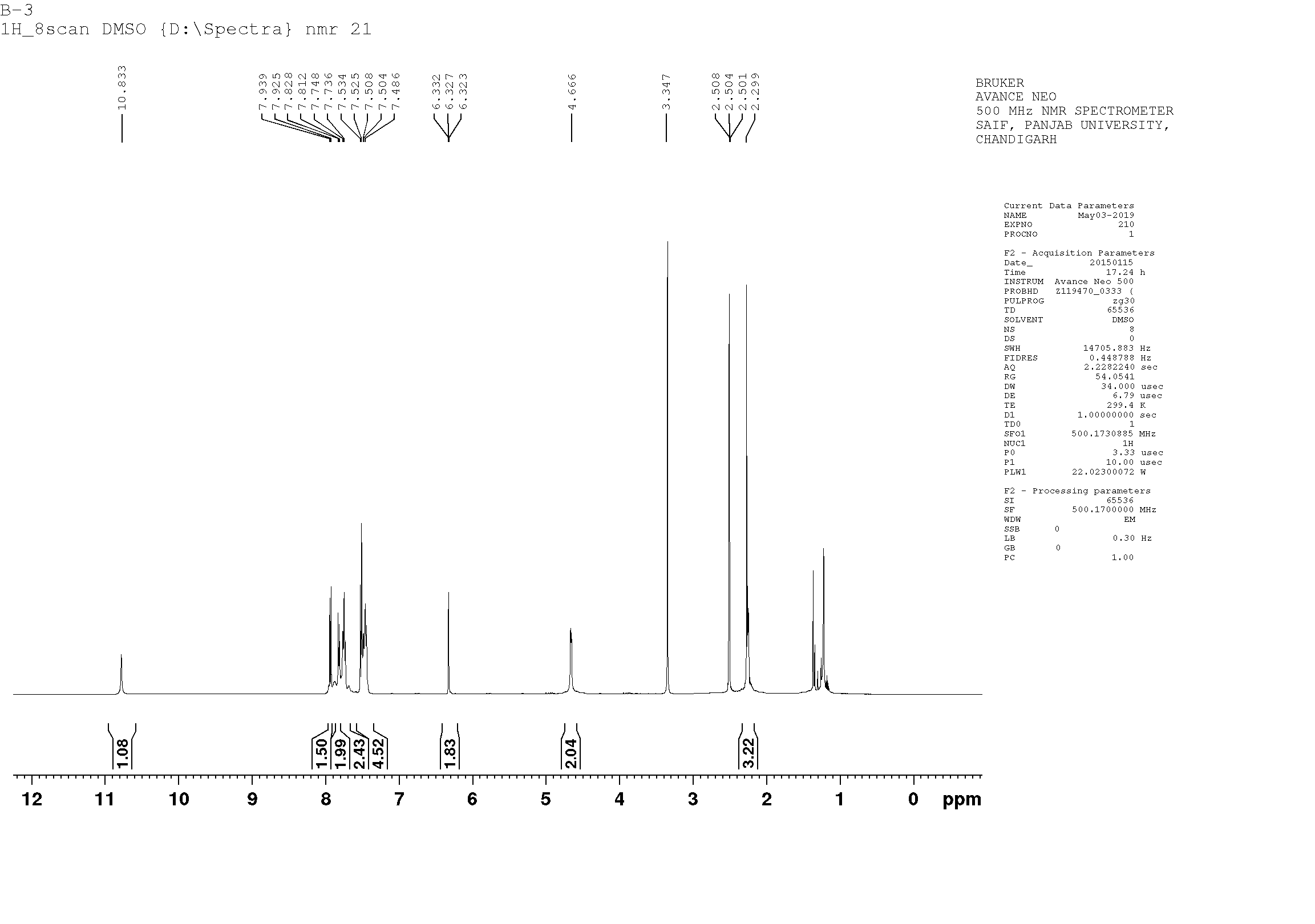

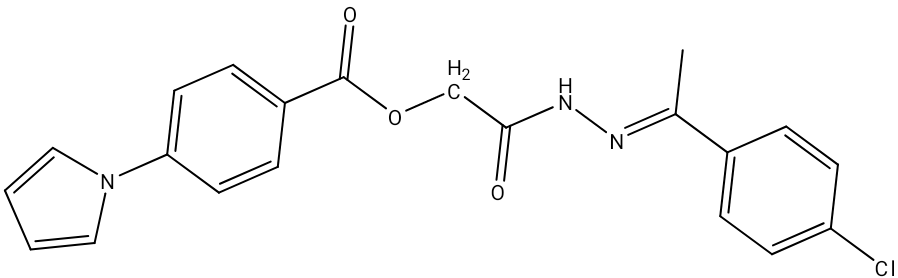


**Spectrum 44: ^13^C NMR Spectrum of compound 5c**


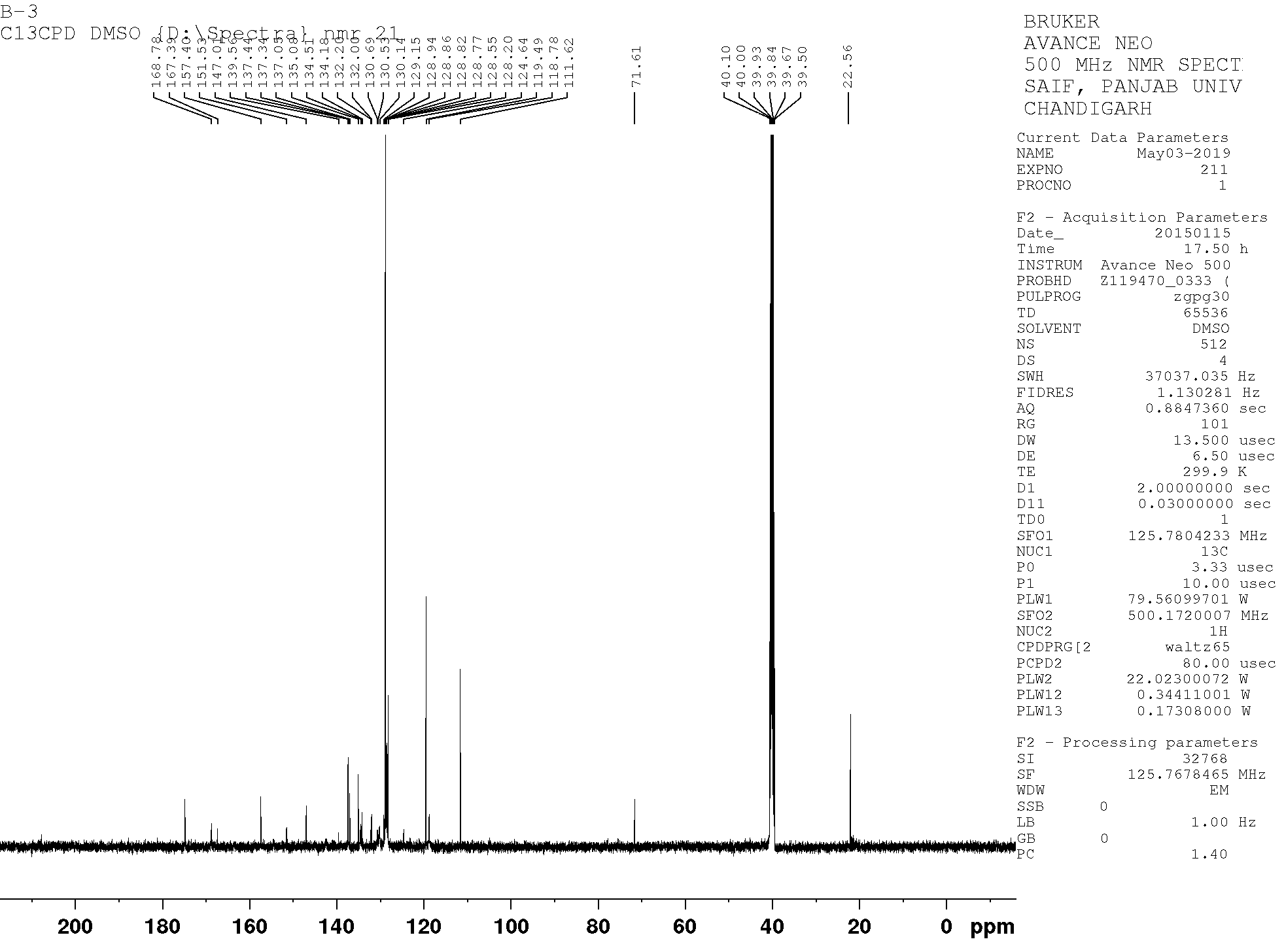

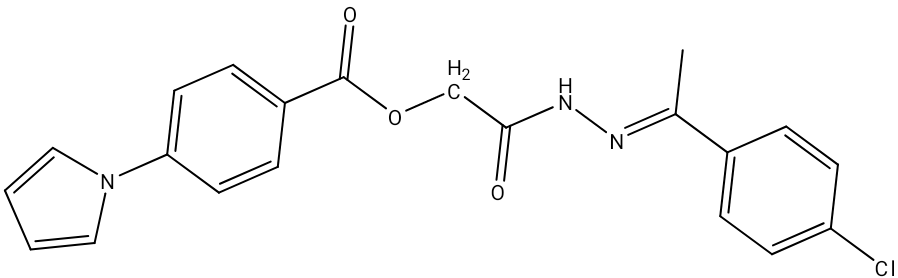


**Spectrum 45: Mass Spectrum of compound 5c**


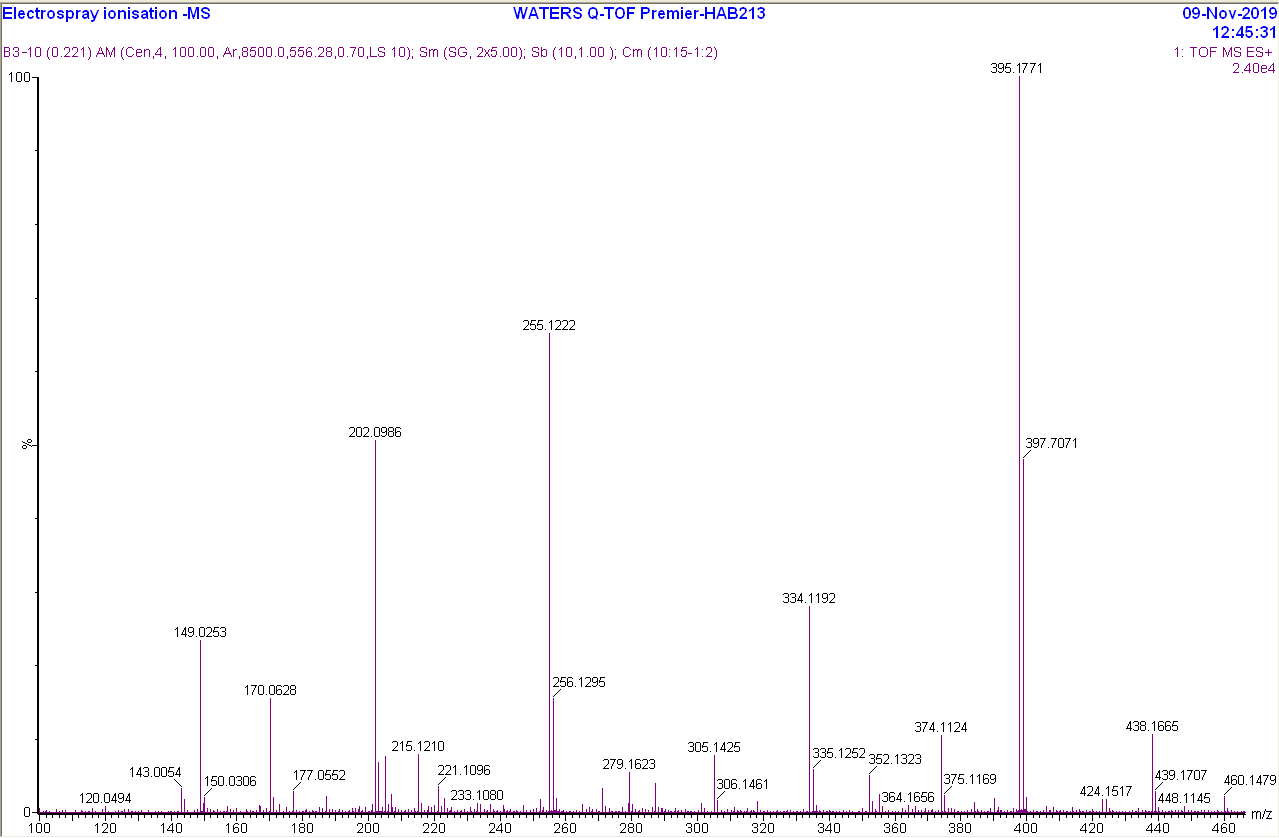

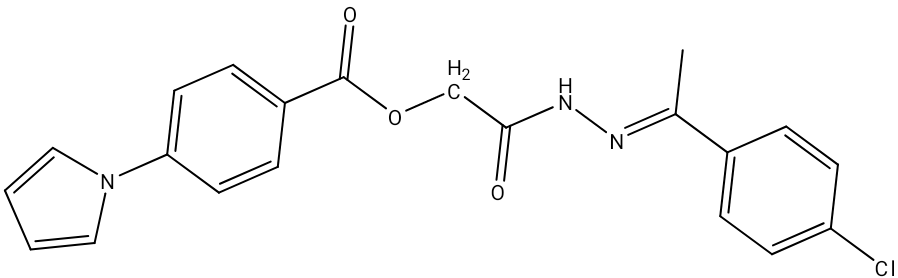


**Spectrum 46: IR Spectrum of compound 5d**


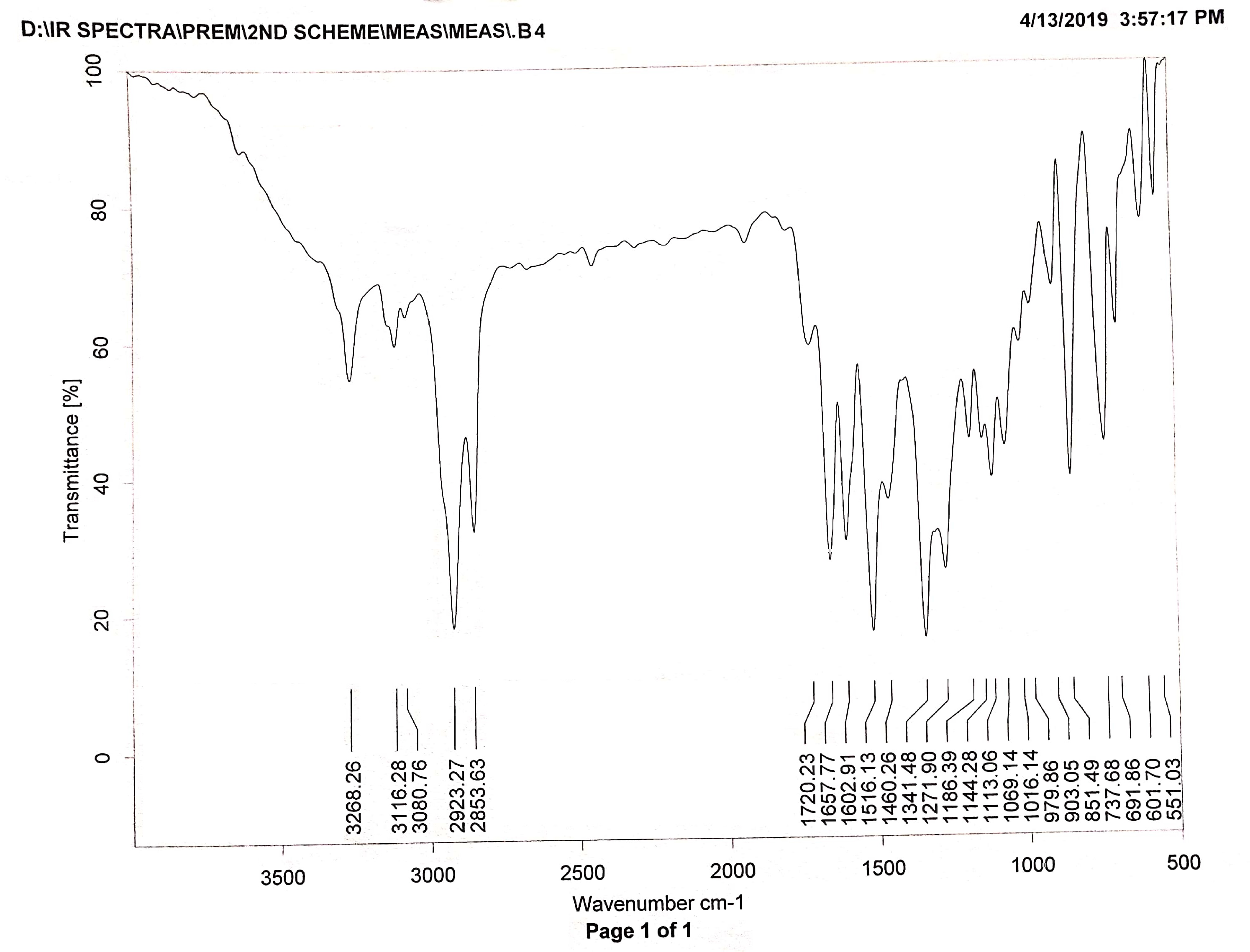

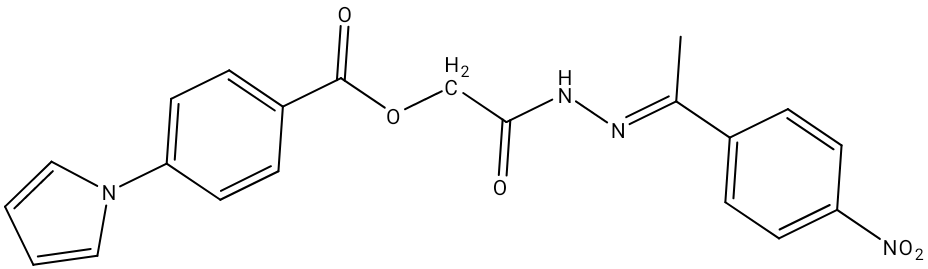


**Spectrum 47: ^1^H NMR Spectrum of compound 5d**


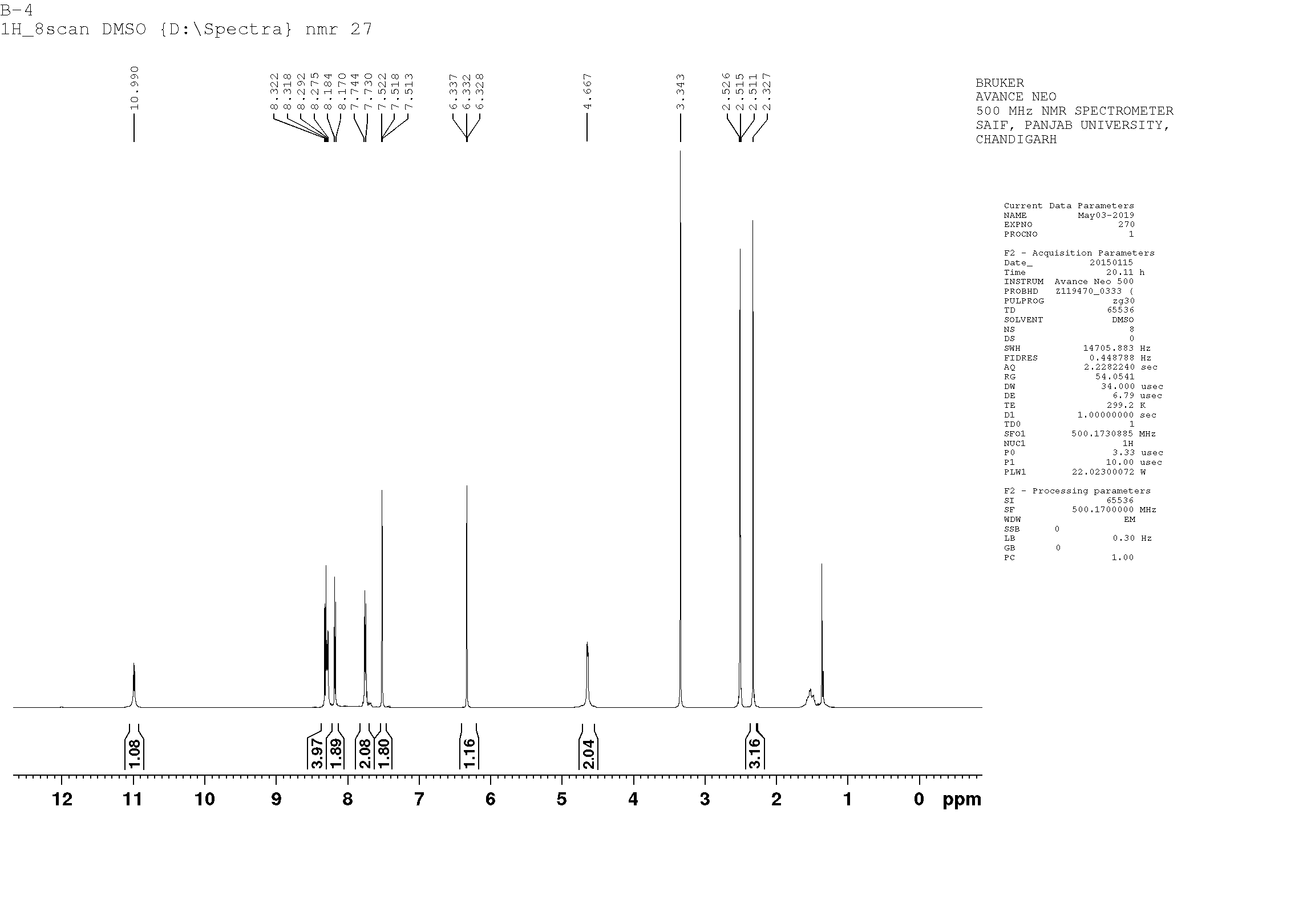

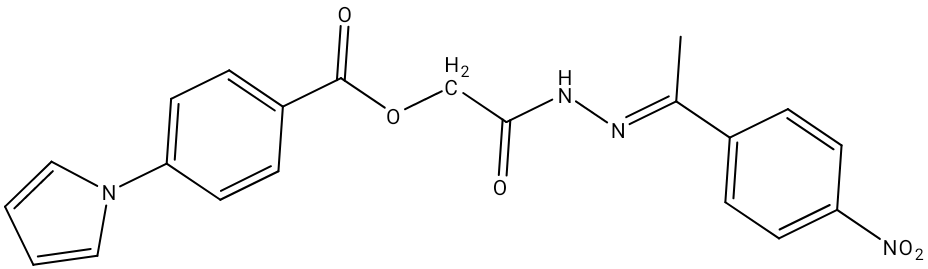


**Spectrum 48: ^13^C NMR Spectrum of compound 5d**


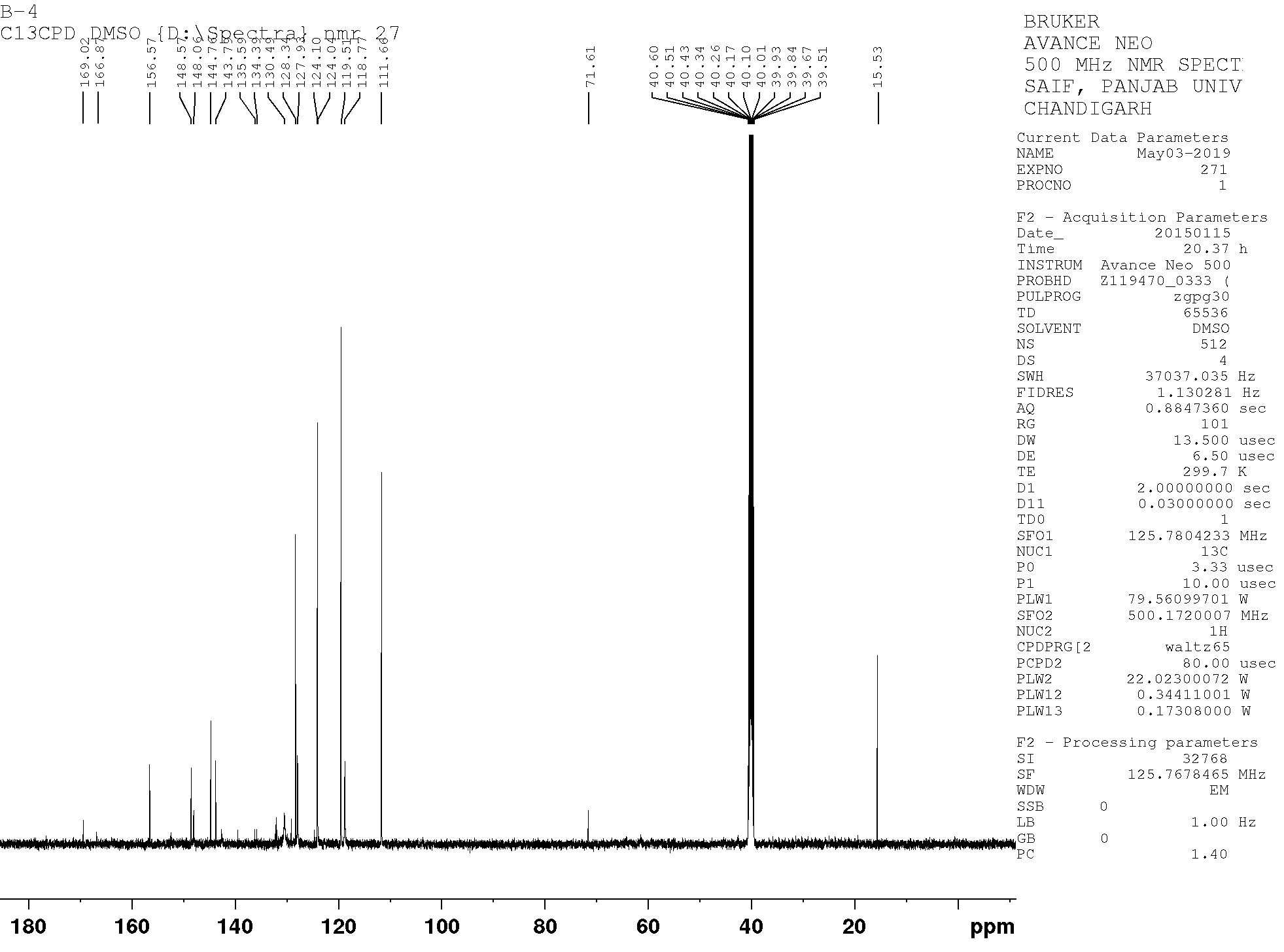

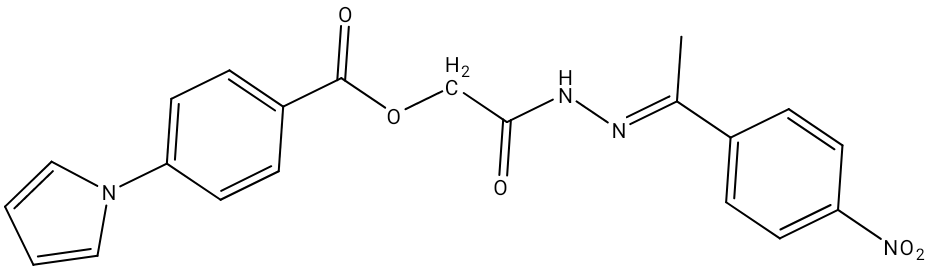


**Spectrum 49: Mass Spectrum of compound 5d**

**
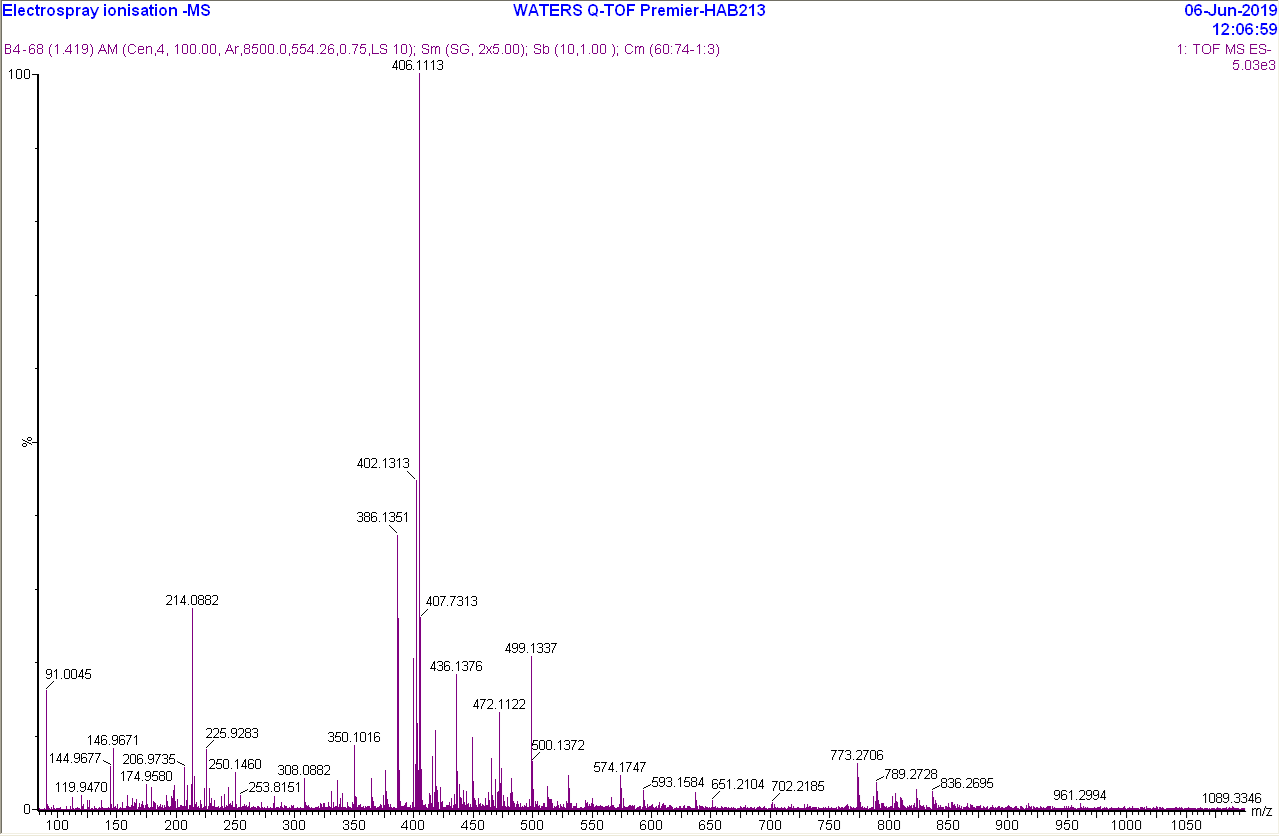
**
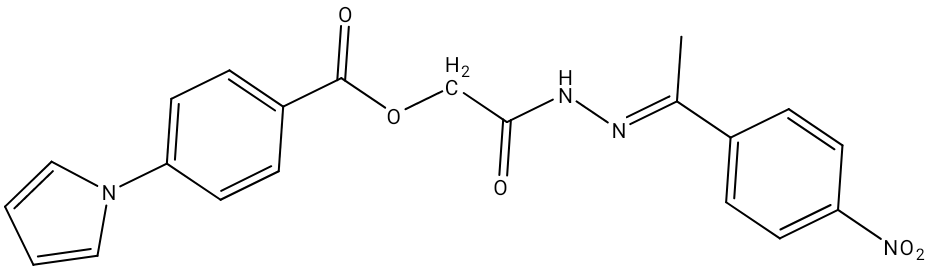


**Spectrum 50: IR Spectrum of compound 5e**

**Spectrum 51: ^1^H NMR Spectrum of compound 5e**

**Spectrum 52: ^13^C NMR Spectrum of compound 5e**

**Spectrum 53: Mass Spectrum of compound 5e**

**Spectrum 54: IR Spectrum of compound 5f**

**Spectrum 55: ^1^H NMR Spectrum of compound 5f**

**Spectrum 56: ^13^C NMR Spectrum of compound 5f**

**Spectrum 57: Mass Spectrum of compound 5f**

**Spectrum 58: IR Spectrum of compound 5g**

**Spectrum 59: ^1^H NMR Spectrum of compound 5g**

**Spectrum 60: ^13^C NMR Spectrum of compound 5g**

**Spectrum 61: Mass Spectrum of compound 5g**

**Spectrum 62: IR Spectrum of compound 5h**

**Spectrum 63: ^1^H NMR Spectrum of compound 5h**

**Spectrum 64: ^13^C NMR Spectrum of compound 5h**

**Spectrum 65: Mass Spectrum of compound 5h**

**Spectrum 66: IR Spectrum of compound 5i**

**Spectrum 67: ^1^H NMR Spectrum of compound 5i**

**Spectrum 68: ^13^C NMR Spectrum of compound 5i**

**Spectrum 69: Mass Spectrum of compound 5i**

**Spectrum 70: IR Spectrum of compound 5j**

**Spectrum 71: ^1^H NMR Spectrum of compound 5j**

**Spectrum 72: ^13^C NMR Spectrum of compound 5j**

**Spectrum 73: Mass Spectrum of compound 5j**

**Spectrum 74: IR Spectrum of compound 5k**

**Spectrum 75: ^1^H NMR Spectrum of compound 5k**

**Spectrum 76: ^13^C NMR Spectrum of compound 5k**

**Spectrum 77: Mass Spectrum of compound 5k**

**Spectrum 78: IR Spectrum of compound 5l**

**Spectrum 79: ^1^H NMR Spectrum of compound 5l**

**Spectrum 80: ^13^C NMR Spectrum of compound 5l**

**Spectrum 81: Mass Spectrum of compound 5l**

**Spectrum 82: IR Spectrum of compound 5m**

**Spectrum 83: ^1^H NMR Spectrum of compound 5m**

**Spectrum 84: ^13^C NMR Spectrum of compound 5m**

**Spectrum 85: Mass Spectrum of compound 5m**

**Spectrum 86: IR Spectrum of compound 5n**

**Spectrum 87: ^1^H NMR Spectrum of compound 5n**

**Spectrum 88: ^13^C NMR Spectrum of compound 5n**

**Spectrum 88: Mass Spectrum of compound 5n**

**Spectrum 89: IR Spectrum of compound 5o**

**Spectrum 90: ^1^H NMR Spectrum of compound 5o**

**Spectrum 91: ^13^C NMR Spectrum of compound 5o**

**Spectrum 92: Mass Spectrum of compound 5o**

**Spectrum 93: IR Spectrum of compound 5p**

**Spectrum 94: ^1^H NMR Spectrum of compound 5p**

**Spectrum 95: ^13^C NMR Spectrum of compound 5p**

**Spectrum 96: Mass Spectrum of compound 5p**

**Spectrum 97: IR Spectrum of compound 6a**

**Spectrum 98: ^1^H NMR Spectrum of compound 6a**

**Spectrum 99: ^13^C NMR Spectrum of compound 6a**

**Spectrum 100: Mass Spectrum of compound 6a**

**Spectrum 101: IR Spectrum of compound 6b**

**Spectrum 102: ^1^H NMR Spectrum of compound 6b**

**Spectrum 103: ^13^C NMR Spectrum of compound 6b**

**Spectrum 104: Mass Spectrum of compound 6b**

**Spectrum 105: IR Spectrum of compound 6c**

**Spectrum 106: ^1^H NMR Spectrum of compound 6c**

**Spectrum 107: ^13^C NMR Spectrum of compound 6c**

**Spectrum 108: Mass Spectrum of compound 6c**

**Spectrum 109: IR Spectrum of compound 6d**

**Spectrum 110: ^1^H NMR Spectrum of compound 6d**

**Spectrum 111: ^13^C NMR Spectrum of compound 6d**

**Spectrum 112: Mass Spectrum of compound 6d**

**Spectrum 113: IR Spectrum of compound 6e**

**Spectrum 114: ^1^H NMR Spectrum of compound 6e**

**Spectrum 115: ^13^C NMR Spectrum of compound 6e**

**Spectrum 116: Mass Spectrum of compound 6e**
