# Supplementary material for: Design, synthesis and in silico molecular modelling studies of 2-Hydrazineyl-2-oxoethyl-4-(1H-pyrrol-1-yb) benzoate derivatives: a potent dual DHFR and ENR-reductase inhibitors with antitubercular, antibacterial and cytotoxic potential
Source: PLoS One. 2025 May 19;20(5):e0323702. doi: 10.1371/journal.pone.0323702 (PMC12088000; doi:10.1371/journal.pone.0323702)
Supplement: S2 File — (DOC) [file pone.0323702.s002.doc]

**Design, Synthesisiandi*IniSilico*iMoleculariModelling Studiesiofi2-Hydrazineyl-2-oxoethyl-4-(1*H*-pyrrol-1-yb) Benzoate derivatives: A Potent Dual DHFR And ENR-Reductase Inhibitors with Antitubercular, Antibacterial and Cytotoxic Potential.**

Mater H. Mahnashi^1^, Prem Kumar S. R^2^, Ahmed Abdullah Al Awadh^3^, Ibrahim Abdullah Almazni^3^, Yahya I. Asiri^4^, Ibrahim Ahmed Shaikh^5^, Basheerahmed Abdulaziz Mannasaheb^6^, Sravanthi Avunoori^7^, Aejaz Abdullatif Khan^8^, Shrinivas D. Joshi^7^*

^1^Department of Pharmaceutical Chemistry, College of Pharmacy, King Khalid University, Abha, Saudi Arabia.

^2^Department of Pharmaceutical Quality Assurance, Sree Siddaganaga College of Pharmacy, Tumkur, Karnataka, India-572103 [prempharma77@gmail.com](mailto:prempharma77@gmail.com)

^3^Department of Clinical Laboratory Sciences, Faculty of Applied Medical Sciences, Najran University, 1988, Najran, 61441, Saudi Arabia. [aaalawadh@nu.edu.sa](mailto:aaalawadh@nu.edu.sa); [eaalmazni@nu.edu.sa](mailto:eaalmazni@nu.edu.sa)

^4^Department of Pharmacology, College of Pharmacy, King Khalid University, Abha, Saudi Arabia.

[yialmuawad@kku.edu.sa](mailto:yialmuawad@kku.edu.sa)

^5^Department of Pharmacology, College of Pharmacy, Najran University, Najran, 66462, Saudi Arabia. [iashikh@nu.edu.sa](mailto:iashikh@nu.edu.sa)

^6^Department of Pharmacy Practice, College of Pharmacy, AlMaarefa University, P.O. Box 71666, Riyadh 11597, Saudi Arabia. [bmannasaheb@um.edu.sa](mailto:bmannasaheb@um.edu.sa)

^7^Novel Drug Design and Discovery Laboratory, Department of Pharmaceutical Chemistry, SET’s College of Pharmacy, Sangolli Rayanna Nagar, Dharwad-580002, Karnataka, India. [sravanthi.avunoori@gmail.com](mailto:sravanthi.avunoori@gmail.com)

^8^Department of General Science, Ibn Sina National College for Medical Studies, Jeddah, Saudi Arabia. [aeju_kh@yahoo.com](mailto:aeju_kh@yahoo.com)

* Corresponding Author: **Shrinivas D. Joshi** e-mail: [shrinivasdj@rediffmail.com](mailto:shrinivasdj@rediffmail.com)

**Figure S1 (A-B):** (A) Docked mode of **4TZK ligand**; (B) 3D docked view of the **4TZK ligand**. Binding site residues; orange colored TYR158 amino acid orange-colored, red-orange colored co-factor NAD+ and the molecule is colored according to atom type.


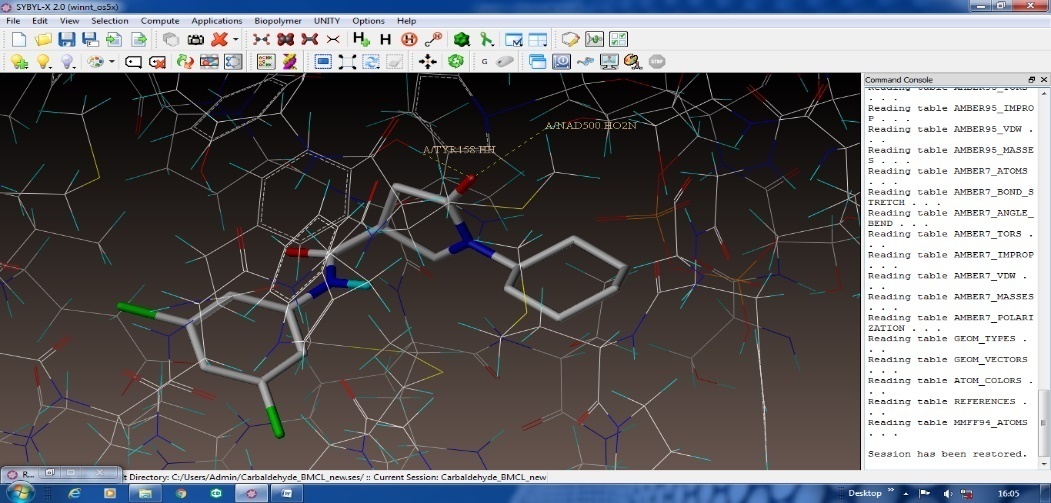


A

**
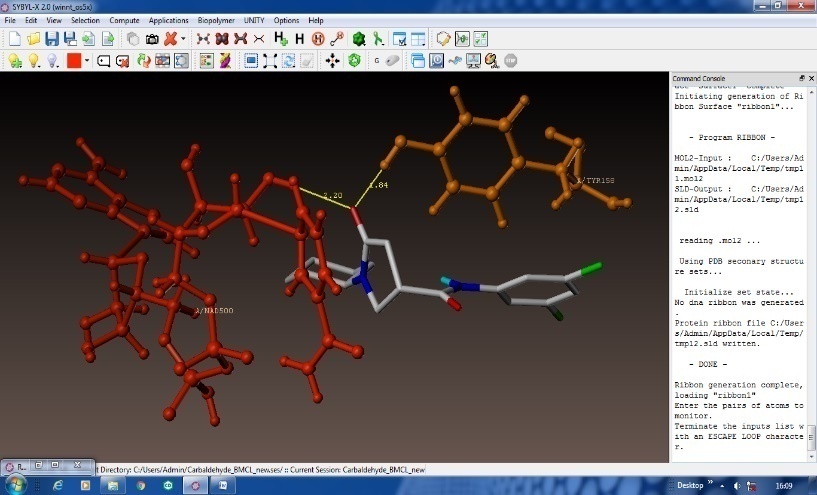
**

B


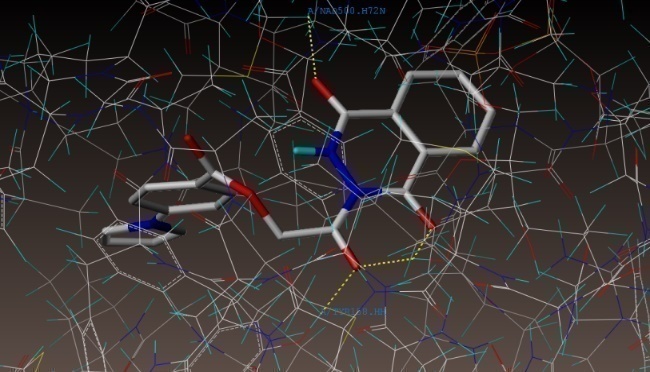
**Figure S2 (A-B):** (A) Docked mode of compound **4a**; B) 3D docked view of the compound **4a**. Binding site residues; cyan colored TYR158 amino acid, green colored co-factor NAD+ and the molecule is colored according to atom type.

A


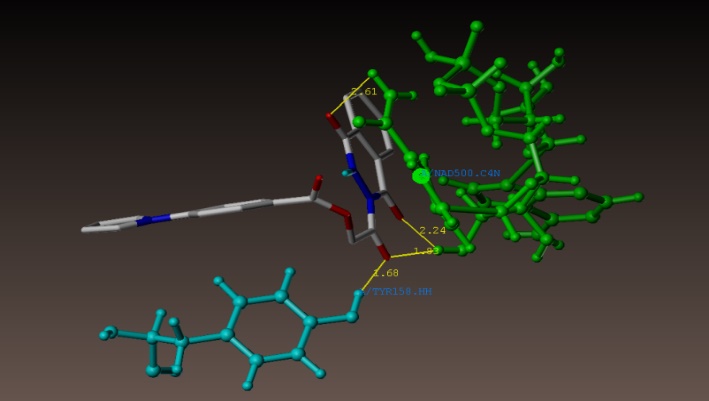


B

**Figure S3 (A-B):** (A) Docked mode of compound **5h**; (B) 3D docked view of the compound **5h**. Binding site residues; cyan colored TYR158 amino acid, green colored co-factor NAD+ and the molecule is colored according to atom type.


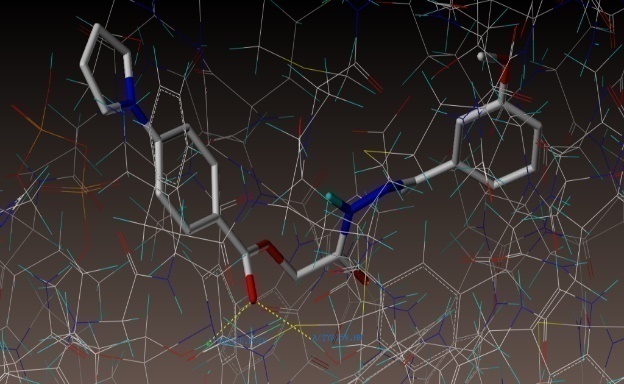


A

**
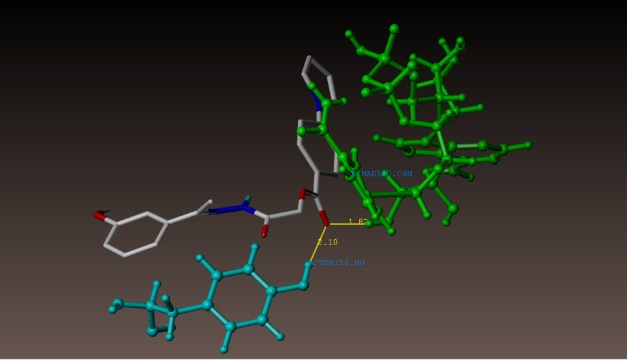
**

B


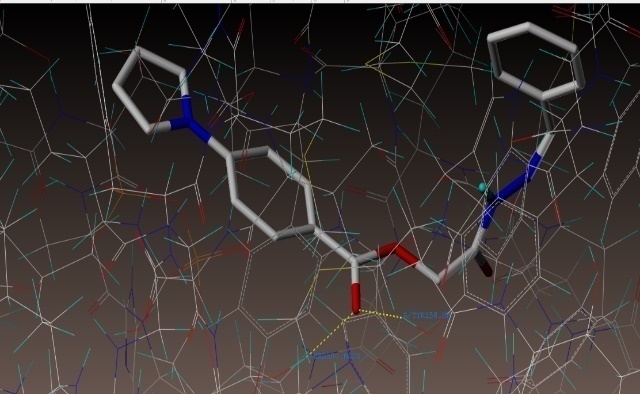
**Figure S4 (A-B):** (A) Docked mode of compound **6a**; (B) 3D docked view of the compound **6a**. Binding site residues; cyan colored TYR158 amino acid, green colored co-factor NAD+ and the molecule is colored according to atom type.

A

**
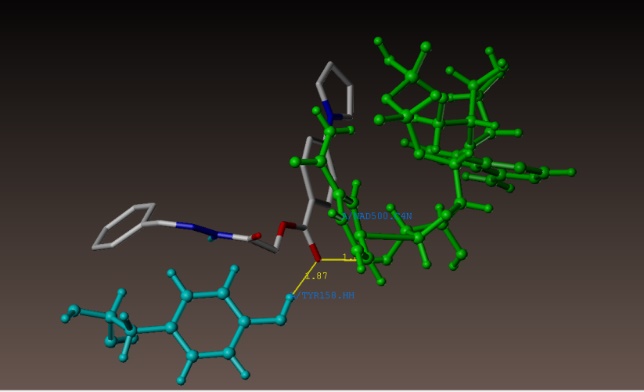
**

B


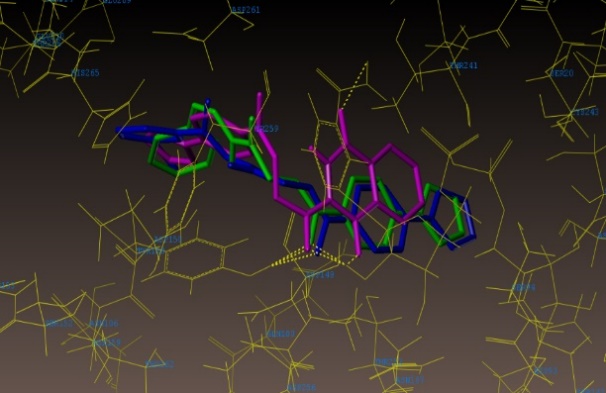
**Figure S5 (A-B):** A) Compounds **4a** (magenta colour), **5h** (blue colour) and **6a** (green colour) bordered with necessary hydrophobic amino acids. B) Hydrophilic amino acids bounded to compounds **4a, 5h**and **6a**.

A

**
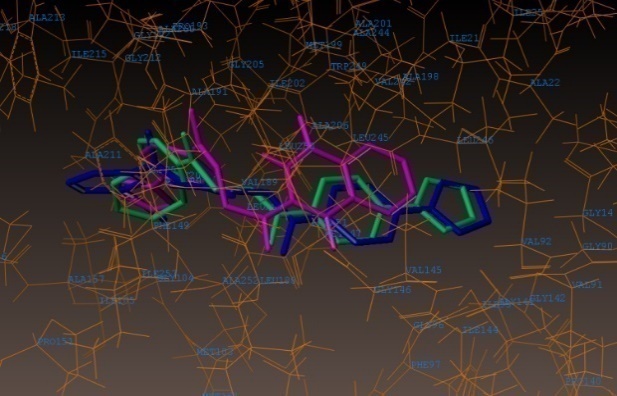
**

B

**Figure S6 (A-B):** (A) 1DF7 ligand docked mode at DHFR; (B) 3D-Docked view of 1DF7 ligand.

A


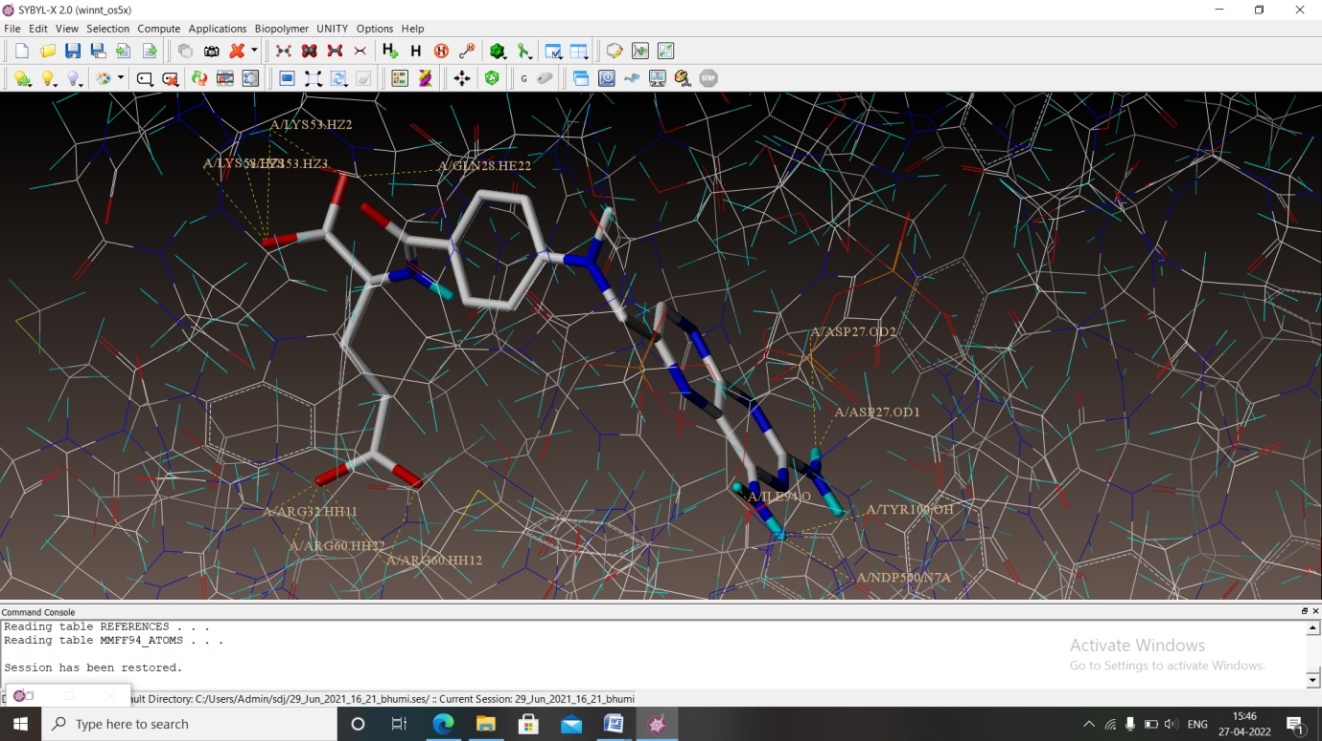


A

**
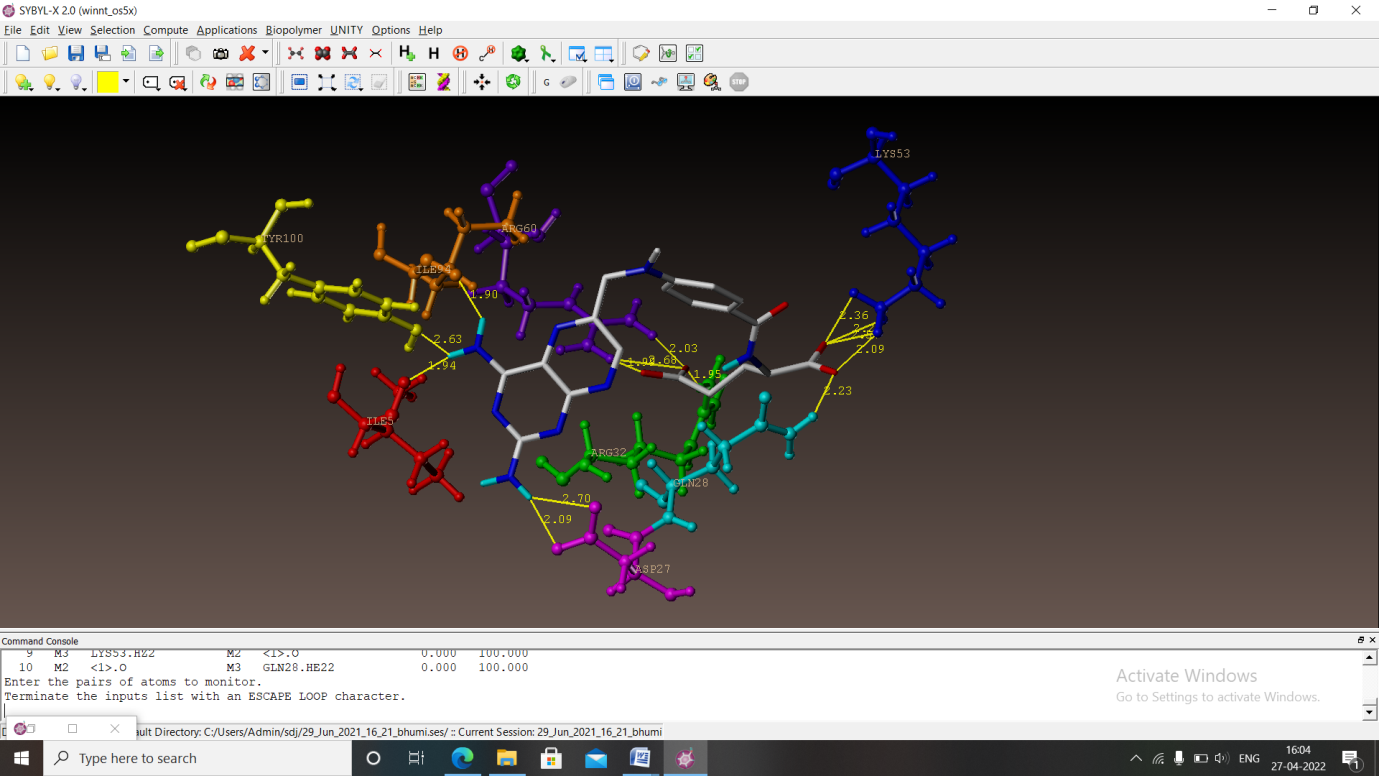
**

B

**Figure S7 (A-B):** (A) Compound **4a** docked mode; (B) Compound **4a** 3D-docked view. Binding site residues include cyan-colored ARG32 amino acid, green-colored ARG60 amino acid, and a molecule that is colored according to atom type.


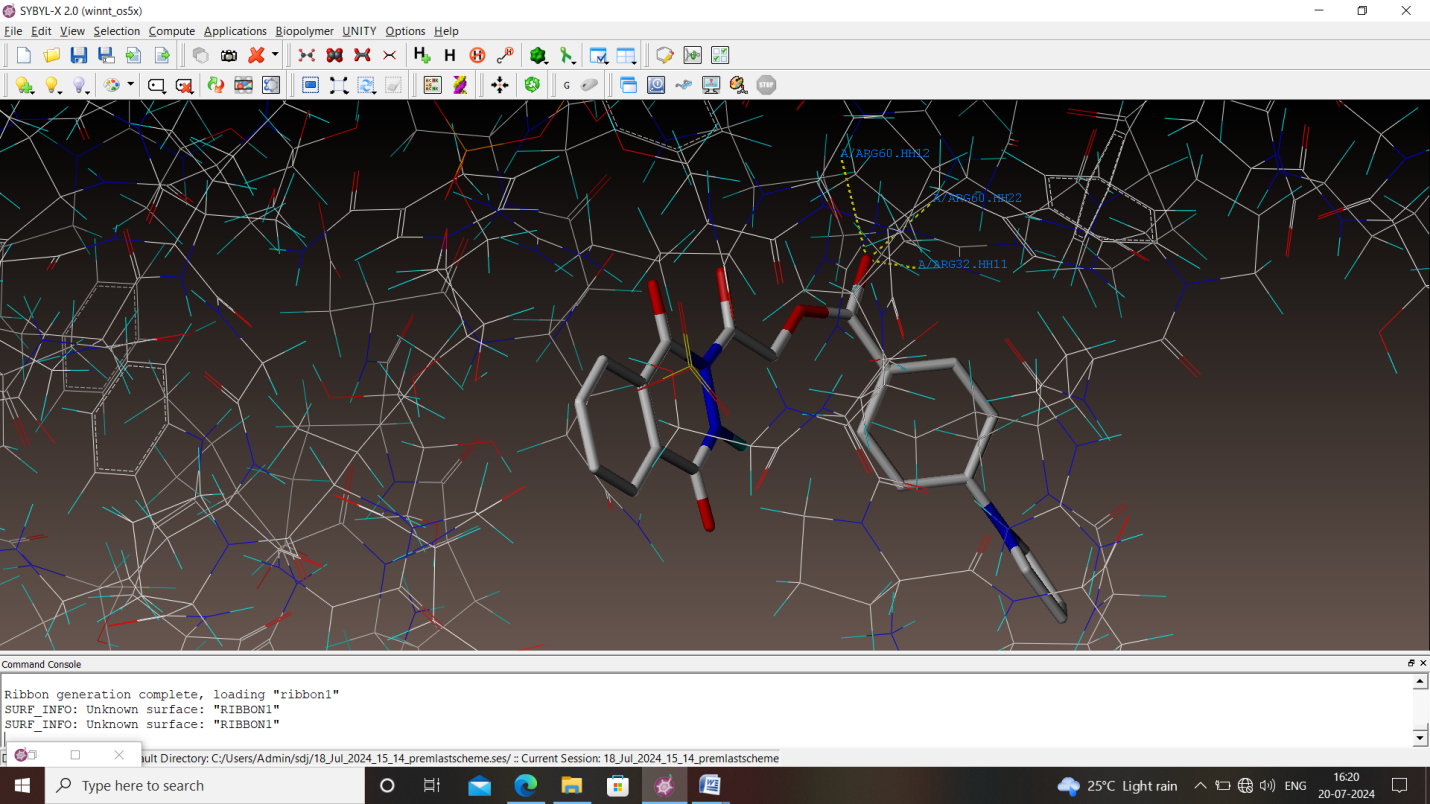


A

**
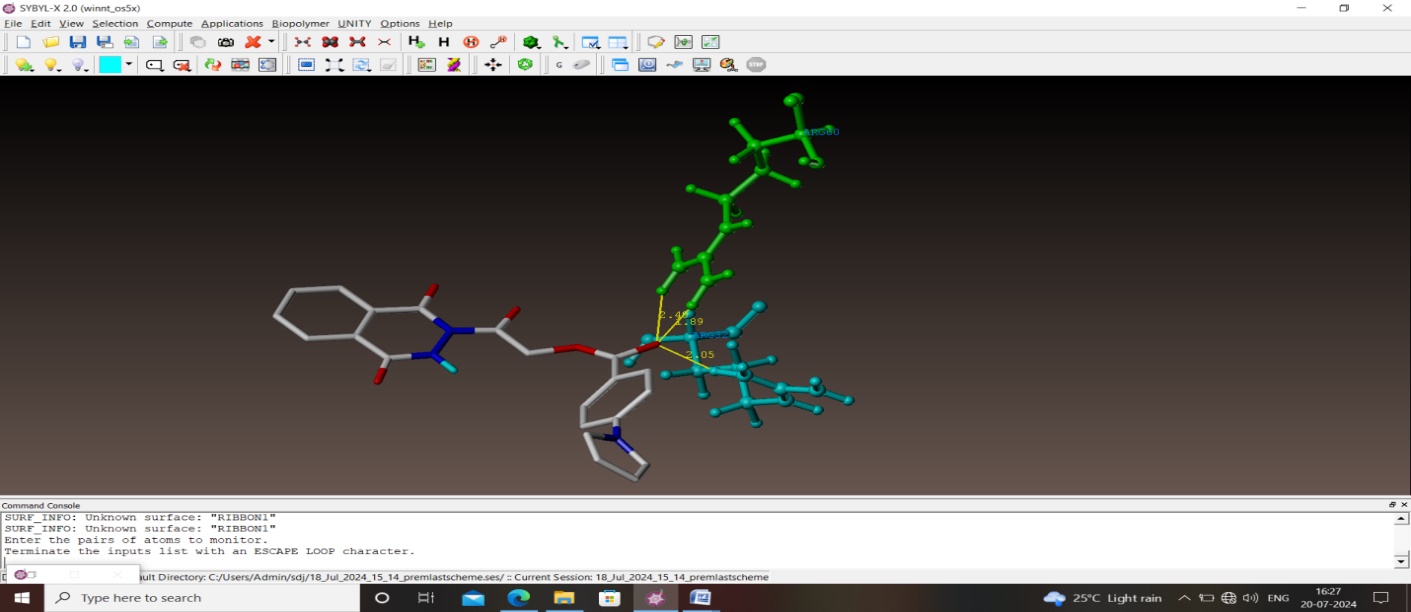
**

B

**Figure S8 (A-B):** (A) Compound 5h docked mode; (B) Compound 5h 3D-docked view. Binding site residues include cyan-colored ARG32 amino acid, green-colored ARG60 amino acid, and a molecule that is colored according to atom type.


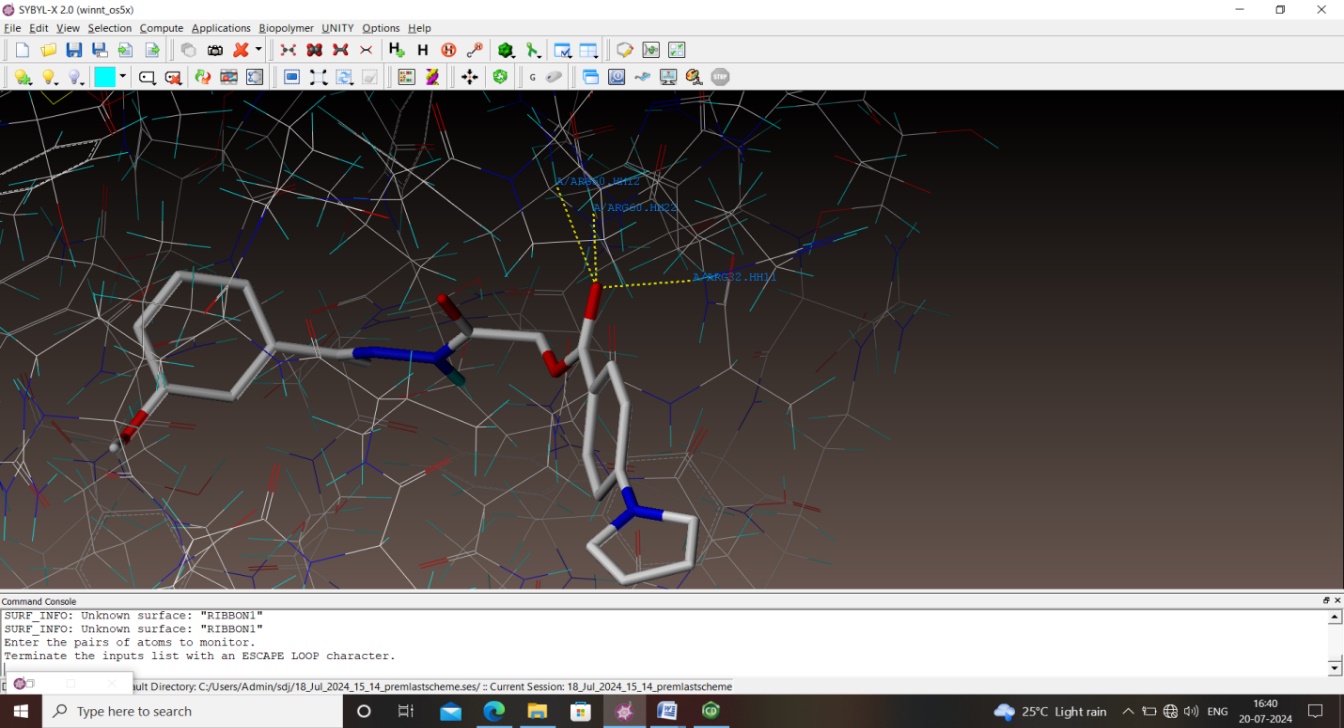


A

**
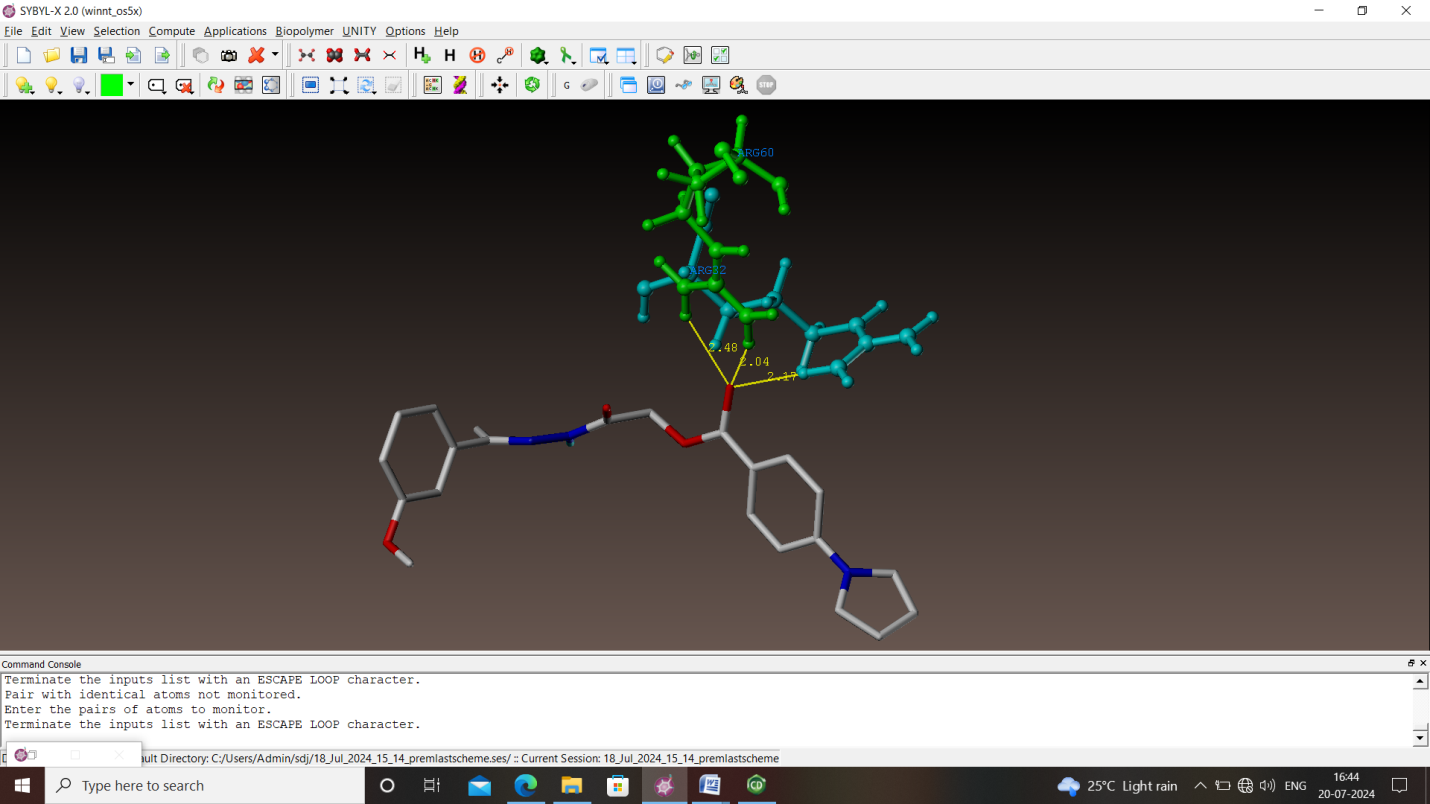
**

B

**Figure S9 (A-B):** (A) Compound **6a** docked mode; (B) Compound **6a** 3D-docked view. Binding site residues include cyan-colored ARG32 amino acid, green-colored ARG60 amino acid, and a molecule that is colored according to atom type.


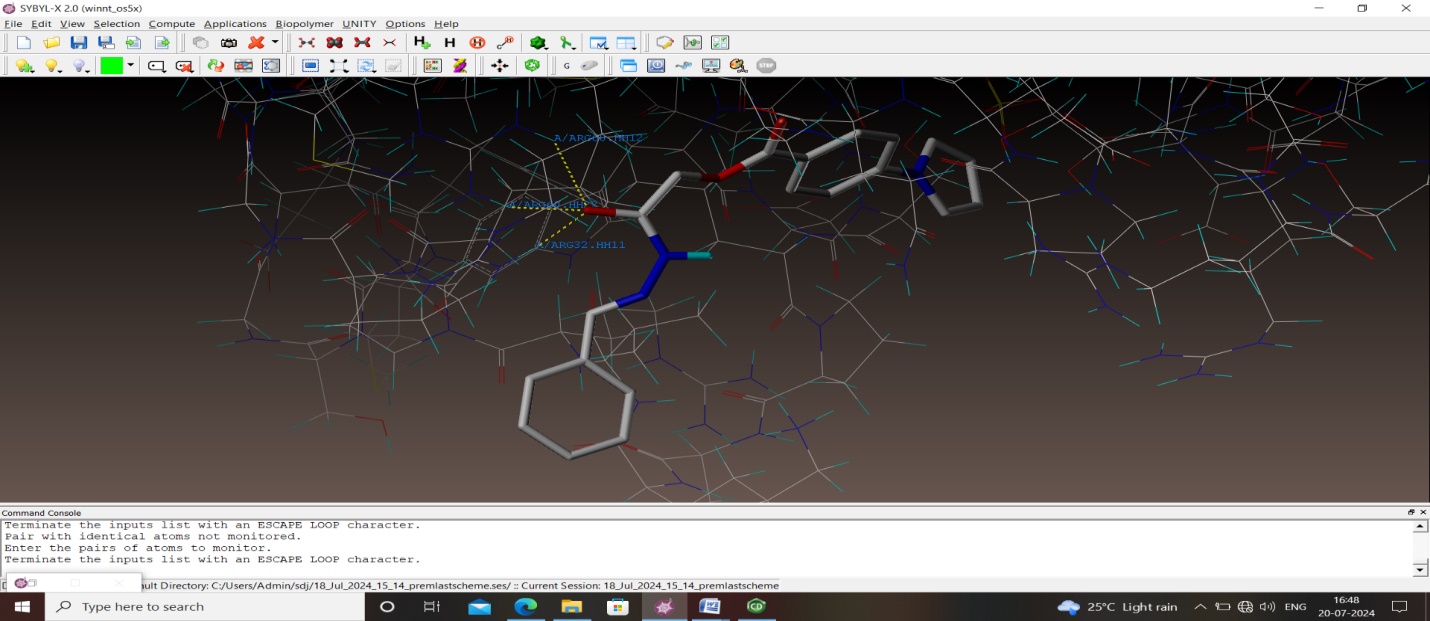


A

**
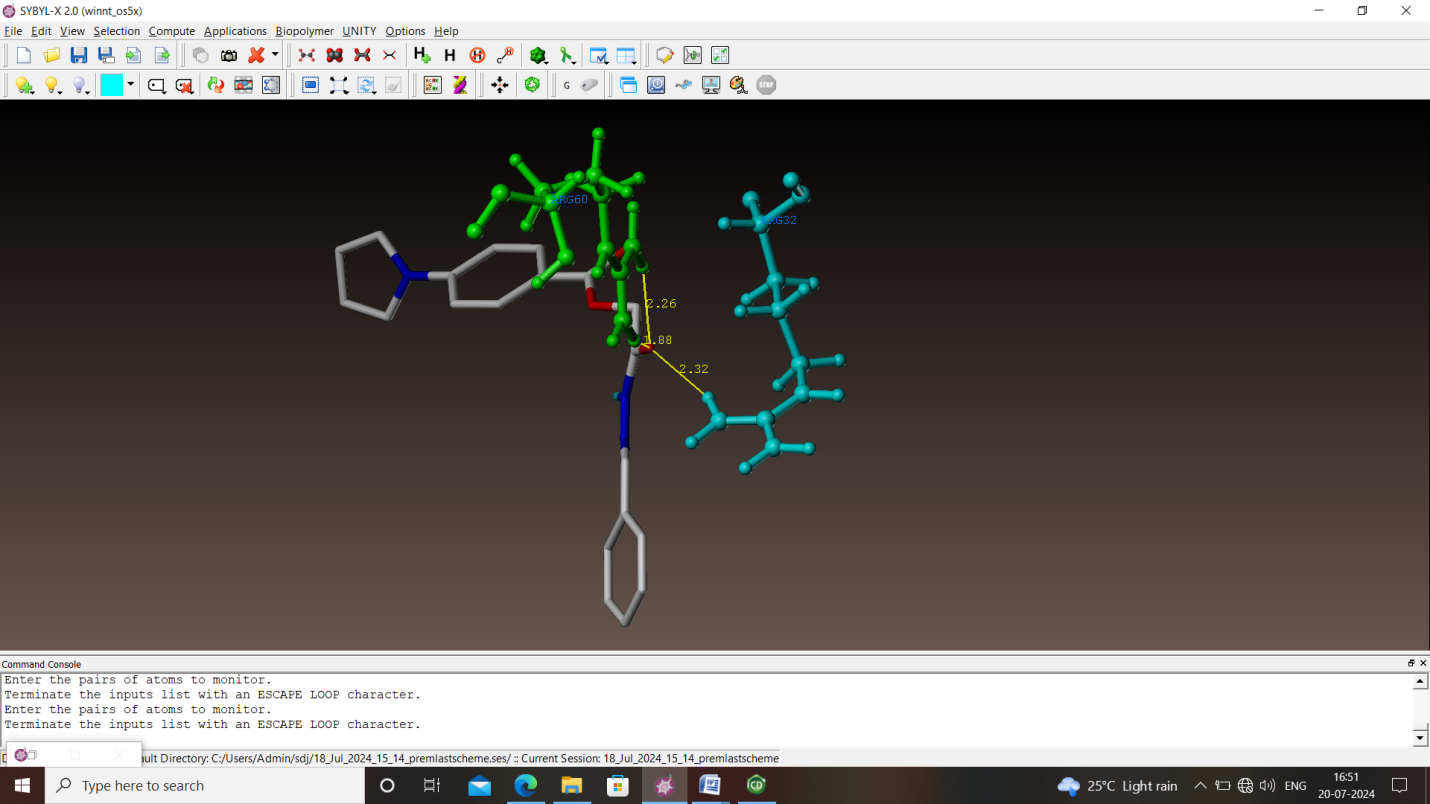
**

B

**Figure S10 (A-B):** A) Compounds **4a** (magenta colour), **5h** (blue colour) and **6a** (green colour) bordered with necessary hydrophobic amino acids. B) Hydrophilic amino acids bounded to compounds **4a, 5h** and **6a**.

**
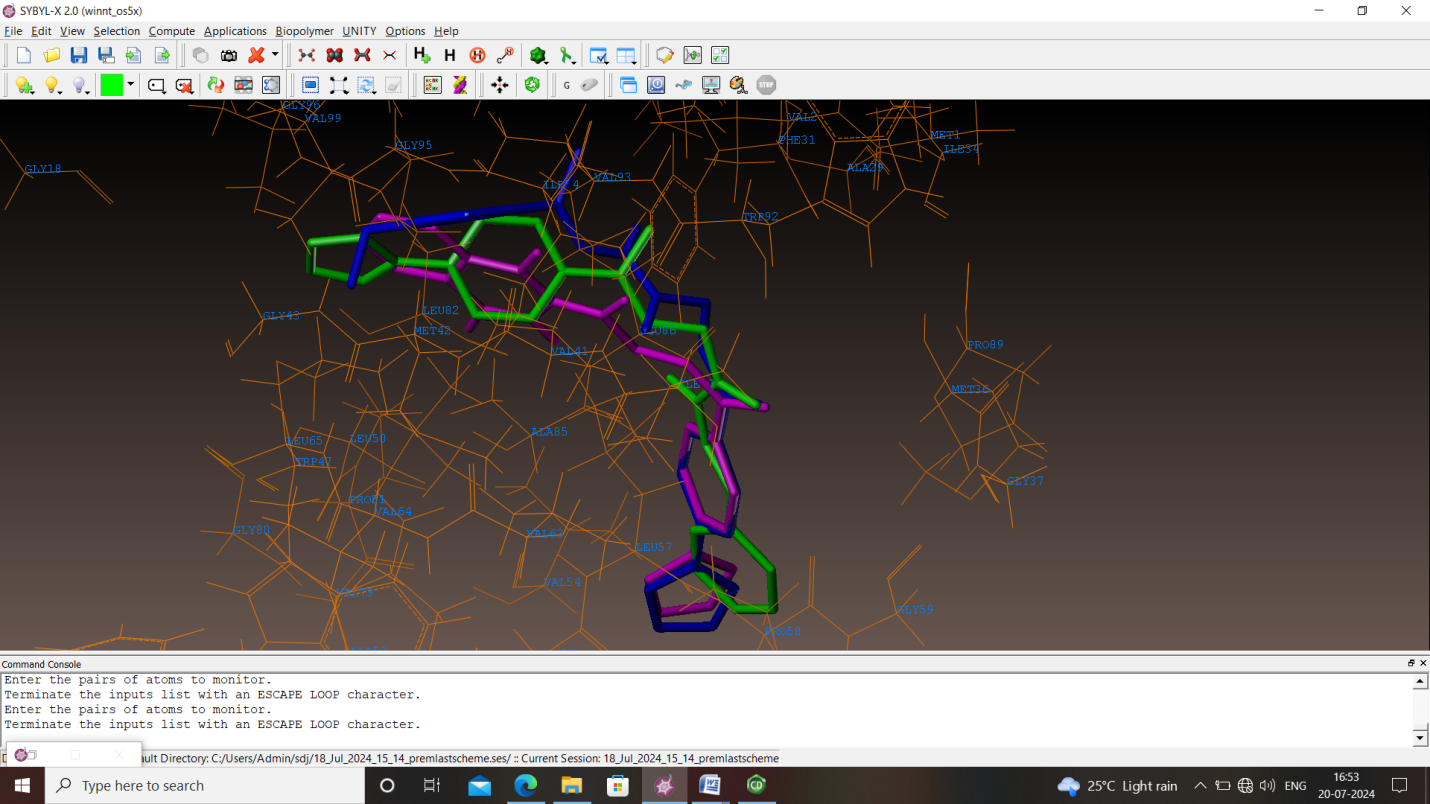
**

A

**
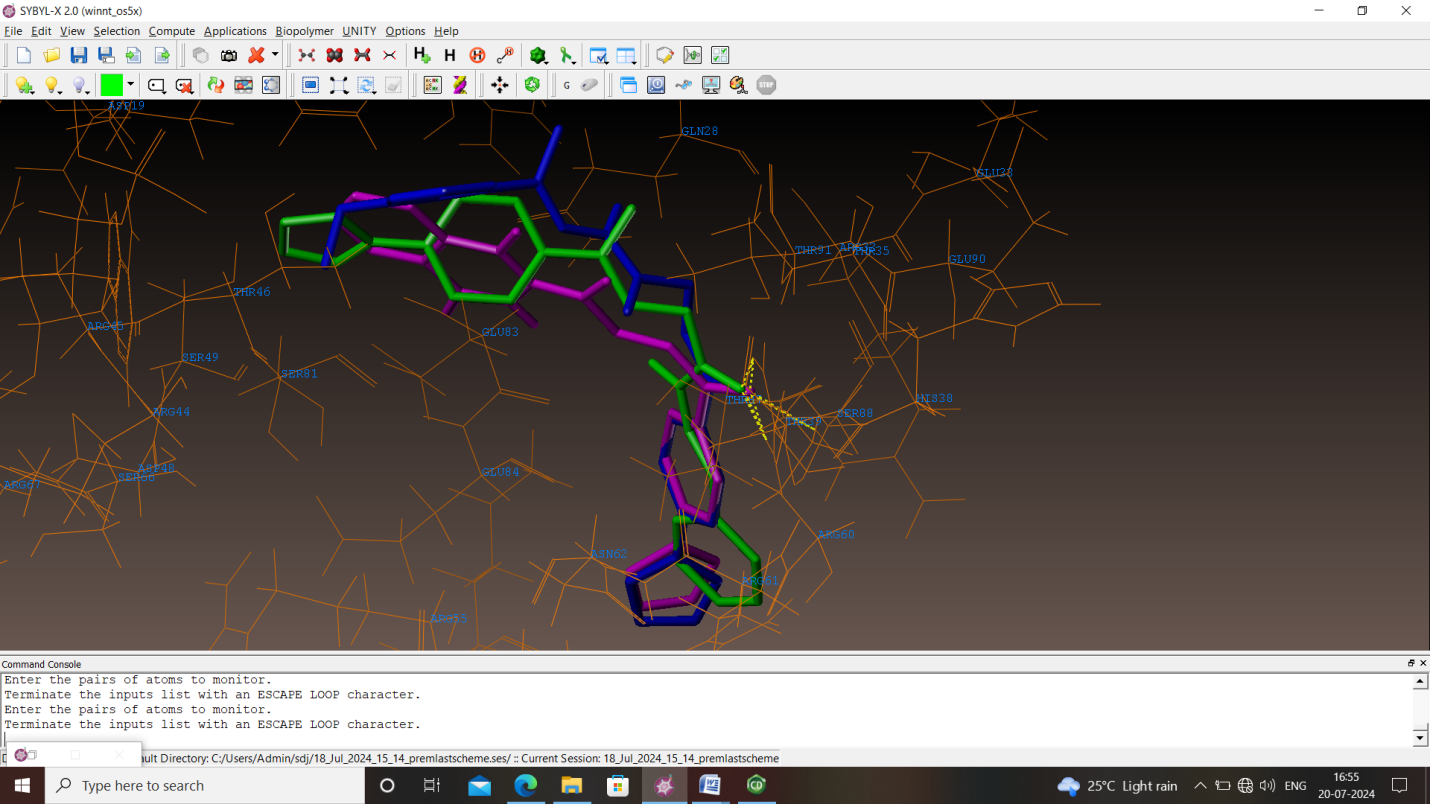
**

B

**Table S1**: Swiss ADME web tool's ADME properties for synthetic molecules:

| **Compound** | **Log P** | **Molar refractivity** | **TPSA** | **HBA** | **HBD** | **RB** | **GI Absorption** | **BBB Permeant** | **Log Kp cm/s** | **Solubility** | **CYP inhibitor** | | | | | **Lipinski violation** | **Synthetic accessibility** |
| --- | --- | --- | --- | --- | --- | --- | --- | --- | --- | --- | --- | --- | --- | --- | --- | --- | --- |
|  |  |  |  |  |  |  |  |  |  |  | **1A2** | **2C19** | **2C9** | **2D6** | **3A4** |  |  |
| **4a** | 2.27 | 105.45 | 103.16 | 5 | 1 | 6 | High | No | -6.98 | Soluble | No | Yes | Yes | No | No | 0 | 2.96 |
| **4b** | 2.35 | 125.49 | 103.16 | 5 | 1 | 6 | High | No | -6.04 | Poorly Soluble | Yes | Yes | Yes | No | No | 2 | 3.03 |
| **4c** | 2.88 | 136.25 | 103.16 | 5 | 1 | 6 | High | No | -6.94 | Poorly Soluble | Yes | Yes | No | No | No | 2 | 3.14 |
| **4d** | 2.01 | 93.10 | 97.71 | 5 | 1 | 6 | High | No | -7.93 | Soluble | No | No | No | No | No | 0 | 2.54 |
| **4e** | 2.05 | 87.94 | 103.16 | 5 | 1 | 6 | High | No | -7.68 | Soluble | No | Yes | No | No | No | 0 | 2.72 |
| **4f** | 2.23 | 92.91 | 103.16 | 5 | 1 | 6 | High | No | -7.50 | Soluble | No | Yes | No | No | No | 0 | 2.94 |
| **4g** | 2.05 | 97.43 | 97.71 | 5 | 1 | 6 | High | No | -7.56 | Soluble | No | No | No | No | No | 0 | 2.80 |
| **5a** | 3.31 | 102.64 | 72.69 | 4 | 1 | 8 | High | Yes | -6.11 | Moderately Soluble | No | Yes | Yes | No | No | 0 | 2.89 |
| **5b** | 3.77 | 110.34 | 72.69 | 4 | 1 | 8 | High | Yes | -6.10 | Moderately Soluble | Yes | Yes | Yes | No | Yes | 0 | 2.98 |
| **5c** | 3.52 | 107.65 | 72.69 | 4 | 1 | 8 | High | Yes | -5.87 | Moderately Soluble | Yes | Yes | Yes | No | Yes | 0 | 2.92 |
| **5d** | 2.89 | 111.47 | 118.51 | 6 | 1 | 9 | High | No | -6.51 | Moderately Soluble | No | Yes | Yes | No | Yes | 0 | 3.09 |
| **5e** | 3.66 | 107.61 | 72.69 | 4 | 1 | 8 | High | Yes | -5.93 | Moderately Soluble | No | Yes | Yes | No | Yes | 0 | 3.00 |
| **5f** | 3.82 | 110.34 | 72.69 | 4 | 1 | 8 | High | Yes | -6.10 | Moderately Soluble | Yes | Yes | Yes | No | Yes | 0 | 3.04 |
| **5g** | 2.86 | 111.47 | 118.51 | 6 | 1 | 9 | High | No | -6.51 | Moderately Soluble | No | Yes | Yes | No | Yes | 0 | 3.14 |
| **5h** | 3.15 | 109.14 | 81.92 | 5 | 1 | 9 | High | No | -6.32 | Moderately Soluble | No | Yes | Yes | No | Yes | 0 | 3.03 |
| **5i** | 3.57 | 109.14 | 81.92 | 5 | 1 | 9 | High | No | -6.32 | Moderately Soluble | No | Yes | Yes | No | Yes | 0 | 2.99 |
| **5j** | 2.95 | 104.67 | 92.92 | 5 | 2 | 8 | High | No | -6.46 | Moderately Soluble | No | No | Yes | No | No | 0 | 2.90 |
| **5k** | 3.69 | 107.65 | 72.69 | 4 | 1 | 8 | High | Yes | -5.87 | Moderately Soluble | Yes | Yes | Yes | No | Yes | 0 | 2.93 |
| **5l** | 3.36 | 102.60 | 72.69 | 5 | 1 | 8 | High | Yes | -6.15 | Moderately Soluble | No | Yes | Yes | No | Yes | 0 | 2.91 |
| **5m** | 3.91 | 118.04 | 72.69 | 4 | 1 | 8 | High | No | -6.09 | Moderately Soluble | Yes | Yes | Yes | No | Yes | 0 | 3.11 |
| **5n** | 3.62 | 112.66 | 72.69 | 4 | 1 | 8 | High | Yes | -5.64 | Moderately Soluble | Yes | Yes | Yes | No | Yes | 0 | 3.06 |
| **5o** | 2.96 | 107.05 | 98.71 | 4 | 2 | 8 | High | No | -6.69 | Soluble | No | No | Yes | No | No | 0 | 2.97 |
| **5p** | 2.93 | 104.67 | 92.92 | 5 | 2 | 8 | High | No | -6.46 | Moderately Soluble | No | No | Yes | No | No | 0 | 2.91 |
| **6a** | 3.17 | 97.84 | 72.69 | 4 | 1 | 8 | High | Yes | -6.18 | Soluble | No | Yes | Yes | No | Yes | 0 | 2.83 |
| **6b** | 3.50 | 105.54 | 72.69 | 4 | 1 | 8 | High | Yes | -6.17 | Moderately Soluble | Yes | Yes | Yes | No | Yes | 0 | 2.93 |
| **6c** | 2.67 | 106.66 | 118.51 | 6 | 1 | 9 | High | No | -6.58 | Soluble | No | Yes | Yes | No | Yes | 0 | 3.02 |
| **6d** | 3.27 | 102.85 | 72.69 | 4 | 1 | 8 | High | Yes | -5.95 | Moderately Soluble | Yes | Yes | Yes | No | No | 0 | 2.85 |
| **6e** | 3.21 | 104.33 | 81.92 | 5 | 1 | 9 | High | No | -6.39 | Moderately Soluble | No | Yes | Yes | No | Yes | 0 | 2.91 |

**Table S2**: Toxicity studies of synthesized compounds:

| **Compound code** | **LD50**  **mg/kg** | **Hepatotoxicity** | **Carcinogenicity** | **Immunotoxicity** | **Mutagenicity** | **Cytotoxicity** | **Aryl hydrocarbon Receptor** | **Androgen Receptor (AR)** | **Androgen Receptor Ligand Binding Domain** | **Aromatase** | **Estrogen Receptor Ligand Binding Domain** | **Peroxisome Proliferator Activated Receptor Gamma** | **Nuclear factor** | **Heat shock factor response element** | **Mitochondrial Membrane Potential** | **Phosphoprotein** | **ATPase family AAA domain containing protein 5** |
| --- | --- | --- | --- | --- | --- | --- | --- | --- | --- | --- | --- | --- | --- | --- | --- | --- | --- |
| **4a** | 750 | Active | Inactive | Inactive | Inactive | Inactive | Inactive | Inactive | Inactive | Inactive | Inactive | Inactive | Inactive | Inactive | Inactive | Inactive | Inactive |
| **4b** | 750 | Active | Inactive | Inactive | Inactive | Inactive | Inactive | Inactive | Inactive | Inactive | Inactive | Inactive | Inactive | Inactive | Inactive | Inactive | Inactive |
| **4c** | 750 | Active | Inactive | Inactive | Inactive | Inactive | Inactive | Inactive | Inactive | Inactive | Inactive | Inactive | Inactive | Inactive | Inactive | Inactive | Inactive |
| **4d** | 5000 | Inactive | Inactive | Inactive | Inactive | Inactive | Inactive | Inactive | Inactive | Inactive | Inactive | Inactive | Inactive | Inactive | Inactive | Inactive | Inactive |
| **4e** | 750 | Active | Inactive | Inactive | Inactive | Inactive | Inactive | Inactive | Inactive | Inactive | Inactive | Inactive | Inactive | Inactive | Inactive | Inactive | Inactive |
| **4f** | 540 | Active | Inactive | Inactive | Inactive | Inactive | Inactive | Inactive | Inactive | Inactive | Inactive | Inactive | Inactive | Inactive | Inactive | Inactive | Inactive |
| **4g** | 2300 | Active | Inactive | Inactive | Inactive | Inactive | Inactive | Inactive | Inactive | Inactive | Inactive | Inactive | Inactive | Inactive | Inactive | Inactive | Inactive |
| **5a** | 1000 | Active | Active | Inactive | Inactive | Inactive | Inactive | Inactive | Inactive | Inactive | Inactive | Inactive | Inactive | Inactive | Inactive | Inactive | Inactive |
| **5b** | 1000 | Active | Inactive | Inactive | Inactive | Inactive | Inactive | Inactive | Inactive | Inactive | Inactive | Inactive | Inactive | Inactive | Inactive | Inactive | Inactive |
| **5c** | 1830 | Active | Inactive | Inactive | Inactive | Inactive | Inactive | Inactive | Inactive | Inactive | Inactive | Inactive | Inactive | Inactive | Inactive | Inactive | Inactive |
| **5d** | 5000 | Active | Active | Inactive | Active | Inactive | Inactive | Inactive | Inactive | Inactive | Inactive | Inactive | Inactive | Inactive | Inactive | Inactive | Inactive |
| **5e** | 1000 | Active | Active | Inactive | Inactive | Inactive | Inactive | Inactive | Inactive | Inactive | Inactive | Inactive | Inactive | Inactive | Inactive | Inactive | Inactive |
| **5f** | 1000 | Active | Inactive | Inactive | Inactive | Inactive | Inactive | Inactive | Inactive | Inactive | Inactive | Inactive | Inactive | Inactive | Inactive | Inactive | Inactive |
| **5g** | 3000 | Active | Active | Inactive | Active | Inactive | Inactive | Inactive | Inactive | Inactive | Inactive | Inactive | Inactive | Inactive | Inactive | Inactive | Inactive |
| **5h** | 4920 | Active | Inactive | Inactive | Inactive | Inactive | Inactive | Inactive | Inactive | Inactive | Inactive | Inactive | Inactive | Inactive | Inactive | Inactive | Inactive |
| **5i** | 1120 | Active | Inactive | Inactive | Inactive | Inactive | Inactive | Inactive | Inactive | Inactive | Inactive | Inactive | Inactive | Inactive | Inactive | Inactive | Inactive |
| **5j** | 1000 | Active | Inactive | Inactive | Inactive | Inactive | Inactive | Inactive | Inactive | Inactive | Inactive | Inactive | Inactive | Inactive | Inactive | Inactive | Inactive |
| **5k** | 1830 | Active | Inactive | Inactive | Inactive | Inactive | Inactive | Inactive | Inactive | Inactive | Inactive | Inactive | Inactive | Inactive | Inactive | Inactive | Inactive |
| **5l** | 3000 | Active | Inactive | Inactive | Inactive | Inactive | Inactive | Inactive | Inactive | Inactive | Inactive | Inactive | Inactive | Inactive | Inactive | Inactive | Inactive |
| **5m** | 1000 | Active | Inactive | Inactive | Inactive | Inactive | Inactive | Inactive | Inactive | Inactive | Inactive | Inactive | Inactive | Inactive | Inactive | Inactive | Inactive |
| **5n** | 1830 | Active | Inactive | Inactive | Inactive | Inactive | Inactive | Inactive | Inactive | Inactive | Inactive | Inactive | Inactive | Inactive | Inactive | Inactive | Inactive |
| **5o** | 710 | Active | Inactive | Inactive | Inactive | Inactive | Inactive | Inactive | Inactive | Inactive | Inactive | Inactive | Inactive | Inactive | Inactive | Inactive | Inactive |
| **5p** | 4540 | Active | Inactive | Inactive | Inactive | Inactive | Inactive | Inactive | Inactive | Inactive | Inactive | Inactive | Inactive | Inactive | Inactive | Inactive | Inactive |
| **6a** | 3000 | Active | Active | Inactive | Inactive | Inactive | Inactive | Inactive | Inactive | Inactive | Inactive | Inactive | Inactive | Inactive | Inactive | Inactive | Inactive |
| **6b** | 3000 | Active | Inactive | Inactive | Inactive | Inactive | Inactive | Inactive | Inactive | Inactive | Inactive | Inactive | Inactive | Inactive | Inactive | Inactive | Inactive |
| **6c** | 5000 | Active | Active | Inactive | Active | Inactive | Inactive | Inactive | Inactive | Inactive | Inactive | Inactive | Inactive | Inactive | Inactive | Inactive | Inactive |
| **6d** | 5000 | Active | Inactive | Inactive | Inactive | Inactive | Inactive | Inactive | Inactive | Inactive | Inactive | Inactive | Inactive | Inactive | Inactive | Inactive | Inactive |
| **6e** | 1120 | Active | Inactive | Inactive | Inactive | Inactive | Inactive | Inactive | Inactive | Inactive | Inactive | Inactive | Inactive | Inactive | Inactive | Inactive | Inactive |
